# The minimal intrinsic stochasticity of constitutively expressed eukaryotic genes is sub-Poissonian

**DOI:** 10.1126/sciadv.adh5138

**Published:** 2023-08-09

**Authors:** Douglas E. Weidemann, James Holehouse, Abhyudai Singh, Ramon Grima, Silke Hauf

**Affiliations:** ^1^ Department of Biological Sciences, Virginia Tech, Blacksburg, VA 24061, USA.; ^2^ Fralin Life Sciences Institute, Virginia Tech, Blacksburg, VA 24061, USA.; ^3^ The Santa Fe Institute, 1399 Hyde Park Road, Santa Fe, NM 87510, USA.; ^4^ Department of Electrical and Computer Engineering, University of Delaware, Newark, DE 19716, USA.; ^5^ Department of Biomedical Engineering, University of Delaware, Newark, DE 19716, USA.; ^6^ School of Biological Sciences, University of Edinburgh, Edinburgh EH9 3JR, UK.

## Abstract

Gene expression inherently gives rise to stochastic variation (“noise”) in the production of gene products. Minimizing noise is crucial for ensuring reliable cellular functions. However, noise cannot be suppressed below a certain intrinsic limit. For constitutively expressed genes, this limit is typically assumed to be Poissonian noise, wherein the variance in mRNA numbers is equal to their mean. Here, we demonstrate that several cell division genes in fission yeast exhibit mRNA variances significantly below this limit. The reduced variance can be explained by a gene expression model incorporating multiple transcription and mRNA degradation steps. Notably, in this sub-Poissonian regime, distinct from Poissonian or super-Poissonian regimes, cytoplasmic noise is effectively suppressed through a higher mRNA export rate. Our findings redefine the lower limit of eukaryotic gene expression noise and uncover molecular requirements for achieving ultralow noise, which is expected to be important for vital cellular functions.

## Teaser

Eukaryotic genes can have expression noise below the commonly assumed noise floor.

## INTRODUCTION

All gene expression is “noisy.” Gene products fluctuate stochastically, which is an inescapable consequence of the nature of gene expression (*1*, *2*). Gene expression, like other intracellular processes, relies on molecule-molecule (in the case of gene expression often protein-DNA) interactions. When both molecules in such a reaction are abundant, their interaction is frequent and not limiting for the reaction itself. However, for several stages of gene expression, one binding partner may be present in very low numbers. For example, to initiate transcription, proteins must bind a gene promoter, which typically only has one to four copies per cell. As a result, stochasticity becomes apparent and the intracellular number of mRNA molecules that have been transcribed from a specific gene varies substantially over time and between cells (*3*–*5*).

Gene expression noise differs between genes. For genes with high noise, some cells in the population may contain dozens or hundreds of mRNA molecules expressed from that gene, and others none (*4*, *6*–*8*). In a few cases, this large variation has been shown to be advantageous since it allows “bet-hedging,” i.e., some cells in the population are well prepared for a change in environmental conditions, whereas others do not produce gene products that are not currently needed (*9*). Highly variable expression of a single gene is also exploited in development, where it may specify cell fate and generate a mix of cells with different phenotypes (*1*, *2*). At the other end of the spectrum, “housekeeping” genes, i.e., genes required for core cellular processes and often required for survival, are expressed stably with low noise (*10*, *11*). Cell cycle genes also show low intrinsic noise (*12*).

High-noise regimes have been extensively studied. One contributing factor to noisy expression is toggling between an inactive and active transcription state so that mRNAs are produced in “bursts,” leading to large variations in mRNA abundance (*13*). The large noise created by bursty expression can be modulated downstream of mRNA synthesis. For example, slow mRNA export from the nucleus may buffer bursty mRNA synthesis and thereby lower noise in the cytoplasm relative to noise in the nucleus (*7*, *14*). By contrast, molecular processes downstream of mRNA synthesis, such as multistep mRNA decay, may enhance noise created during transcription (*15*).

Less is known about the low-noise regime. Low-noise genes are thought to be expressed from constitutive promoters that stay “on” and do not toggle into an “off” state (*2*, *16*). Widely used models for this constitutive expression assume that mRNA is produced with a single rate-limiting step from an active promoter and that the wait time between synthesis events follows an exponential distribution (*17*, *18*). This has been experimentally confirmed for two constitutively expressed genes in budding yeast (*19*). Under these assumptions, the steady-state mRNA numbers in the cell population will follow a Poisson distribution, independent of the precise nature of the mRNA degradation process (*20*, *21*). A Poisson distribution is characterized by the variance being equal to the mean, or the Fano factor (the variance divided by the mean) being equal to 1. Consistent with this proposed expression model, several constitutively expressed genes in yeast show distributions of mRNA numbers that closely follow a Poisson distribution (*3*, *8*, *22*, *23*). This distribution is typically considered the “noise floor,” i.e., the lower limit of intrinsic stochasticity in gene expression (*7*, *23*–*25*).

Several theoretical models have examined how noise can be minimized, for example, by negative feedback loops or by wait times between synthesis events that follow a narrower than exponential distribution (*21*, *26*–*33*). Despite the theoretical possibility of achieving cytoplasmic mRNA numbers with a sub-Poissonian distribution (Fano factor < 1), reports of such narrow distributions within cell populations are extremely sparse. To our knowledge, there are only isolated reports from bacteria (*28*, *34*, *35*) and hints for single genes in eukaryotes (*23*, *36*) without well-controlled confirmation. Sub-Poissonian distributions of mRNA numbers have been reported for yeast transcription sites (TSs) (*22*, *30*), but these sites are distinct in that they contain mRNA molecules of varying lengths in the process of being transcribed. As a result, the sub-Poissonian distribution at the TS can be consistent with a classical Poissonian gene expression model (*37*).

To shed additional light on low-noise gene expression, we have investigated a group of constitutively expressed fission yeast (*Schizosaccharomyces pombe*) genes that are important for cell division and show low mRNA and protein noise (*38*, *39*). The protein products of these genes contribute to the spindle assembly checkpoint (SAC, also known as mitotic checkpoint). This signaling pathway operates during cell division to detect chromosomes that are not correctly attached to the mitotic spindle and halts the execution of anaphase as a response (*40*, *41*). SAC signaling involves several protein-protein interactions, and the relative ratio of SAC proteins is important for SAC function (*38*, *42*, *43*). Hence, low expression noise of these genes supports SAC function, making it important to understand how low noise is achieved.

Notably, rather than Poissonian mRNA distributions, we found sub-Poissonian mRNA distributions for these genes, i.e., Fano factors less than 1. In the cytoplasm, the Fano factor could be as low as 0.5. We also examined other, non-SAC, low-noise genes, re-examined published results, and found a spectrum of sub-Poissonian to Poissonian mRNA distributions for constitutively expressed genes. This suggests that constitutively expressed genes are not a homogenous group with respect to mRNA noise, but instead can differ substantially in the extent of their noise. We conclusively establish that the lower limit of intrinsic stochasticity for constitutively expressed eukaryotic genes is sub-Poissonian, not Poissonian.

## RESULTS

### Gene expression is regulated independently for *S. pombe* SAC genes

**Fig. 1.**
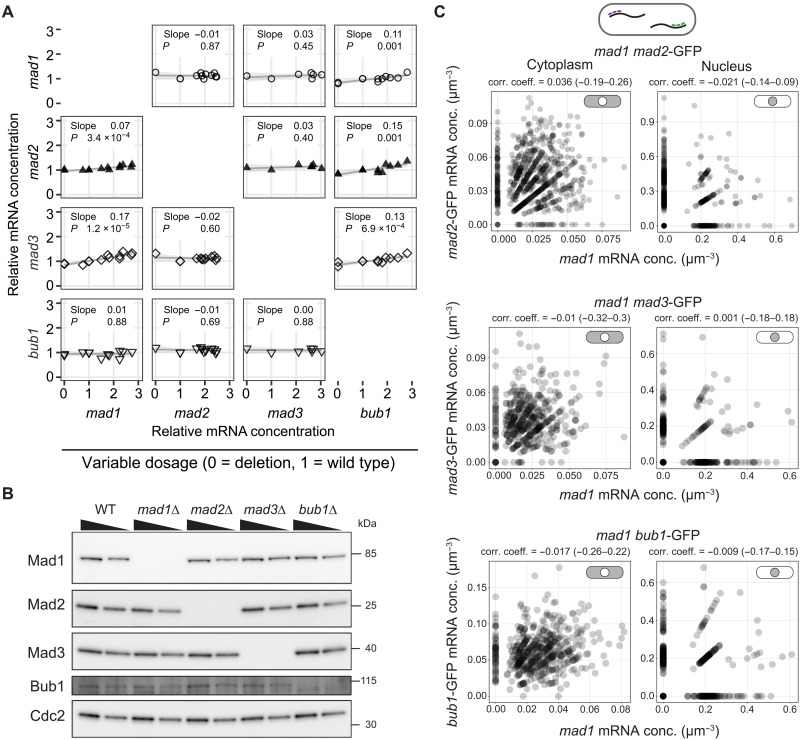
The expression of individual spindle assembly checkpoint (SAC) genes is not correlated with other SAC genes. (**A**) Strains in which SAC genes were either deleted (dosage = 0) or slightly overexpressed by expression from a second genetic locus (dosage >1) were analyzed for the concentration of SAC gene mRNAs by quantitative polymerase chain reaction (qPCR); the mRNA concentration is given relative to that in a wild-type strain (dosage = 1). Parameters of linear regression are shown. (**B**) Immunoblot of cell extracts from the indicated strains; 70% of the extract is loaded in each second lane. Antibodies targeted the endogenous proteins. Cdc2 serves as a loading control. (**C**) The mRNA concentrations of *mad1* and one other SAC gene, tagged with green fluorescent protein (GFP), were determined by single-molecule mRNA fluorescence in situ hybridization (smFISH) (*n* = 734 cells for *mad1*/*mad2*-GFP, 451 cells for *mad1*/*mad3*-GFP, 502 cells for *mad1*/*bub1*-GFP; one experiment each). Cells expressed the targeted SAC genes from their endogenous locus; *mad1* mRNA was detected with *mad1*-specific probes and the other mRNAs with probes against GFP. The correlation coefficient was calculated as Kendall’s tau b; the 95% confidence interval is given in brackets.

SAC genes need to cooperate in one signaling pathway, and their relative stoichiometries are important for function (*38*, *42*, *43*). Stoichiometries could be more easily maintained if the expression of these genes was coupled, i.e., if higher or lower expression of one coincided with higher or lower expression of another. Yet, isolated observations suggest that their expression is independent (*38*, *44*). We systematically tested for interdependence at both the mRNA and protein levels (Fig. 1). When single SAC genes were deleted, the mRNA levels measured for other SAC genes by quantitative polymerase chain reaction (qPCR) remained close to the level observed in wild-type cells (Fig. 1A), suggesting that there is no direct feedback on the expression of other SAC genes. Large changes were also not observed on the protein level, either in single or double deletions (Fig. 1B and fig. S8, A to C). A slight overexpression from integrating a second copy of each SAC gene at an exogenous locus also did not greatly change the mRNA or protein levels of other SAC genes (Fig. 1A and fig. S8). We did observe a slight increase in mRNA concentration of *mad1*, *mad2*, and *mad3* by qPCR when expression of *bub1* was increased by two- to threefold, and a similar trend for *mad3* when *mad1* was increased by two- to threefold (Fig. 1A). However, the changes were subtle and not readily detectable on the protein level (fig. S8, D to G). Together, this suggests that either deleting or doubling the expression of a single SAC gene does not greatly alter the expression of other SAC genes. An alternative possibility for coregulation is a common upstream regulator. In such a scenario, the mRNA concentration of coregulated genes in single cells should correlate. We were particularly interested in whether this might be the case for Mad1 and Mad2, which form a tight 2:2 complex that is central to the SAC function (*40*). However, *mad1* and *mad2* mRNA concentrations did not correlate in single cells (Fig. 1C). This agrees with findings in budding yeast that the concentrations of constitutively expressed mRNAs coding for subunits of stable protein complexes do not necessarily correlate (*22*). We also did not observe any correlation in the mRNA concentrations of *mad1* and *mad3*, or *mad1* and *bub1* (Fig. 1C). Overall, we conclude that *S. pombe* SAC genes are likely expressed independently of each other.

### SAC genes show sub-Poissonian distributions of mRNA numbers narrower than those of other low-noise genes

Without coordination between SAC expression levels, it is important that the expression of these genes is stable so that their levels do not deviate beyond the stoichiometric range that allows for a functional SAC. We therefore assessed variability in SAC gene mRNA numbers at the single-cell level by single-molecule mRNA fluorescence in situ hybridization, smFISH (*45*). The spot intensities in the cytoplasm were homogenously distributed in a narrow range, consistent with each spot representing a single mRNA molecule (fig. S9). Spots with higher intensities were almost exclusively observed for highly expressed genes and only in the nucleus, presumably representing the TS (figs. S9 and S18C). To determine the number of mRNAs as accurately as possible, we assessed three different methods. (i) We counted the number of FISH spots per cell while ignoring their intensity (“spot count”). While this provides accurate estimates of mRNA number for weakly expressed genes, it underestimates both number and variability when a substantial fraction of spots contains more than one mRNA molecule. (ii) Conversely, to take the intensity of spots fully into account, we determined the number of mRNA molecules by summing up the normalized intensities of all spots (“intensity count”). This comes with the drawback of summing up errors from technical variation in intensity (e.g., from uneven labeling or uneven intensity across the image). Hence, it causes the variability in mRNA counts to be overestimated (see Materials and Methods for more details). For most analyses, therefore, we used (iii) a hybrid approach to quantifying mRNA per cell (fig. S9C). If spots had intensities below the 95th percentile of spot intensities in the cytoplasm, then we counted them as single mRNA molecules. For brighter spots, the number of mRNAs contained in each spot was estimated as the value of its intensity divided by the median intensity of a cytoplasmic spot. This approach (“hybrid count”) minimizes technical noise in intensities from being interpreted as biological noise while still ensuring that several mRNAs at the same position are accurately counted as such. Despite the differences between count methods, our major findings here are independent of the method being used (fig. S10 and see below).

**Fig. 2.**
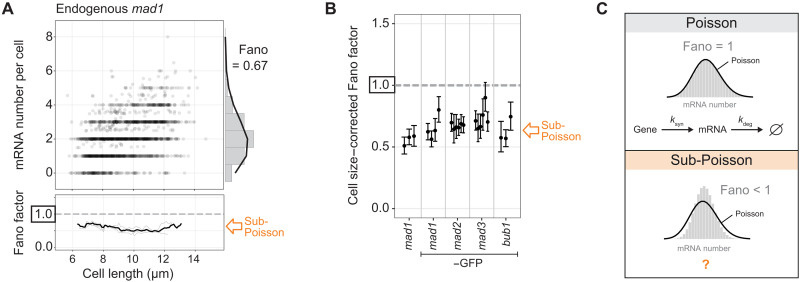
Spindle assembly checkpoint (SAC) genes show sub-Poissonian distributions of mRNA numbers. (**A**) Scatter plot of cell length versus mRNA number per cell for untagged *mad1* (*n* = 1619 cells; data from three replicates combined). Right: Histogram of mRNA number across all cells with fit to a Poisson distribution (black line). Bottom: The Fano factor was determined in a sliding window spanning 1 μm of cell length. The Fano factors for single replicates are shown in light gray; the Fano factor for the pooled data is shown in black. See fig. S11 for other SAC genes. (**B**) Cell size–corrected Fano factors and their 95% confidence interval were calculated from single-molecule mRNA fluorescence in situ hybridization (smFISH) data as in (*46*). At least three replicate experiments for each gene; the number of cells in single replicates ranged from 209 to 778. (**C**) Simulations of a Poissonian and sub-Poissonian distribution. Constitutive gene expression with a single rate-limiting step in transcription and mRNA degradation yields a Poisson distribution of mRNA numbers per cell (Fano factor = 1). What creates a narrower than Poissonian distribution (Fano < 1) for the SAC genes is unclear.

The cell-to-cell variation in mRNA numbers is a result of intrinsic and extrinsic noise sources (*1*, *2*). A considerable extrinsic influence is cell size. Mean mRNA numbers increase with cell size (see Fig. 2A and fig. S11A for examples) so that mRNA concentrations (number divided by cell volume) remain approximately constant as cells grow (*23*, *25*, *46*–*48*). To exclude the influence of size, we either considered only cells within a given size range (Fig. 2A and fig. S11A) or mathematically corrected the variance for the size effect (Fig. 2B and fig. S11B; see Materials and Methods) (*46*). When excluding the influence of cell size, all four SAC genes (*mad1*, *mad2*, *mad3*, and *bub1*) showed Fano factors of their mRNA distributions that were clearly below 1, with *mad1* reaching as low as 0.5 (Fig. 2, A and B; fig. S11, A and B; and Supplementary Text A.2). This was independent of whether we used cell length or cell volume as the size parameter (fig. S11C) and, for *mad1*, was the case regardless of whether a green fluorescent protein (GFP) tag was present or not (Fig. 2, A and B; fig. S11, A and B; and Supplementary Text A.2). Further statistical analysis showed that these Fano factors below 1 could not be explained by finite sample size effects (Supplementary Text B.7). A Poisson distribution (Fano factor = 1; Fig. 2C) would have been expected if SAC genes were expressed with a single rate-limiting step, as is typically assumed for constitutively expressed genes. Hence, this observation suggests that other, potentially more complex mechanisms account for the ultralow Fano factor of the SAC gene mRNA distributions.

**Fig. 3.**
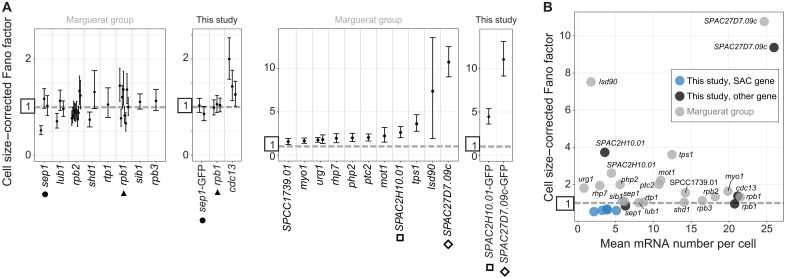
Similar results for non–spindle assembly checkpoint (SAC) genes between previously published data and our analysis. (**A**) Cell size–corrected Fano factors and their 95% confidence interval were calculated from single-molecule mRNA fluorescence in situ hybridization (smFISH) data as described in (*46*), either using published smFISH data by Marguerat and colleagues (*8*, *23*) or our own data. Genes analyzed both by Marguerat and colleagues (*8*, *23*) and this study are highlighted by icons. Between 1 and 10 replicate experiments per gene for Marguerat and colleagues’ data (*8*, *23*) (between 103 and 441 cells in single replicates) and between one and three replicate experiments per gene for this study (between 236 and 676 cells in single replicates). (**B**) Cell size–corrected Fano factors for individual genes plotted against the mean mRNA number per cell. Data from replicates were pooled. Between 236 and 3501 cells per gene for our data and between 103 and 2564 cells per gene for Marguerat and colleagues’ data (*8*, *23*). For our data, the spot count for mRNA number was used for better comparability to Marguerat and colleagues’ data (*8*, *23*).

The distribution of mRNA numbers in *S. pombe* for several other genes had previously been examined, and some genes showed mRNA distributions close to a Poisson distribution (*8*, *23*). When we corrected these published data for cell size in the same way we had done for the SAC genes, five genes (*sep1*, *lub1*, *rpb2*, *shd1*, and *rpb1*) showed single experiments with evidence for sub-Poissonian mRNA distributions, but none of them as consistently across multiple experimental replicates as we observed for the SAC genes (Fig. 3A). When pooling cells from all experimental replicates, none of these genes had a Fano factor significantly below 1 (fig. S2). We retested two of these genes (*sep1* and *rpb1*), as well as two higher noise genes (*SPAC2H10.01* and *SPAC27D7.09c*), with our workflow and found the mRNA numbers and variance to be highly consistent with those obtained previously, cross-validating the data (Fig. 3A and fig. S11D). We did not observe a correlation between mean mRNA numbers and cell size–corrected Fano factors across these genes (Fig. 3B). SAC genes show low mean mRNA numbers (means of 2 to 5). Other genes with similarly low mean mRNA numbers can show considerably larger Fano factors. In summary, the SAC genes show the lowest Fano factor in mRNA number distributions among all *S. pombe* genes for which we are aware that cell size and mRNA number have been reported.

### SAC genes have short promoters, variable 5′UTR lengths, and typical 3′UTR lengths

The sub-Poissonian mRNA number distribution of SAC genes raised the question of whether these genes share any peculiar characteristics, possibly in the promoter or the mRNA. We first mapped the 5′ and 3′ untranslated regions (UTRs) because they may influence mRNA dynamics, and because the 5′UTR length provides insight into the position of the promoter. The 5′UTRs of *mad1* and *mad2* are extremely short [~10 nucleotides (nt)], below the sixth percentile of all *S. pombe* genes (fig. S12, A and B). The 5′UTR of *mad3* (~270 nt) was longer than the interquartile range observed in *S. pombe*, whereas that of *bub1* (~60 nt) was within the interquartile range. Our results were highly consistent with a recent genome-wide study identifying transcription start sites (TSSs) in *S. pombe* (fig. S12C) (*49*). The very short 5′UTRs were unexpected since short 5′UTRs have been shown to impair translation from the first start codon in the mRNA (*50*). The only gene ontologies that were enriched for genes with such short 5′UTRs were those of ribosomal proteins (fig. S12D). Although the short 5′UTRs of *mad1* and *mad2* are unusual, the strong differences in 5′UTR length among the SAC genes imply that there is no direct correlation with the sub-Poissonian mRNA distributions.

**Fig. 4.**
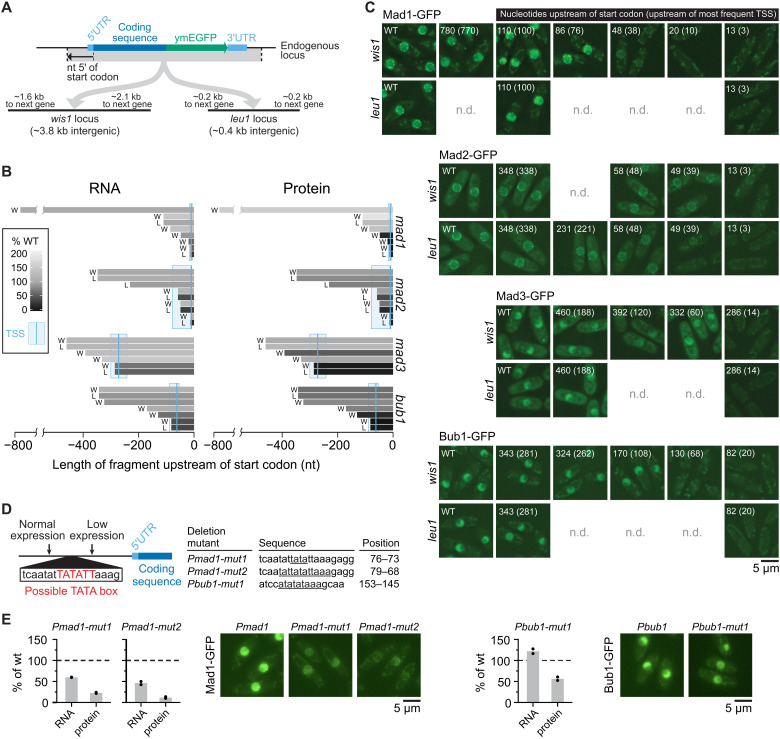
Spindle assembly checkpoint (SAC) genes have short promoter regions. (**A**) Diagram showing the experimental design of integrating SAC genes at two exogenous, intergenic loci. (**B**) Amount of RNA [by quantitative polymerase chain reaction (qPCR)] and protein (by immunoblot quantification; see fig. S13) from exogenous locus expression (W = *wis1* locus, L = *leu1* locus) relative to expression from the endogenous locus. The length of the bars shows the length of the upstream region (promoter and 5′UTR) and shades of gray show the mRNA or protein amount; qPCR primers and immunoblot antibody targeted the green fluorescent protein (GFP) tag. Data from one experiment are shown. (**C**) Representative live-cell images from strains expressing GFP-tagged SAC genes from the endogenous locus (WT, wild type) or with differently sized upstream regions from an exogenous locus (*wis1* or *leu1*). Numbers indicate the size of the upstream region in nucleotides either measured from the start codon or from the most prominent transcription start site (TSS) (in brackets). Gamma correction for images to make both high and low expression cells visible: Mad1 = 2.0, Mad2 = 1.8, Mad3 = 1.0, and Bub1 = 1.5. (**D**) Schematic (left) and table (right) of deletions to test the functionality of possible TATA box sequences. Deleted bases are underlined in the table and their positions are given in base pairs upstream of the start codon. (**E**) Relative expression of RNA (by qPCR) and protein (by immunoblot quantification; see fig. S13) from deletion mutants in (D) normalized to strains with wild-type promoter sequences (two biological replicates each). One-sample *t* tests: *Pmad1-mut1* RNA *P* = 0.007, *Pmad1-mut1* protein *P* = 0.01, *Pmad1-mut2* RNA *P* = 0.04, *Pmad1-mut2* protein *P* = 0.01, *Pbub1-mut1* RNA *P* = 0.16, and *Pbub1-mut1* protein *P* = 0.06. Representative live-cell images of the GFP-tagged protein expressed from the wild-type promoter or the TATA box mutants at the exogenous *leu1* locus (gamma correction: 1.5).

Promoters have a large influence on gene expression noise (*51*–*55*). The location, size, and characteristics of most promoters in *S. pombe* remain poorly defined (*49*, *56*). To map the promoter regions of the SAC genes, we transferred these genes with a variable length of sequence upstream of the TSS (3 to 770 nt) to two different intergenic loci: (i) the 3.8 kb region next to *wis1*, one of the largest intergenic regions in *S. pombe*, and (ii) the 0.4 kb region next to *leu1*, which is a frequently used integration site in *S. pombe* (Fig. 4A) (*57*). We used two loci to be able to control for effects from the surrounding regions. We found that expression only started to break down when less than 50 to 100 base pairs of the upstream sequence was retained (Fig. 4, B and C, and figs. S13A and S14). Monitoring mRNA levels by qPCR and protein levels by either immunoblotting or imaging gave similar results (Fig. 4, B and C). This suggests that the expression breakdown reflects the inability of the coding sequence to be transcribed. The results from the two intergenic loci were overall consistent, with some quantitative differences observed for *mad2* (Fig. 4, B and C, and fig. S13A).

On the basis of the short length of the candidate promoter regions, expression is likely driven by only a core promoter. Consistently, *mad1* and *bub1* had TATA box-like sequences in a stretch of upstream sequence that, when removed, markedly lowered expression (fig. S14). This was unexpected because TATA boxes are typically found in promoters causing large noise (*12*, *51*, *52*, *54*). Mutating the candidate TATA box in *mad1* clearly lowered expression (Fig. 4, D and E, and fig. S13B), consistent with this sequence acting as a TATA box. Mutating the candidate TATA box in *bub1* did not reduce the RNA concentration (Fig. 4, D and E), but *bub1* contains another TATA box-like sequence upstream (fig. S14), which may have compensated. We conclude that the expression of SAC genes is likely driven by core promoter sequences only.

### Regulatory SAC gene sequences are sufficient for mRNA distributions in the Poisson-range, but not for sub-Poissonian mRNA distributions

**Fig. 5.**
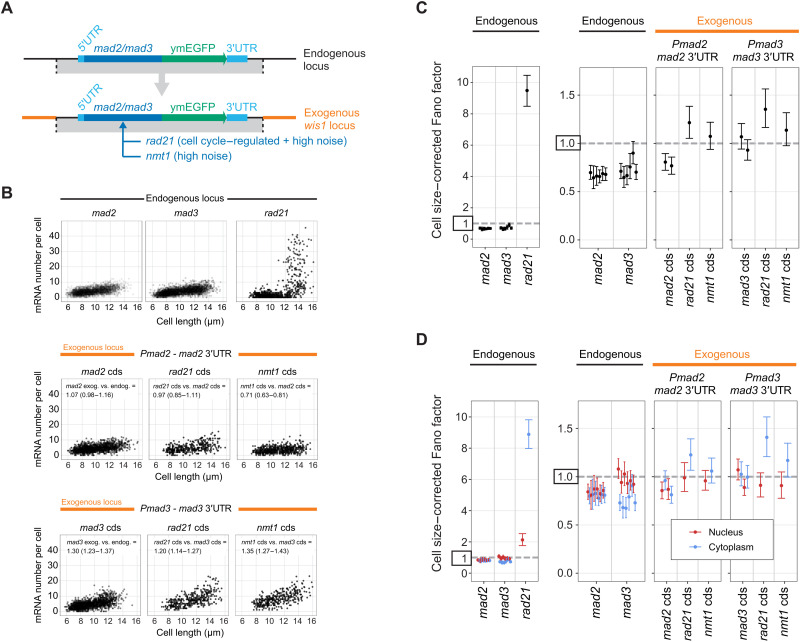
Small surrounding regions are sufficient for low-noise expression, but not for sub-Poissonian mRNA distributions. (**A**) *mad2*–green fluorescent protein (GFP) or *mad3*-GFP with their surrounding genomic region was integrated at the intergenic locus adjacent to the *wis1* gene. For *mad2*, 48 nt upstream of the most prominent transcription start site (TSS) to 521 nt downstream of the stop codon were used; for *mad3*, 60 nt upstream of the most prominent TSS to 279 nt downstream of the stop codon were used. In additional strains, the coding sequences (cds) of *mad2* or *mad3* were replaced with those of *rad21* or *nmt1*. (**B**) Top: mRNA number relative to cell length at the endogenous locus for each gene (*mad2*: 3501 cells from six independent experiments; *mad3*: 2993 cells, six independent experiments; *rad21*: 678 cells, one experiment; data for *mad2* and *mad3* are the same as in Fig. 2). Middle and bottom: Same for expression from the *mad2* promoter (*Pmad2*) and *mad2* downstream region or *mad3* promoter (*Pmad3*) and *mad3* downstream region at the *wis1* locus (*Pmad2-mad2*: 1308 cells from two independent experiments; *Pmad2-rad21*: 375 cells, one experiment; *Pmad2-nmt1*: 557 cells, one experiment; *Pmad3-mad3*: 1586 cells, two independent experiments; *Pmad3-rad21*: 368 cells, one experiment; *Pmad3-nmt1*: 342 cells, one experiment). Generalized linear mixed model estimates for the ratio in mRNA number per cell between conditions with a 95% confidence interval in brackets are given; also see fig. S15C. (**C**) Cell size–corrected Fano factors and their 95% confidence interval were calculated from single-molecule mRNA fluorescence in situ hybridization (smFISH) data as in Fig. 2 using the data shown in (B). Between one and six experiments for each genotype; the number of cells in single experiments ranged from 209 to 794. (**D**) As in (C), but the cell size–corrected Fano factor was calculated separately for mRNA localizing to the nucleus or the cytoplasm.

To test whether the identified promoters are sufficient for expression with low Fano factors, we placed the coding sequences of two high-noise genes under the *mad2* and *mad3* promoters at the intergenic locus next to the *wis1* gene (Fig. 5A). We used *rad21*, which is a cell cycle–regulated gene whose Fano factor can increase to around 12 during expression late in the cell cycle (Fig. 5B and fig. S15, A and B) (*58*), and *nmt1*, which is a thiamine-responsive, highly variable gene (*8*, *39*). When expressing *rad21* and *nmt1* from the *mad2* and *mad3* promoters, the cell cycle regulation of *rad21* disappeared (Fig. 5B and fig. S15), and the Fano factor of the mRNA distributions of *rad21* and *nmt1* was lowered to a value slightly above 1 (Fig. 5C). This was close to, but not quite as low, as the *mad2* and *mad3* coding sequences expressed from their own promoter at the exogenous locus (Fig. 5C). Overall, this confirms that promoter sequences have a large influence but suggests that a combination of gene elements is required for the very narrow mRNA distributions observed for SAC genes.

To explore the subtle differences in noise between endogenous and exogenous loci, and between the endogenous and exogenous coding sequences, we determined the size-corrected Fano factor separately for the nucleus, where mRNAs are produced, and for the cytoplasm, where mRNAs are ultimately translated. We assigned mRNAs to either the nucleus or the cytoplasm after segmenting nuclei in three dimensions (3D) based on DNA staining and correcting for chromatic aberration (fig. S16A). This revealed interesting differences. For *mad3* at its endogenous locus, the Fano factor of the mRNA distribution was lower in the cytoplasm than in the nucleus, whereas it was similar in the cytoplasm and nucleus for *mad2* (Fig. 5D). When *mad3* was expressed from the exogenous locus, though, its Fano factor also became similar between the nucleus and cytoplasm. Furthermore, *rad21* and *nmt1* expressed from the same regulatory sequences tended to show a higher Fano factor in the cytoplasm than in the nucleus (Fig. 5D). Together, this suggested that an interplay between promoter, coding sequence, and surrounding sequences may be necessary for sub-Poissonian mRNA distributions, and that the sub-Poissonian mRNA expression of SAC genes is maintained or further suppressed in the cytoplasm.

### Most SAC genes show lower mRNA Fano factors in the cytoplasm than in the nucleus

**Fig. 6.**
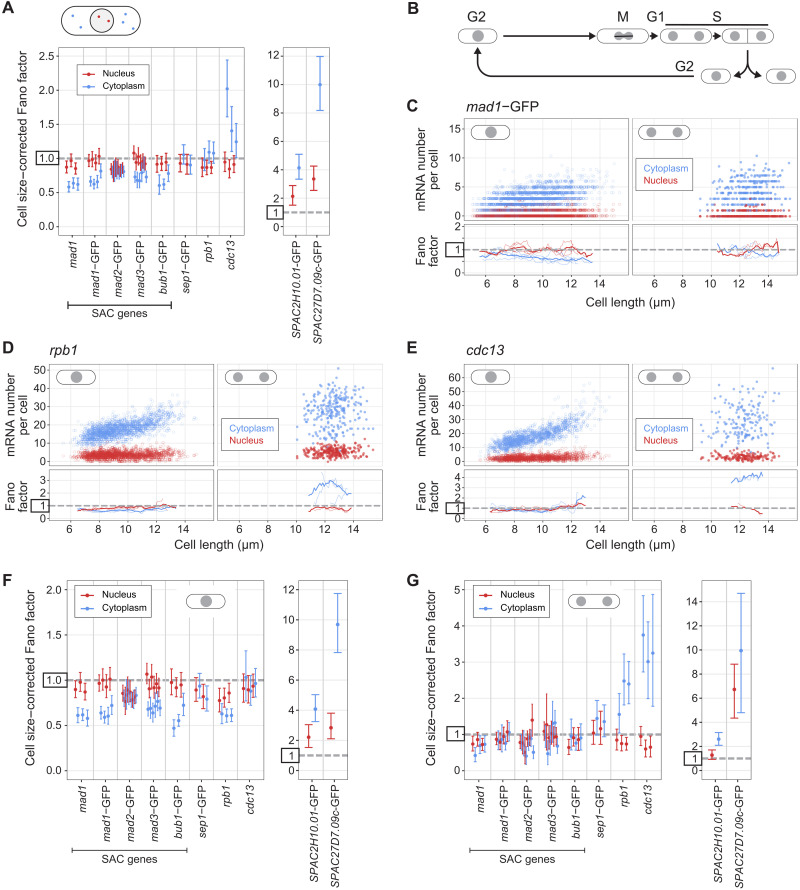
The Fano factor of the mRNA distribution can be lower in the cytoplasm than in the nucleus. (**A**) Cell size–corrected Fano factors and their 95% confidence interval were calculated from single-molecule mRNA fluorescence in situ hybridization (smFISH) data. The same experiments as in Figs. 2 and 3, but nuclei were segmented using 4′,6-diamidino-2-phenylindole (DAPI) staining, and the cell size–corrected Fano factor was calculated separately for mRNA localizing to the nucleus and localizing to the cytoplasm. Between one and six replicate experiments for each genotype; the number of cells in single replicates ranged from 209 to 778. (**B**) Schematic of the *S. pombe* cell cycle. (**C** to **E**) Top: Scatter plot of cell length versus mRNA number in the cytoplasm (blue) or nucleus (red). Mono- and binucleated cells are shown separately. Bottom: The Fano factor was determined in a sliding window spanning 1 μm of cell length. The Fano factors for single replicates are shown as thin lines; the Fano factor for the pooled data is shown as a thick line. Same experiments as in (A). See fig. S17 for other spindle assembly checkpoint (SAC) genes. (**F** and **G**) As in (A), but analyzed separately for mononucleated (F) and binucleated (G) cells.

To test for the generality of this observation, we determined size-corrected nuclear and cytoplasmic Fano factors for all SAC genes and the other genes examined previously, all expressed from their endogenous locus (Fig. 6A and Supplementary Text A.3). Notably, all SAC genes, with the exception of *mad2*, showed lower Fano factors in the cytoplasm than in the nucleus (Fig. 6A and Supplementary Text A.3). Other low-noise genes (*sep1* and *rpb1*) showed a similar or slightly higher Fano factor in the cytoplasm than in the nucleus, and high-noise genes (*SPAC2H10.01* and *SPAC27D7.09c*) showed a considerably higher Fano factor in the cytoplasm than in the nucleus. These results suggest that, for most SAC genes, the Fano factor of their mRNA distribution is further lowered posttranscriptionally.

These data with segmented nuclei also allowed us to distinguish mononucleated and binucleated cells (Fig. 6, B to G). In *S. pombe*, the nuclear envelope does not break down during cell division, and chromosome segregation is followed by the splitting of the nucleus into two and, ultimately, cell division (*59*). The G1 phase in *S. pombe* is short and DNA replication occurs mostly before cell division, i.e. during the binucleated stage. Thus, binucleated cells are in late mitosis, G1, or S phase, whereas almost all mononucleated cells are in G2 (Fig. 6B). We observed significant Fano factor differences between mononucleated and binucleated cells for almost all genes, with stronger differences in the cytoplasm than the nucleus for the low-noise genes (Fig. 6, C to G, fig. S17, and Supplementary Text A.4). The Fano factor suppression between the nucleus and cytoplasm for SAC genes went away during the binucleated stage, and *rpb1* and *cdc13* showed drastic increases in cytoplasmic (but not nuclear) Fano factor in binucleated cells (Fig. 6, F and G). Overall, this most likely reflects marked changes in cellular physiology during cell division and DNA replication, which could affect mRNA synthesis, nuclear export, mRNA degradation, or possibly all of those (*60*–*63*). It also illustrates that it is important to minimize cell cycle effects when analyzing gene expression noise and its mechanistic basis (*25*, *64*).

### The Fano factor of the *mad1* mRNA distribution is lower in the cytoplasm than at the transcription site or in the nucleoplasm

**Fig. 7.**
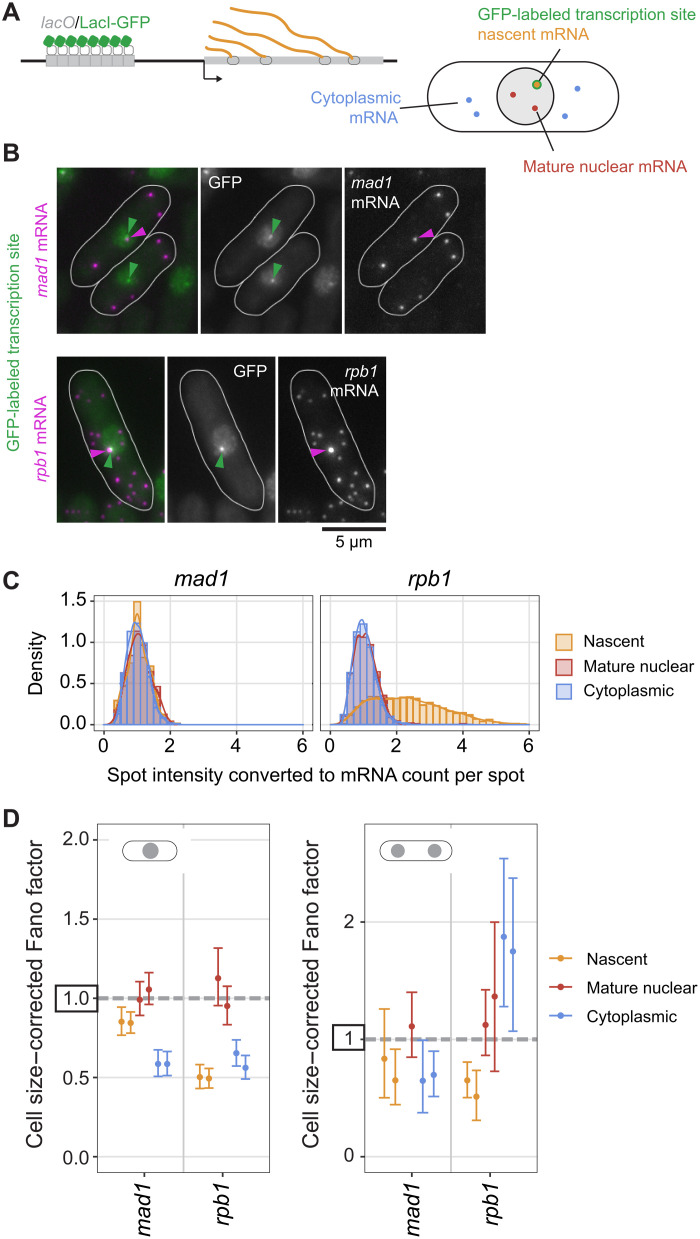
Transcription site labeling confirms a larger Fano factor of mature nuclear than cytoplasmic mRNA. (**A**) Diagram of transcription site (TS) labeling and classifying mRNA as nascent [associated with the green fluorescent protein (GFP)–labeled gene], mature nuclear, or cytoplasmic. (**B**) Representative images of *mad1* or *rpb1* mRNA fluorescence in situ hybridization (FISH) in cells with the TS labeled by GFP. (**C**) Histogram and density distribution of FISH spot intensity at the TS (nascent), at other positions in the nucleus (mature nuclear), or in the cytoplasm. Data were normalized to the median of the spot intensity in the cytoplasm for each image. Pooled data from two independent experiments; *n* = 2217 spots for *mad1*; *n* = 20,740 spots for *rpb1*. See fig. S18C for the independent experiments. (**D**) Cell size–corrected Fano factors and their 95% confidence interval were calculated from single-molecule mRNA FISH (smFISH) data for nascent, mature nuclear, or cytoplasmic mRNA. Mono- and binucleated cells were analyzed separately. Two replicate experiments for each genotype; one of the experiments did not have enough binucleated cells with mature nuclear *mad1* mRNA for a reliable estimate of the cell size–corrected Fano factor in this compartment. The number of cells in single replicates ranged from 343 to 541 for mononucleated and 32 to 98 for binucleated cells.

Our observations suggested that the Fano factor of SAC gene mRNA distributions is further lowered posttranscriptionally. To strengthen this conclusion, we additionally examined mRNA directly at the TS (Fig. 7). Using a strategy similar to one previously used in *Escherichia coli* (*65*), we labeled the TS by integrating lac operator (lacO) repeats into an intergenic region 3.3 and 6.4 kb away from *mad1* and *rpb1*, respectively, and expressing a Lac inhibitor–GFP fusion protein with a nuclear localization signal in these cells (Fig. 7A). This resulted in typically one GFP spot in the nucleus (Fig. 7B). Both mean mRNA numbers and cell size–corrected Fano factors for *mad1* and *rpb1* remained highly similar to cells without the TS labeled (fig. S18A), suggesting that the integration did not alter expression from the endogenous locus.

We classified mRNAs in the direct vicinity of the GFP spot as nascent (Fig. 7A and fig. S16B). Occasionally, a TS was just outside of the region that we had classified as nuclear based on DNA labeling and segmentation. In such cases, we isotropically expanded the nucleus to include the TS and adjusted our classification of nuclear and cytoplasmic mRNA accordingly (fig. S16B). Excluding these cells from the analysis as an alternative method yielded similar results (compare Fig. 7D and fig. S18B). For the highly expressed *rpb1* gene, this procedure captured all high-intensity mRNA spots as nascent (Fig. 7, B and C, and fig. S18C), confirming the validity of the approach. This procedure then allowed us to distinguish nascent, mature nuclear, and cytoplasmic mRNAs (Fig. 7A). For both *mad1* and *rpb1*, the number of mature nuclear mRNAs was lower than the number at the TS, indicating fast nuclear mRNA export (fig. S18D), as has been previously found for budding yeast (*66*). Consequently, the number of all nuclear mRNAs is not a good proxy for the number of mature nuclear RNAs (fig. S18D and Supplementary Text B.5).

The analysis of the cell size–corrected Fano factors in the different compartments showed that both *mad1* and *rpb1* are transcribed in a way that yields a sub-Poissonian distribution of mRNA numbers at the TS, even stronger for *rpb1* than for *mad1* (Fig. 7D, figs. S18E and S19, and Supplementary Text A.2). The Fano factor of mature nuclear mRNA was close to 1. In agreement with our previous observations (Fig. 6F), the Fano factor of cytoplasmic *mad1* mRNA in mononucleated cells was clearly lower than either in the nucleoplasm or at the TS (Fig. 7D and Supplementary Text A.3). As we had seen before, suppression of the Fano factor in the cytoplasm largely disappeared in binucleated cells (Fig. 7D). The results were independent of whether we used cell length or cell volume as the size parameter (fig. S18F). Furthermore, the same trends were observed when binning cells by their size and analyzing the Fano factor of the mRNA distribution in cells of similar size (fig. S18G). Overall, these results show that both these constitutively expressed genes show characteristics of transcription that lead to a sub-Poissonian distribution of mRNA numbers at the TS. In addition, the results confirm that the Fano factor of the cytoplasmic mRNA distribution can be significantly lower than that in the nucleoplasm, or even at the TS.

### Multiple rate-limiting steps in transcription and degradation, combined with fast nuclear export, can explain the sub-Poissonian mRNA distributions

How can the observed sub-Poissonian mRNA distributions be explained mechanistically? The classical, widely used model for constitutive gene expression assumes single rate-limiting steps for mRNA synthesis and degradation with exponential wait times between single synthesis or degradation events (*16*–*18*). Under these assumptions, the Fano factor of mature mRNA in both the nucleus and cytoplasm will be 1 (*20*). This may be consistent with data for some constitutively expressed genes, but clearly not all.

**Fig. 8.**
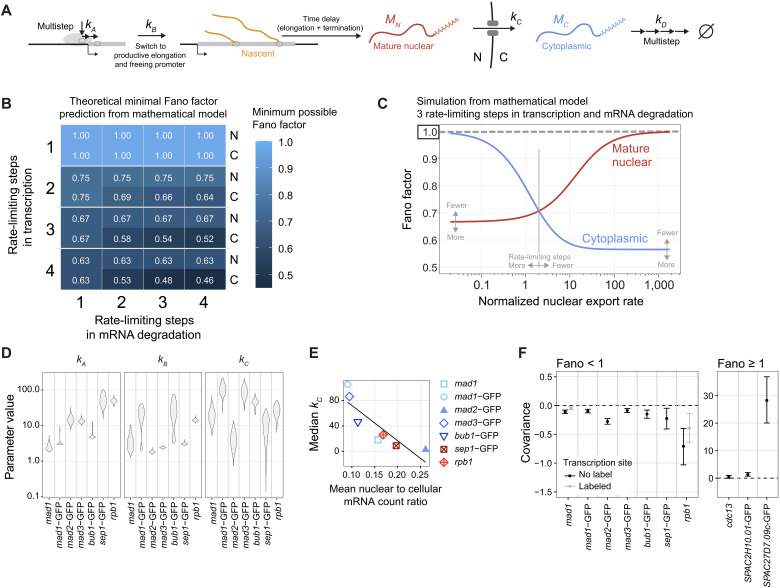
A sub-Poissonian mRNA distribution in the cytoplasm can be explained by multiple rate-limiting steps in mRNA degradation and is modulated by nuclear export kinetics. (**A**) Outline of the parameters in the mathematical model, including multiple rate-limiting steps in mRNA synthesis and degradation. (**B**) Analytical results from the mathematical model: minimum possible Fano factors in the nucleus (N) and cytoplasm (C) depending on the number of rate-limiting steps in transcription and mRNA degradation. (**C**) Expectation for nuclear and cytoplasmic Fano factors for a model with three rate-limiting steps in both transcription and mRNA degradation; *k_A_* = *k_B_* = 10; *k_D_* = 3. The *x* axis shows the nuclear export rate *k_C_*, which is defined relative to the effective mRNA degradation rate. The nuclear export rate at which the Fano factors are equal scales inversely with the number of rate-limiting steps in transcription and mRNA degradation (table S7). Gray arrows illustrate in which direction the curves are expected to change for fewer or more rate-limiting steps in transcription and mRNA degradation. (**D**) Posterior distributions of model parameters obtained using an approximate Bayesian computation (ABC) rejection sampler from fitting the model *S* = *R* = 3 to data from the sub-Poisson genes in mononucleated cells. (**E**) Relationship between median *k_C_* from (D) and mean nuclear to cellular mRNA count ratio; *n* = 586 to 3064 cells per gene. Linear regression fit: *P* = 0.012, adj. *R*^2^ = 0.70. (**F**) Covariance between nuclear and cytoplasmic [no transcription site (TS) label] or mature nuclear and cytoplasmic (TS-labeled) mRNA counts in mononucleated cells. The means and 95% confidence interval were calculated. Same experiments as in Figs. 6F and 7D, but cells from replicate experiments were pooled for each gene.

To obtain a distribution of mRNA numbers that is narrower than the Poisson distribution, the wait times between single mRNA synthesis events need to be more homogeneous than in an exponential distribution (*21*). Assuming multiple steps in mRNA synthesis with similar timescales, rather than just one rate-limiting step, will lead to such narrower than exponential wait times (*21*, *30*, *33*, *37*). Hence, we constructed a model (Fig. 8A and Supplementary Text B) whose effective reaction scheme (excluding nascent RNA dynamics) is

$$U_{0}⇌U_{1}\to U_{2}\to \cdots \to U_{S}\to U_{1}+M_{N},M_{N}\to M_{C}^{1}\to M_{C}^{2}\to \cdots \to M_{C}^{R}\to \emptyset$$

The promoter state *U*_0_ denotes the state in which chromatin is closed. The promoter state *U*_1_ denotes the state in which chromatin is open but no RNA polymerase II (Pol II) is bound to the promoter. Promoter binding, opening, escape, and other downstream events at the promoter are represented by states (*U*_2_ to *U*_*S*−1_). The last state *U_S_* is a promoter-proximal paused state; release from this state simultaneously leads to the clearing of the promoter for new Pol II binding (and hence return to state *U*_1_) and the beginning of the elongation of the nascent transcript which results in the production of a mature mRNA, *M_N_* (*67*–*69*). The latter is then exported from the nucleus to the cytoplasm where degradation of the mRNA occurs. Degradation of mRNA is also known to involve multiple steps (*70*), and it has been argued that assuming several rate-limiting steps is necessary to fit the majority of transcriptome-wide mRNA decay data from budding yeast (*71*). Hence, the model assumes *R* rate-limiting steps in mRNA degradation, and the cytoplasmic mRNA in step *i* is denoted by $M_{C}^{i}$.

We note two special cases of the model that are well-studied in the literature (Supplementary Text B). In the first special case, the classical model for constitutive gene expression, chromatin is always open and the intermediate promoter states are short-lived, i.e., there is a single rate-limiting step for mRNA synthesis, which leads to the simplified model

$$U_{1}\to U_{1}+M_{N},M_{N}\to M_{C}^{1}\to M_{C}^{2}\to \cdots \to M_{C}^{R}\to \emptyset$$

in which case the Fano factor of the cytoplasmic mRNA distribution will be equal to 1. In the second special case of the model, chromatin switches between open and closed states, while intermediate promoter states are also short-lived, which leads to the well-known two-state telegraph model of gene expression

$$U_{0}⇌U_{1}\to U_{1}+M_{N},M_{N}\to M_{C}^{1}\to M_{C}^{2}\to \cdots \to M_{C}^{R}\to \emptyset$$

where the Fano factors are greater than 1.

If we assume constantly open chromatin (no *U*_0_ state), without assuming a single rate-limiting step for mRNA synthesis (i.e., long-lived intermediate promoter states), we obtain the model

$$U_{1}\to U_{2}\to \cdots \to U_{S}\to U_{1}+M_{N},M_{N}\to M_{C}^{1}\to M_{C}^{2}\to \cdots \to M_{C}^{R}\to \emptyset$$

which can predict Fano factors less than 1 and thus explain sub-Poissonian noise (Fig. 8, A and B).

We derived mathematical expressions for the Fano factor in steady-state conditions to determine the minimal Fano factors possible for this model (Fig. 8B and Supplementary Text B). Increasing the number of transcription initiation steps decreases the possible Fano factor and allows for Fano factors below 1 (Fig. 8B). Increasing the number of rate-limiting steps in mRNA degradation allows for lower Fano factors in the cytoplasm than in the nucleus (Fig. 8B), in agreement with what we observed experimentally (Figs. 6 and 7). Whether the cytoplasmic Fano factor is lower or higher than the nuclear Fano factor depends on the nuclear export rate in this model (Fig. 8C). At slow nuclear export rates, the Fano factor in the cytoplasm is larger than that in the nucleus. Only at fast nuclear export rates will the cytoplasmic Fano factor become smaller than that in the nucleus. The threshold value at which the switch occurs becomes lower with an increasing number of rate-limiting steps in transcription and mRNA degradation (Fig. 8C and table S7). Hence, our mathematical model shows that multiple rate-limiting steps in transcription and mRNA degradation, along with efficient nuclear export of mRNA, can explain both the sub-Poissonian mRNA distributions and the lower Fano factor in the cytoplasm than in the nucleus. Furthermore, nuclear export can be an efficient lever to tune cytoplasmic mRNA variability in this sub-Poisson regime.

### At least three rate-limiting steps in synthesis and mRNA degradation are required to explain all SAC gene data

To determine how many rate-limiting steps in transcription and mRNA degradation are required to explain the experimental data, we used Bayesian model selection. We found strong evidence that three or four rate-limiting steps in both transcription and mRNA degradation are required to explain the data for *mad1*, *mad1*-GFP, and *bub1*-GFP (table S18). The expression of the other genes (*mad2*-GFP, *mad3*-GFP, *rpb1*, and *sep1*-GFP) could be equally well explained by other combinations of the number of rate-limiting steps, with the provision that there are at least two steps for transcription. For each gene, we then estimated the rate constants for promoter remodeling (*k_A_*; the rate of switching from *U_i_* → *U*_*i*+1_), promoter freeing (*k_B_*; the rate of initiating nascent mRNA production, *U_S_* → *U*_1_ + *M_N_*), and nuclear export (*k_C_*; the rate of the reaction $M_{N}\to M_{C}^{1}$) relative to the effective rate of cytoplasmic degradation (the inverse of the sum of the lifetimes of all cytoplasmic mRNA species $M_{C}^{i}$). We used a model with three rate-limiting steps in both transcription and mRNA degradation (*S* = *R* = 3 in the reaction scheme). The estimates indicated some differences between genes (Fig. 8D and table S19). The non-SAC genes, *rpb1* and *sep1*, were best fit with higher rates of promoter remodeling (*k_A_*); the two genes not showing a reduction of the Fano factor in the cytoplasm, *mad2* and *sep1*, were best fit with lower rates of nuclear export (*k_C_*). Consistent with a lower nuclear export rate, *mad2* and *sep1* have a higher fraction of their mRNA in the nucleus (Fig. 8E and fig. S17G), which is information that was not used in the fitting and therefore supports the model. Furthermore, an analytical examination of the model revealed that the covariance between nuclear and cytoplasmic mRNA counts is expected to be negative if the fluctuations are sub-Poissonian, zero if Poissonian, and positive if super-Poissonian (Supplementary Text B.3). Consistent with this expectation, we observe negative covariances for the genes with Fano factor less than 1 and positive covariances for genes with Fano factor larger than 1 (Fig. 8F).

Together, our results imply that the sub-Poissonian mRNA distributions observed for some constitutively expressed genes can be biologically explained by multiple rate-limiting steps in transcription and mRNA degradation. Furthermore, our results indicate that constitutively expressed genes are not a homogenous group, but instead differ measurably in their expression characteristics with consequences for mRNA noise.

## DISCUSSION

The expression characteristics of low-noise genes have received less attention than those of high-noise genes. Here, we showed that the mRNA distribution of eukaryotic, constitutively expressed eukaryotic genes can be narrower than a Poisson distribution, which establishes that the intrinsic noise floor of constitutively expressed genes is sub-Poissonian, not Poissonian. This knowledge is relevant because researchers have at times eliminated data from their analysis when the mRNA distributions appeared sub-Poissonian (*72*). On the basis of our results, this may have excluded valid and interesting data. Our findings imply that the widely used model for constitutive expression with single rate-limiting steps in transcription and mRNA degradation is an oversimplification. We suggest that—in their most basic, unregulated form—the multistep nature of transcription and mRNA degradation leads to sub-Poissonian, not Poissonian, noise. However, certain promoter architectures or added-on regulation, e.g., by a transcription factor or by an RNA-binding protein, can introduce additional intrinsic noise and thereby move a gene closer to Poisson-type expression. Overall, low-noise constitutive gene expression may be more varied than previously thought.

Our findings were unexpected because prior reports of sub-Poissonian mRNA distributions are extremely sparse. In bacteria, one group has reported that expression from the *tetA* promoter results in a sub-Poissonian mRNA distribution (*34*) and that the wait times between synthesis events from this and two other bacterial promoters are more narrowly distributed than exponential, consistent with two or three rate-limiting steps in transcription (*28*, *34*, *35*). Others concluded that gene expression noise in bacteria is generally Poissonian or super-Poissonian (*5*, *24*). In eukaryotes, mRNA distributions of constitutively expressed genes have generally been reported to be Poissonian (*3*, *22*, *23*). However, some of these values were obtained without correcting for cell size, which may have made a sub-Poissonian distribution appear Poissonian (see fig. S11A for an example). In addition, unrecognized cell cycle effects may have muddied the data. For example, the cytoplasmic mRNA distribution of *S. pombe rpb1* (coding for the largest subunit of Pol II) has a Fano factor below 1 in mononucleated cells (Fig. 6F), but clearly above in binucleated (Fig. 6G), which can average to around 1 across the population (Fig. 6A)—and hence, its expression was assumed Poissonian (*23*). One report provides evidence for a sub-Poissonian mRNA distribution of the orthologous *S. cerevisiae RPB1* gene (*36*). However, finite sample effects would need to be excluded for a formal conclusion.

Overall, although prior data from eukaryotes do not specifically support sub-Poissonian distributions, they do not exclude them either. Since we used one of the gold standards to assess mRNA numbers (*73*), which yielded solid evidence of sub-Poissonian distributions, and since unrecognized biological or technical variability would only inflate the measured variability, we find it hard to escape the conclusion that the expression of constitutive genes can be sub-Poissonian.

Whether mRNA distributions are sub-Poissonian or Poissonian has at least two biological implications. First, it makes a difference in noise strength. Whether this is functionally relevant, i.e., whether increasing sub-Poissonian to Poissonian expression lowers fitness, remains to be determined. Generally, noise characteristics of genes can be under natural selection (*1*, *11*). It will be interesting to determine which classes of genes show sub-Poissonian expression and to uncover the potential connections to their function. Second, the mRNA distributions provide a window into the gene expression process. A promoter transcribing with a single rate-limiting step must be fundamentally different from one exhibiting multiple rate-limiting steps. To what extent and how constitutive promoters differ from each other is still poorly understood (*74*). Distinguishing sub-Poissonian and Poissonian low-noise genes may help to classify constitutive promoters and identify their functionally important elements. It is worth noting, though, that the elements defining sub-Poissonian expression may not be neatly confined to the promoter (Fig. 5). This highlights the important role that chromatin context (*51*) and noncoding features of the coding sequence (*75*) play in gene expression. Furthermore, it remains a possibility that sub-Poissonian distributions are brought about by negative feedback, as has been examined repeatedly in models (*26*, *27*, *29*, *31*). For the genes examined here, we are not aware of any known process establishing negative feedback, and we therefore favor multiple steps in transcription and mRNA degradation as the origin of their sub-Poissonian mRNA distributions. Another possible mechanism leading to sub-Poissonian distributions is steric hindrances between RNA Pol II molecules (*76*); however, this is an unlikely explanation for the SAC genes because they are transcribed infrequently (Fig. 7 and fig. S9).

Another relevant observation is that most SAC genes that show sub-Poissonian mRNA distributions also show a reduction of the Fano factor between the nucleus and cytoplasm. Such a reduction of variance has been seen for bursty genes, where it was attributed to slow nuclear export (*7*, *14*). In contrast, our model predicts that the nuclear export rate needs to be sufficiently fast for the Fano factor in the cytoplasm to drop below the one in the nucleus (Fig. 8C). Our analysis also shows that the cytoplasmic Fano factor can become lower with an increasing number of rate-limiting steps in mRNA degradation (Fig. 8, B and C). This seems at odds with published results that multiple rate-limiting steps in mRNA degradation can increase the Fano factor in the cytoplasm (*15*). The difference between these published results and ours is the underlying type of expression. Lowering of the cytoplasmic Fano factor by slow nuclear export or increase by multiple rate-limiting steps in mRNA degradation holds true for super-Poissonian expression (nuclear Fano factor larger than 1), but the relationships change in sub-Poissonian expression (nuclear Fano factor smaller than 1; shown analytically in Supplementary Text B.4). Hence, the sub-Poissonian regime is qualitatively distinct, but its distinct rules can be explained through a similar type of underlying gene expression model. In the future, it will be important to experimentally confirm the distinct rules in the sub-Poissonian regime.

Last, among the SAC genes we have studied, *mad2* is an interesting exception. It does show a sub-Poissonian mRNA distribution, but it does not show the Fano factor reduction between the nucleus and cytoplasm seen for the other SAC genes (Fig. 6 and fig. S17). This suggests that its nuclear export or mRNA degradation characteristics differ qualitatively from that of the other SAC genes. We speculate that this could be a consequence of the cotranslational assembly of the Mad1/Mad2 complex. The Mad1 and Mad2 proteins form an extremely tight 2:2 complex (*40*). The Mad1 dimer at the core of this complex assembles cotranslationally (*39*, *77*). We consider it possible that the binding of Mad2 to the Mad1 dimer also needs to be cotranslational. In this case, *mad2* mRNA may associate with Mad1 protein, which, in turn, may affect its export characteristics or the number of rate-limiting steps in its degradation.

In summary, we have established that the low-noise regime of eukaryotic gene expression reaches lower than previously appreciated. This opens avenues to understand the underlying molecular mechanisms and identify gene elements that minimize noise.

## MATERIALS AND METHODS

### Key materials

*S. pombe* strains are shown in table S22, single-guide RNA (sgRNA) sequences in table S23, FISH probes in table S24, and qPCR primers in table S25.

### Strain construction

SAC genes *mad1*, *mad2*, *mad3*, and *bub1* were tagged with yeast codon-optimized, monomeric enhanced GFP (ymEGFP) at the endogenous locus without inserting any other exogenous sequences (*39*). This was accomplished either by replacement of the counter-selectable *rpl42-hphNT1* cassette in an *rpl42*::*cyh^R^*(sP56Q) strain (*78*) or by using CRISPR-mediated targeting (*79*). The *mad2*-ymEGFP strain contains a single, silent (AGG to AGA) protospacer adjacent motif (PAM) site mutation at amino acid position 173 of Mad2. The *mad3*-ymEGFP strain contains a single, silent (TTG to TTA) PAM site mutation at amino acid position 199 of Mad3. ymEGFP was derived from yEGFP (*80*) by mutation of alanine-206 to arginine (A206R), which is expected to reduce dimerization (*81*). Tagging of *sep1*, *SPAC2H10.01*, *SPAC27D7.09c*, and *rad21* with ymEGFP was performed by conventional PCR-based gene targeting (*82*). Integration at the exogenous *wis1* and *leu1* loci used CRISPR-mediated targeting; sgRNA sequences are listed in table S23.

For TS labeling, lacO repeats were integrated as described by Rohner and colleagues (*83*). Insertion close to *mad1* is between the adjacent *trz2* and *but2* genes (3.3 kb away from the *mad1* stop codon), and insertion close to *rpb1* is between the adjacent *toa1* and *hrd3* genes (6.4 kb away from the *rpb1* start codon). We tried to be minimally disruptive to gene expression by choosing relatively large intergenic regions, not directly adjacent but sufficiently close to the genes of interest. A resistance cassette (*hphMX4* from pAG32) was inserted at the respective locus, and then swapped for the lacO repeats and *LEU2* as a resistance marker (from pSR13). The swap was facilitated by CRISPR-Cas9 targeting of the resistance cassette.

### Cell growth

Yeast extract with adenine [YEA; glucose (30 g/liter), yeast extract (5 g/liter), adenine hemisulfate dihydrate (0.15 g/liter)] was used as a rich medium; Edinburgh minimal medium (EMM; MP Biomedicals, 4110032) was used as a minimal medium. Strains were thawed on YEA plates, and then grown for approximately 24 hours either in liquid YEA or in liquid EMM, all at 30°C. When cultures were diluted to low densities, 50% preconditioned medium (made by filtering EMM cultures) was added to EMM. l-Leucine (0.2 g/liter) was added to the EMM medium as needed for auxotrophic strains.

### Single-molecule mRNA fluorescence in situ hybridization

Cultures were grown to ~0.7 × 10^7^ to 1.5 × 10^7^ cells/ml and 2 × 10^8^ cells were fixed with either 2% or 4% formaldehyde. After 30 min, formaldehyde was washed out with three washes of ice-cold buffer B [1.2 M sorbitol and 100 mM potassium phosphate buffer (pH 7.5)]. When not immediately continuing with the next step, cells were stored in 1 ml of buffer B at 4°C. Fixed cells were resuspended in 1 ml of spheroplast buffer [1.2 M sorbitol, 0.1 M potassium phosphate, 20 mM vanadyl ribonuclease complex (NEB, S1402S), and 20 μM beta-mercaptoethanol], and 1.2 to 5 μl of 100T zymolyase (10 mg/ml; US Biological, Z1005) was added to digest the cell wall. Cells were kept at 30°C until the cell walls were sufficiently digested (determined by counting the fraction of cells that lysed when placed in deionized water). Typically, lysis of 30 to 50% of cells was taken as evidence for sufficient digestion. Digestion was stopped by washing the cells three times with 1 ml of buffer B. Next, cells were incubated for 20 min in 1 ml of 0.01% Triton X-100 in 1× phosphate-buffered saline (PBS), followed by three more washes of buffer B and one wash with 10% formamide in 2× saline sodium citrate (SSC) buffer (Thermo Fisher Scientific, AM9770). Cells were resuspended in the formamide/SSC solution and split evenly into two replicate samples for hybridization. For each sample, 3.75 pmol of Stellaris RNA FISH probes (CAL Fluor red 610 probes targeting ymEGFP or *cdc13*, or Quasar 570 probes targeting *mad1* or *rpb1*; Biosearch Technologies, LGC) was combined with 2 μl each of salmon sperm DNA (Life Technologies, 15632-011) and yeast transfer RNA (Thermo Fisher Scientific, AM7119) and diluted in buffer F [20% formamide and 10 mM sodium phosphate buffer (pH 7.2)] to a final volume of 50 μl. This mixture was heated to 95°C for 3 min, allowed to cool to room temperature, and added to an additional 50 μl of buffer H [4× SSC buffer, acetylated bovine serum albumin (BSA) (4 mg/ml; Sigma-Aldrich, B8894), and 20 mM vanadyl ribonuclease complex]. Each sample was resuspended in 100 μl of this hybridization solution. When possible, as a positive control, one of the replicates of each sample was hybridized with probes for a higher abundance mRNA which would be expected to show FISH spots in all cells if the experiment was successful. After overnight incubation in the dark at 37°C, the hybridization solution was removed and cells were incubated for 6 min in 10% formamide/2× SSC heated to 37°C, 6 min in 0.1% Triton X-100/2× SSC, and lastly 10 min in 4′,6-diamidino-2-phenylindole (DAPI; 1 μg/ml) in 1× PBS. After DAPI staining, the cells were washed once with 1× PBS before final resuspension in 1× PBS. Cells were stored in the dark at 4°C until imaging.

For imaging, cells were mounted in SlowFade Diamond Antifade Mountant (Thermo Fisher Scientific, S36972) with #1.5 glass coverslips and ribonucleases-free slides. Cells were imaged with a Zeiss AxioImager M1 equipped with Xcite Fire light-emitting diode illumination (Excelitas), a Zeiss Plan FLUAR 100×/1.45 oil objective, and an ORCA-Flash4.0LT scientific complementary metal-oxide semiconductor (sCMOS) camera (Hamamatsu). FISH-optimized red or gold filters (Chroma 49306 and 49304, respectively) were used to image the FISH probes, while standard GFP, cyan fluorescent protein, and DAPI filters were used to capture images of GFP-labeled TSs, cell areas, and nuclei, respectively. The images for each channel consisted of a 6 μm *z* stack containing 31 images at 0.2 μm intervals.

### Image processing of smFISH

Images were dark noise-subtracted and flatfield-corrected. Cells were segmented in two dimensions using a custom FIJI macro based on using trainable Weka segmentation (*84*), while nuclei were segmented in three dimensions from DAPI images using a custom FIJI macro adapted from https://github.com/haesleinhuepf/cca_benchmarking (Robert Haase, MPI-CBG, Dresden; September 2019 version, accessed 21 September 2020). Cell segmentation was manually corrected and cells missing nuclear segmentation, or whose nuclei were not fully contained within the image stack, were removed. Cell length was measured from the segmented cell outlines using the bounding box method. Cells with lengths >4 SDs away from the sample mean were removed (zero to two cells per sample). RNA FISH spots and GFP TS spots were detected with FISHquant v3a (*85*). FISH or GFP images were filtered with the default 3D_LoG filter (size = 5, sigma = 1). Spots were initially found using local maximum detection and an automatically determined minimum intensity threshold; the quality score filtering option was not used. The default setting was used for the minimum distance between spots (160 nm). After point spread function (PSF) fitting, final thresholds were set manually for the PSF sigma *xy*, sigma *z*, and *z* position to exclude non-RNA or non-TS spots such as hot pixels. Spot detection accuracy was checked manually for a subset of cells from each image. Additional R scripts were used to classify each spot as nuclear or cytoplasmic based on the 3D nuclear DAPI segmentation.

In TS-labeling experiments, cells with a GFP spot in the cytoplasm, distant from the nucleus, were excluded from the analysis (only three cells across both strains and experiments). In addition, cells without a GFP spot or with more than two GFP spots per nucleus were excluded from the analysis. This removed 3 to 13% of the mononucleated cells and 15 to 37% of the binucleated cells. In the binucleated cells, this was typically due to the absence of a detectable GFP spot in one or both of the two nuclei. To identify nascent mRNA FISH spots, the 3D distance of each FISH spot to its nearest TS spot was determined. The distribution of distances was bimodal (fig. S16B), and a distance cutoff was used to separate nascent from mature mRNA. The distance cutoff was calculated separately for each image generated by a clustering method that locates the minimum between the two distance distributions.

In cells where a TS was located just outside the region initially segmented as nuclear based on DNA staining, the segmentation of the nucleus was isotropically expanded to include the TS (NucSegmentMethod = 3DnucExpand). This seemed justified, since nuclei in cells where this was the case were on average slightly smaller than those in cells where this was not the case, suggesting that the initial nuclear segmentation had not captured the entire nucleus. Alternatively, these cells were excluded from the analysis (NucSegmentMethod = CF, cells filtered), which did not change the overall results or conclusions.

The number of mRNA molecules per cell was calculated with three different approaches. Traditionally, counts of mRNA molecules per cell are obtained by counting the number of spots visible in the microscopy image (“spot counts”). This method is accurate as long as nearly all spots contain a single mRNA molecule. For the genes in this study, cytoplasmic spot intensity distributions are monomodal and relatively narrow, strongly suggesting that nearly all spots consist of single mRNA molecules (Fig. 7C and fig. S9B). In addition, for nuclear spots of low expressed genes (*mad1*, *mad2*, *mad3*, and *bub1*) and mature nuclear spots of both *mad1* and *rpb1*, the distribution of spot intensities is the same as in the cytoplasm, suggesting that these spots also consist of single mRNA molecules (Fig. 7C and fig. S9B). However, for the higher expressed genes in this study, the nuclear spot intensity distribution is wider than that of the cytoplasmic spots, with a notable fraction of nuclear spots having higher intensities than the cytoplasmic spots (*sep1*, *rpb1*, *cdc13*, *SPAC2H10.01*, and to a lesser extent, *SPAC27D7.09c*; for example, see fig. S9B). These brighter spots likely represent nascent mRNA at the TS (confirmed for *rpb1*; Fig. 7C), and the higher brightness is presumably due to multiple mRNA molecules being transcribed simultaneously. In this case, counting the number of spots while ignoring intensity (spot count) can underestimate abundance and variability in nuclear mRNA counts because TSs with varying numbers of mRNA molecules (e.g., two, three, or four molecules) are counted as one mRNA. To solve this problem, spot intensity can be incorporated into mRNA counts (intensity count method). First, the amplitude of each spot’s PSF is divided by the median amplitude of the cytoplasmic spots in each image. Since, for the genes in this study, the vast majority of spots in the cytoplasm can be assumed to consist of single mRNA molecules, the result is a normalized intensity measure in which a typical single mRNA FISH spot will have a value of 1 (fig. S9C). These normalized intensities are summed across all spots in the cell to produce an intensity-based estimate of the number of mRNA molecules per cell. While this method allows spots with multiple mRNAs to be counted as >1 mRNA molecule, the downside is that the intensities of individual spots show technical variation, which then finds its way into the counts. In the cytoplasm (i.e., single mRNA spots), the intensity coefficient of variation ranged from 0.24 to 0.37 and count values for individual spots typically spanned 0.53 to 1.77 molecules. Variation can arise from unequal staining or unequal illumination of the image. Thus, summing intensities introduces technical noise into the resulting counts of mRNA per cell and results in consistently higher cytoplasmic Fano factors compared to the spot count–based method (figs. S10 and S19). Therefore, we developed a third method (hybrid count) that combines features of both these methods (fig. S9C). If a spot has a normalized intensity below the 95th percentile of the cytoplasmic spots in that sample, then it is assumed to contain one mRNA molecule. For brighter spots, which likely contain multiple mRNA molecules, the number of molecules per spot is set equal to the normalized intensity (fig. S9C), i.e., equal to the count it would have received with the intensity count method. The molecule counts for all spots are then summed to obtain the number of mRNA molecules per cell. We consider hybrid count to be the most reliable method since, for single mRNA spots, it excludes technical noise from spot intensity variation, but it uses intensity information for brighter spots containing multiple mRNA molecules (e.g., *rpb1* TSs) where spot count fails to accurately estimate the number of molecules per spot. However, we also present Fano factors calculated with the spot count and intensity count methods for key results (figs. S10 and S19), and we used the spot count method when directly comparing our results to Marguerat and colleagues' data (*8*, *23*) (Fig. 3B and fig. S11D), since we did not have intensity values available for their data.

### Size-corrected Fano factor

Cell size–corrected Fano factors were calculated as by Padovan-Merhar and colleagues (*46*), using linear regression to assess the influence of cell size. We either used cell length as a proxy for cell size or we calculated volume from cell length and width, making the assumption that *S. pombe* cells are shaped like a cylinder with hemispheres at the two ends. The size parameter used did not qualitatively influence the results (figs. S11C and S18F).

To determine the Fano factor in a sliding window across cell sizes, cells within a window of 1 μm cell length were combined, and mRNA variance, mean, and Fano factor were calculated. The window was moved by 0.1 μm across all cell sizes. For mono- and binucleated cells combined, data were retained when there were at least 50 cells in the window for pooled data, or 40 cells in the window for single replicates, for at least 20 consecutive windows. For mononucleated cells, these threshold numbers were 40, 30, and 10 and, for binucleated cells, they were 30, 20, and 6.

### Analysis of published smFISH data

We reanalyzed smFISH data of *S. pombe* by Marguerat and colleagues (*8*, *23*), who had also determined both mRNA numbers and cell size. Genes that appeared cell cycle–regulated were excluded (*ace2*, *fkh2*, *mid2*, *SPAPB17E12.14c*, and *SPAPB1E7.04c*). One dataset (wt.1) for *rpb1* from Sun *et al*. (*23*) was excluded because 5% of the cells lacked any mRNA, whereas the highest percentage of cells without *rpb1* mRNA in any other dataset was 0.6%. A few cells across all datasets had highly unusual cell widths (smaller than 1.5 μm or larger than 4 μm) and were excluded. Cells with a length above 16 μm were excluded as well. The cell size–corrected Fano factor was calculated in the same way as for our data.

### Immunoblotting

Between 6 × 10^7^ and 1 × 10^8^ cells were washed with water, pelleted, resuspended in 500 μl of 0.22 M NaOH and 0.12 M beta-mercaptoethanol, and incubated on ice for 15 min. Trichloroacetic acid was added to a final concentration of 6% followed by another 10 min incubation on ice. The samples were pelleted, optionally washed with acetone, and either resuspended in 100 μl of 1× high urea (HU) buffer pH 6.8 (4 M urea, 2.5% SDS, 100 mM tris-HCl, 0.5 mM EDTA, 0.005% bromophenol blue, 10% glycerol, and 0.1 M dithiothreitol) or in 1× to 1.5× NuPage LDS sample buffer (Thermo Fisher Scientific, NP0007) with 5% beta-mercaptoethanol. Samples were heated to 70°C for 5 min and further lysed with glass beads (Sigma-Aldrich, G8772) for 1 to 2 min at 30 s^−1^ in a Retsch MM 400 ball mill. Extracts were collected by centrifugation and incubated at 70°C for another 3 min. Samples were run on NuPAGE 4 to 12% bis-tris polyacrylamide gels (Thermo Fisher Scientific) with MOPS running buffer and NuPAGE antioxidant. Proteins were transferred to Immobilon-P polyvinylidene difluoride membrane (Millipore Sigma) using an Amersham Semi-Dry Transfer Unit and transfer buffer (39 mM glycine, 48 mM tris base, and 5 to 10% methanol) containing 0.01% SDS and 1:1000 NuPAGE antioxidant. The membrane was blocked with 4% nonfat milk in tris-buffered saline with Tween 20 (TBS-T) (150 mM NaCl, 20 mM tris base, and 0.05% Tween-20) for 15 to 30 min at room temperature. Antibodies were in 4% nonfat milk in TBS-T, and membranes were washed with TBS-T between antibody incubation steps. Primary antibodies were mouse anti-GFP (0.4 μg/ml; Roche, 11814460001), rabbit anti-Cdc2 (0.2 μg/ml; Santa Cruz Biotechnology, SC-53), rabbit anti-Mad1 (*38*) (2 μg/ml), rabbit anti-Mad2 (against recombinant protein, serum at 1:1000), rabbit anti-Mad3 (*86*) (serum at 1:500), rabbit anti-Bub1 (serum at 1:2000; peptide: CKPKAGGPGRRRSSN); secondary antibodies were goat anti-mouse horseradish peroxidase (HRP) or goat anti-rabbit HRP (1:10,000; Dianova, 115-035-003 and 111-035-003). For imaging, membranes were incubated with SuperSignal West Dura Extended Duration Substrate or SuperSignal West Pico PLUS Chemiluminescent Substrate (Thermo Fisher Scientific) and chemiluminescence was recorded with a ChemiDoc XRS+ (Bio-Rad). Protein expression was quantified relative to each wild-type sample and relative to the loading control Cdc2, using the dilution series for linear regression.

### Quantitative PCR

RNA extraction, cDNA library preparation, and qPCR were performed as previously described (*39*). Briefly, cells were grown in either EMM (TATA-box mutant experiments), EMM with leucine (0.2 g/liter; one *mad1* deletion strain), or YEA (all other) to concentrations of 0.7 × 10^7^ to 1.5 × 10^7^ cells/ml, and then flash-frozen in liquid nitrogen. RNA extraction was followed by deoxyribonuclease (DNase) treatment and SuperScript IV reverse transcription with oligo d(T)_20_ primers. *act1* and *cdc2* were used as reference genes in all qPCRs. The average amplification efficiency was 93.2%. Relative expression was quantified from the mean Ct values of three (occasionally two) replicate wells. Three samples with high SD in Ct values across wells (>0.3) were excluded.

### Rapid amplification of cDNA ends PCR (RACE-PCR)

Cells were grown in YEA and snap-frozen in liquid nitrogen (~1 × 10^8^ cells). RNA was extracted using acidic phenol-chloroform and treated with DNase (Roche, DNase I) to remove any remaining DNA contamination. For 3′UTR sequencing, DNase-treated RNA was reverse-transcribed with the FirstChoice RLM-RACE kit (Invitrogen, AM1700). Either the FirstChoice RLM-RACE kit with SuperScript IV (Invitrogen) or the GeneRacer kit (Invitrogen, L150201) with SuperScript III (Invitrogen) was used to prepare the cDNA for 5′UTR sequencing. cDNA was amplified with two rounds of nested PCR targeting the gene of interest. The PCR product was inserted into a pBlueScript vector using Gibson assembly (NEB, E2611) and transformed into NEB 5-alpha Competent *E. coli* (NEB, C2992). Vectors were sequenced by Sanger sequencing. Sequences were discarded if the UTR appeared to begin (5′UTR) or end (3′UTR) in the coding sequence of the gene. When possible, to limit the analysis to mature mRNA, sequences were checked for the presence of introns and discarded if introns were found.

### SAC protein-GFP imaging in live cells

Cells were grown in EMM or EMM with leucine (0.2 g/liter) to between 4 × 10^6^ and 1.5 × 10^7^ cells/ml and mounted in µ-Slide 8 well glass bottom chambers (Ibidi, 80827) coated with lectin (50 μg/ml; Sigma-Aldrich, L1395). Cells were imaged on a DeltaVision Elite microscope equipped with a PCO edge sCMOS camera. Imaging was performed with an Olympus 60×/1.42 Plan APO oil objective and GFP filters. Images were taken as 7.2 μm *z* stacks with 0.1 μm step size. To bleach autofluorescence, an initial GFP image stack was acquired with 0.1 s exposures and discarded before the main GFP image stack was acquired using 0.25 to 0.40 s exposures (depending on the protein being imaged). GFP images were deconvolved using SoftWoRx software with two cycles of the ratio method (conservative) and a camera intensity offset of 50. Representative regions of cells were selected and single image slices from the *z* stack were extracted at approximately the midplane of the cells. Image intensity was further scaled in ImageJ and Photoshop, being consistent for each GFP-tagged protein.

### Gene Ontology enrichment analysis

Gene Ontology (GO) slim enrichment analysis was performed with the PANTHER overrepresentation test [www.pantherdb.org (*87*)] (released 02 February 2022), using the GO database (DOI:10.5281/zenodo.6399963; released 22 March 2022). From the data reported by Thodberg and colleagues (*49*), only transcripts of non–mitochondrial protein-coding genes were kept. For genes with multiple TSSs, only the TSS with the highest pooled TPM value was retained. Start codon positions were downloaded from PomBase (*88*) on 22 June 2022. All genes for which the TSS was located downstream of the currently annotated start codon were excluded. This resulted in a final list of 4648 genes, which was used as reference data for the GO enrichment analysis. Of those genes, 296 had a 5′UTR of 15 nt or shorter, and 451 had a 5′UTR of 20 nt or shorter. Only the most specific subclass from the GO slim analysis is shown. Fisher’s exact test with false discovery rate correction was used to identify statistically significant results.

### Generalized linear mixed models

Generalized linear mixed models were fit to counts of mRNA per cell using the R package lme4 (*89*). The spot count method was used for mRNA counts due to the need for integer counts. Counts were modeled as a function of cell length (natural log-transformed and centered), genotype, and the nested random effects strain, experimental replicate, and microscopy image. The natural log link function and Poisson error distribution were also assumed. A likelihood ratio test was used to test for the presence of an interaction between cell length and genotype. *P* values were derived by bootstrapping the null distribution (1000 bootstrapping replicates) using the R package pbkrtest. Significant interactions were found and thus included in the model for the following comparisons: *mad2* and *mad3* at the endogenous versus exogenous locus, and *mad2* versus *rad21* coding sequences at the exogenous locus. The 95% confidence intervals for the ratios of RNA abundance between pairs of genotypes and the 95% confidence bands for the regression curves were generated by bootstrapping (10,000 bootstrapping replicates).

## Supplementary Materials

### This PDF file includes:

Supplementary Text Figs. S1 to S19 Tables S1 to S25 Legends for data S1 to S5 References
