# Supplementary material for: The minimal intrinsic stochasticity of constitutively expressed eukaryotic genes is sub-Poissonian
Source: Sci Adv. 2023 Aug 9;9(32):eadh5138. doi: 10.1126/sciadv.adh5138 (PMC10411910; doi:10.1126/sciadv.adh5138)
Supplement: Supplementary file 1 — Supplementary Text Figs. S1 to S19 Tables S1 to S25 Legends for data S1 to S5 References [file sciadv.adh5138_sm.pdf]

Science Advances
AAAS

Supplementary Materials for

The minimal intrinsic stochasticity of constitutively expressed eukaryotic genes is sub-Poissonian

Douglas E. Weidemann *et al.*

Corresponding author: Silke Hauf, silke@vt.edu

Sci. Adv. **9**, eadh5138 (2023)
DOI: 10.1126/sciadv.adh5138

The PDF file includes:

Supplementary Text
Figs. S1 to S19
Tables S1 to S25
Legends for data S1 to S5
References

Other Supplementary Material for this manuscript includes the following:

Data S1 to S5

Supplementary Text

A. Statistical tests of size-corrected Fano factors

A.1 Statistical methodology

For statistical conclusions on the size-corrected Fano factors, we used a hierarchical bootstrapping approach. Experimental replicates (shown separately in the main figures) and cells within replicates were resampled with replacement. From this, the statistic of interest (average Fano factor across experimental replicates, difference between genotypes, average difference in Fano factor between mono- and binucleated cells, or average difference in Fano factor between cellular compartments) was calculated. To estimate the statistic's distribution, 5,000 bootstrap replicates were performed, and the mean and 95 % confidence interval (2.5^{th} to 97.5^{th} percentile) were calculated from the bootstrap results. Up to 0.5 % of bootstrap replicates were allowed to fail to calculate (i.e., returned NaN), and these were discarded before calculating the mean and confidence interval. If > 0.5 % of bootstrap replicates failed, no statistical results were reported for that analysis. The latter was only the case for mature nuclear mRNA from *mad1* in binucleated cells (transcription site [TS]-labeled data), due to a low cell number in one replicate. A genotype was considered to have a size-corrected RNA distribution significantly different from Poisson if the bootstrap 95 % confidence interval excluded 1, and differences were considered significant if the confidence interval excluded 0.

A.2 Comparison of hierarchical bootstrapping with bootstrapping from pooled data

As a comparison to hierarchical bootstrapping, cells from replicate experiments were combined and the cell-size corrected Fano factor calculated from the pooled set of cells. This yielded very similar results to the hierarchical bootstrapping results (Fig. S1, S3), except for some genes in published data (*8, 23*) with a larger variation between experimental replicates (Fig. S2).

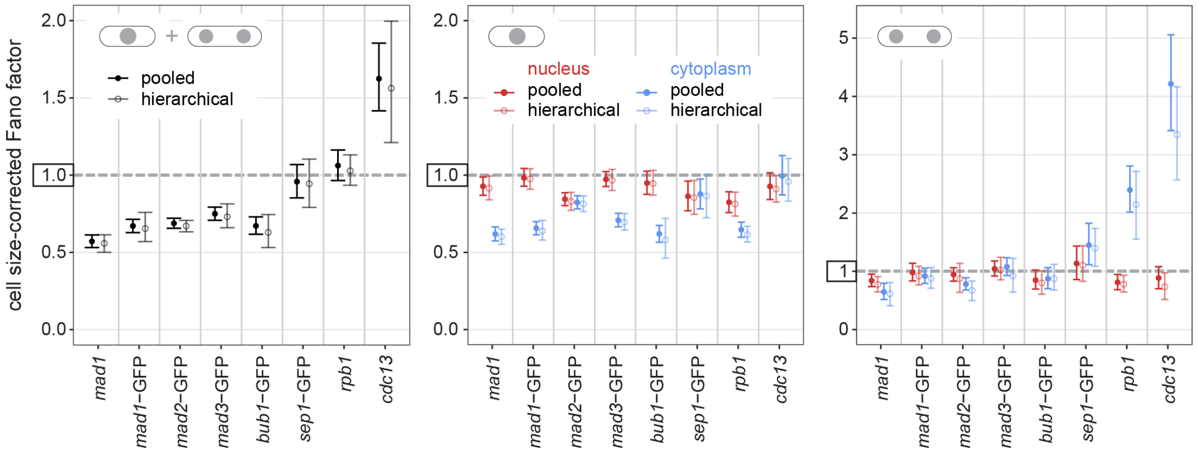

Figure S1: Fano factors from pooled and hierarchical analysis (non-TS labeled)
Related to Fig. 2, 3A, and 6F,G.

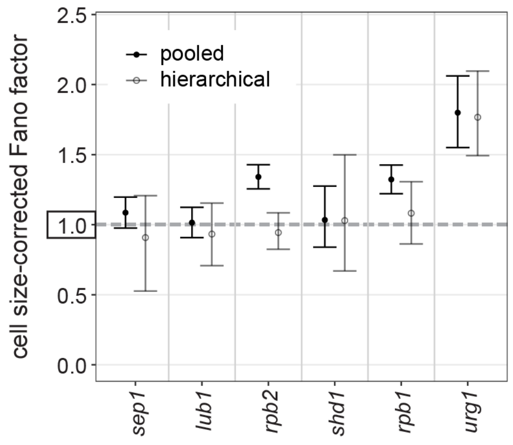

Figure S2: Fano factors from pooled and hierarchical analysis of Marguerat group data
Related to Fig. 3A. Only genes with more than one experimental replicate are shown.

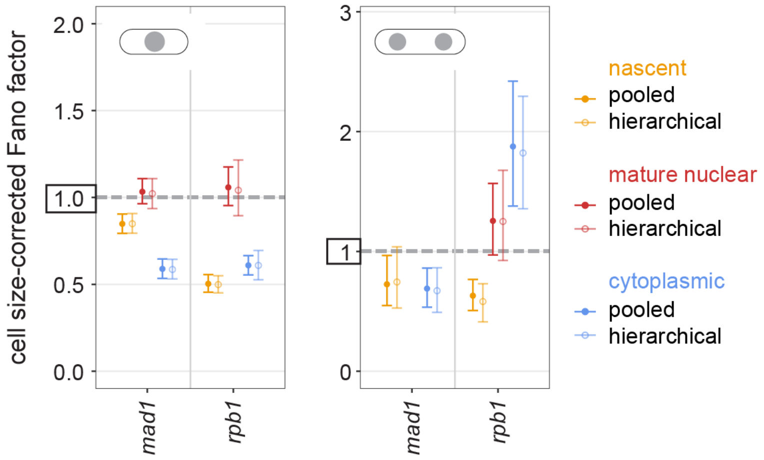

Figure S3: Fano factors from pooled and hierarchical analysis (TS-labeled)
Related to Fig. 7D.

A.3 Comparison of Fano factors in cellular compartments

We tested if size-corrected Fano factors differ between cellular compartments using hierarchical bootstrapping. Mean and 95 % confidence intervals from bootstrapping the mean difference between compartments are provided. Confidence intervals that exclude 0 are interpreted as significantly different Fano factors between compartments.

Table S1: Comparison between nuclear and cytoplasmic Fano factors (non-TS labeled)
Comparing nuclear and cytoplasmic Fano factors in experiments without transcription-site labeling (see Fig. 6 and S1). A positive mean Fano factor difference indicates a larger Fano factor in the cytoplasm than the nucleus, while a negative mean Fano factor difference indicates a smaller Fano factor in the cytoplasm than the nucleus.

Gene	Nuclei Count	Mean Fano Factor Difference	Lower 95 % Confidence Interval	Upper 95 % Confidence Interval	Effect
mad1	1	-0.31	-0.39	-0.23	smaller
mad1	2	-0.16	-0.38	0.06	same
mad1	all	-0.29	-0.37	-0.20	smaller
mad1-GFP	1	-0.34	-0.42	-0.25	smaller
mad1-GFP	2	-0.04	-0.25	0.18	same
mad1-GFP	all	-0.29	-0.37	-0.20	smaller
mad2-GFP	1	-0.02	-0.09	0.07	same
mad2-GFP	2	-0.20	-0.52	0.05	same
mad2-GFP	all	-0.04	-0.10	0.03	same
mad3-GFP	1	-0.27	-0.37	-0.18	smaller
mad3-GFP	2	-0.11	-0.53	0.26	same
mad3-GFP	all	-0.22	-0.33	-0.12	smaller
bub1-GFP	1	-0.37	-0.52	-0.20	smaller
bub1-GFP	2	0.07	-0.25	0.42	same
bub1-GFP	all	-0.26	-0.38	-0.13	smaller
sep1-GFP	1	0.01	-0.13	0.15	same
sep1-GFP	2	0.29	-0.11	0.69	same
sep1-GFP	all	0.06	-0.11	0.22	same
rpb1	1	-0.20	-0.29	-0.10	smaller
rpb1	2	1.37	0.72	1.99	larger
rpb1	all	0.18	0.05	0.30	larger
cdc13	1	0.05	-0.10	0.22	same
cdc13	2	2.62	1.90	3.37	larger
cdc13	all	0.66	0.30	1.09	larger
SPAC2H10.01-GFP	1	1.88	0.79	3.05	larger
SPAC2H10.01-GFP	2	1.31	0.61	1.95	larger
SPAC2H10.01-GFP	all	1.97	0.91	3.05	larger
SPAC27D7.09c-GFP	1	6.84	4.82	9.03	larger
SPAC27D7.09c-GFP	2	3.19	-1.93	7.89	same
SPAC27D7.09c-GFP	all	6.63	4.74	8.67	larger

Table S2: Comparison between nuclear and cytoplasmic Fano factors at the exogenous *wis1* locus

Comparing nuclear and cytoplasmic Fano factors at the exogenous *wis1* locus (see Fig. 5D). A positive mean Fano factor difference indicates a larger Fano factor in the cytoplasm than the nucleus, while a negative mean Fano factor difference indicates a smaller Fano factor in the cytoplasm than the nucleus. Change observed in mononucleated cells (as opposed to mononucleated and binucleated cells pooled = all) is indicated in the second effect column, without listing the mean Fano factor difference and confidence intervals.

Gene	Mean Fano Factor Difference	Lower 95 % Confidence Interval	Upper 95 % Confidence Interval	Effect all	Effect mono-nucl.
mad2 endog	-0.04	-0.11	0.03	same	same
mad3 endog	-0.23	-0.33	-0.12	smaller	smaller
rad21 endog	6.74	5.82	7.71	larger	larger
mad2 exog	0.02	-0.12	0.16	same	same
Pmad2-rad21 exog	0.24	0.02	0.46	larger	same
Pmad2-nmt1 exog	0.10	-0.06	0.26	same	same
mad3 exog	0.03	-0.12	0.17	same	same
Pmad3-rad21 exog	0.50	0.27	0.73	larger	same
Pmad3-nmt1 exog	0.26	0.03	0.49	larger	same

Table S3: Comparison of Fano factors in different compartments (TS-labeled)

Comparing nascent, mature nuclear, and cytoplasmic Fano factors in experiments with transcription-site labeling (see Fig. 7 and S3). A positive mean Fano factor difference indicates a larger Fano factor in compartment 2 than in compartment 1, while a negative mean Fano factor difference indicates a smaller Fano factor in compartment 2 than in compartment 1.

Gene	Nuclei Count	Compartment 1	Compartment 2	Mean Fano Factor Difference	Lower 95 % Confidence Interval	Upper 95 % Confidence Interval	Effect
mad1	1	Nascent	Mature Nuclear	0.17	0.07	0.28	larger
mad1	1	Nascent	Cytoplasm	-0.26	-0.34	-0.18	smaller
mad1	1	Mature Nuclear	Cytoplasm	-0.44	-0.54	-0.33	smaller
mad1	2	Nascent	Mature Nuclear	N/A	N/A	N/A	N/A
mad1	2	Nascent	Cytoplasm	-0.08	-0.45	0.23	same
mad1	2	Mature Nuclear	Cytoplasm	N/A	N/A	N/A	N/A
mad1	all	Nascent	Mature Nuclear	0.20	0.07	0.31	larger
mad1	all	Nascent	Cytoplasm	-0.23	-0.32	-0.15	smaller
mad1	all	Mature Nuclear	Cytoplasm	-0.42	-0.52	-0.32	smaller
rpb1	1	Nascent	Mature Nuclear	0.54	0.40	0.71	larger
rpb1	1	Nascent	Cytoplasm	0.11	0.02	0.21	larger
rpb1	1	Mature Nuclear	Cytoplasm	-0.43	-0.57	-0.31	smaller
rpb1	2	Nascent	Mature Nuclear	0.67	0.29	1.21	larger
rpb1	2	Nascent	Cytoplasm	1.23	0.75	1.72	larger
rpb1	2	Mature Nuclear	Cytoplasm	0.57	-0.14	1.19	same
rpb1	all	Nascent	Mature Nuclear	0.53	0.42	0.65	larger
rpb1	all	Nascent	Cytoplasm	0.48	0.28	0.70	larger
rpb1	all	Mature Nuclear	Cytoplasm	-0.06	-0.28	0.17	same

A.4 Comparison of Fano factors between mononucleated and binucleated cells

We tested if size-corrected Fano factors differ between mono- and binucleated cells using hierarchical bootstrapping. Mean and 95 % confidence intervals from bootstrapping the mean difference are provided. Confidence intervals that exclude 0 are interpreted as a significant difference in Fano factors.

Table S4: Comparison of Fano factors between binucleated and mononucleated cells (non-TS labeled)
Comparing Fano factors from mono- and binucleated cells in experiments without transcription-site labeling (see Fig. 6 and S1). A positive mean Fano factor difference indicates a larger Fano factor in binucleated than mononucleated cells, while a negative mean Fano factor difference indicates a smaller Fano factor in binucleated than mononucleated cells.

Gene	Compartment	Mean Fano Factor Difference	Lower 95 % Confidence Interval	Upper 95 % Confidence Interval	Effect
mad1	Nucleus	-0.14	-0.27	-0.02	smaller
mad1	Cytoplasm	0.01	-0.21	0.22	same
mad1-GFP	Nucleus	-0.06	-0.22	0.12	same
mad1-GFP	Cytoplasm	0.24	0.08	0.41	larger
mad2-GFP	Nucleus	0.05	-0.22	0.33	same
mad2-GFP	Cytoplasm	-0.14	-0.34	0.04	same
mad3-GFP	Nucleus	0.06	-0.13	0.31	same
mad3-GFP	Cytoplasm	0.22	-0.04	0.52	same
bub1-GFP	Nucleus	-0.14	-0.37	0.09	same
bub1-GFP	Cytoplasm	0.29	0.06	0.57	larger
sep1-GFP	Nucleus	0.25	-0.06	0.61	same
sep1-GFP	Cytoplasm	0.53	0.21	0.88	larger
rpb1	Nucleus	-0.04	-0.20	0.15	same
rpb1	Cytoplasm	1.53	0.91	2.11	larger
cdc13	Nucleus	-0.18	-0.41	0.08	same
cdc13	Cytoplasm	2.38	1.61	3.19	larger
SPAC2H10.01-GFP	Nucleus	-0.93	-1.80	-0.13	smaller
SPAC2H10.01-GFP	Cytoplasm	-1.47	-2.57	-0.46	smaller
SPAC27D7.09c-GFP	Nucleus	3.87	1.08	6.16	larger
SPAC27D7.09c-GFP	Cytoplasm	0.19	-5.79	5.38	same

Table S5: Comparison of Fano factors between binucleated and mononucleated cells (TS-labeled)
Comparing Fano factors from mono- and binucleated cells in experiments with transcription-site labeling (see Fig. 7D and S3). A positive mean Fano factor difference indicates a larger Fano factor in binucleated than mononucleated cells.

Gene	Compartment	Mean Fano Factor Difference	Lower 95 % Confidence Interval	Upper 95 % Confidence Interval	Effect
mad1	Nascent	-0.11	-0.32	0.18	same
mad1	Mature Nuclear	N/A	N/A	N/A	N/A
mad1	Cytoplasm	0.08	-0.12	0.29	same
rpb1	Nascent	0.08	-0.09	0.24	same
rpb1	Mature Nuclear	0.21	-0.18	0.75	same
rpb1	Cytoplasm	1.21	0.74	1.68	larger

A.5 Comparison of Fano factors between endogenous and exogenous locus and with different coding sequences

We tested if size-corrected Fano factors for *mad2* and *mad3* differ between endogenous and exogenous locus, and after exchanging the coding sequence, using hierarchical bootstrapping. Mean and 95 % confidence intervals from bootstrapping the mean difference are provided. Confidence intervals that exclude 0 are interpreted as a significant difference in Fano factors.

Table S6: Comparison of Fano factors for *mad2* and *mad3* between endogenous and exogenous locus and with different coding sequences

Comparing Fano factors for *mad2* and *mad3* between endogenous and exogenous locus, and at the exogenous locus after changing the coding sequence (see Fig. 5). A positive mean Fano factor difference indicates a larger Fano factor in Genotype 2 than Genotype 1. Change observed in mononucleated cells (as opposed to mononucleated and binucleated cells pooled = all) is indicated in the second Effect column, without listing the mean Fano factor difference and confidence intervals.

Genotype 1	Genotype 2	Compart-ment	Mean Fano Factor Difference	Lower 95 % Confidence Interval	Upper 95 % Confidence Interval	Effect all	Effect mono-nucl.
mad2 endog	*mad2* exog	Nucleus	0.02	-0.07	0.10	same	same
mad2 endog	*mad2* exog	Cytoplasm	0.08	-0.05	0.21	same	same
mad2 exog	*Pmad2-**rad21*** exog	Nucleus	0.12	-0.04	0.29	same	same
mad2 exog	*Pmad2-**rad21*** exog	Cytoplasm	0.34	0.14	0.55	larger	larger
mad2 exog	*Pmad2-**nmt1*** exog	Nucleus	0.10	-0.03	0.22	same	same
mad2 exog	*Pmad2-**nmt1*** exog	Cytoplasm	0.17	0.00	0.35	same	same
mad3 endog	*mad3* exog	Nucleus	0.00	-0.15	0.16	same	same
mad3 endog	*mad3* exog	Cytoplasm	0.26	0.14	0.38	larger	larger
mad3 exog	*Pmad3-**rad21*** exog	Nucleus	-0.07	-0.26	0.11	same	same
mad3 exog	*Pmad3-**rad21*** exog	Cytoplasm	0.39	0.17	0.62	larger	larger
mad3 exog	*Pmad3-**nmt1*** exog	Nucleus	-0.07	-0.27	0.12	same	same
mad3 exog	*Pmad3-**nmt1*** exog	Cytoplasm	0.16	-0.04	0.36	same	larger

B. Modeling and Inference

B.1 Model specification

Transcription is modelled by the set of (effective) reactions:

$$U_1 \to U_2 \to \cdots \to U_S \to U_1 + N$$
$$N \Rightarrow M_N$$

where N is the nascent RNA and M_N is the mature nuclear mRNA. We consider S gene states, U_i, where $i = 1, \ldots, S$. The free promoter state U_1 denotes the state in which no RNA polymerase (RNAP) is bound to the promoter. Once RNAP binds the free promoter, a closed complex between the two is formed (U_2). Downstream steps (U_3 to U_{S-1}) may include several long-lived intermediate states related to promoter opening and escape (*67*). The last state U_S is a promoter-proximal paused state; release from this state leads to the clearing of the promoter for new RNAP binding and the beginning of elongation of the nascent transcript (*68, 69*). This is modelled by the reaction $U_S \to U_1 + N$. After a time delay, elongation and termination finish, leading to mature nuclear mRNA M_N. The rate of switching from $U_i \to U_{i+1}$ is given by k_A, and the rate of initiating nascent mRNA production, $U_S \to U_1 + N$, is given by k_B. The time for the nascent RNA *(N)* to become mature mRNA *(*M_N*)* is considered fixed at some value T, i.e. the reaction $N \Rightarrow M_N$ is a delayed reaction (denoted by the double right arrow). The fixed delay (deterministic

elongation and termination) can be derived from a microscopic stochastic model of RNAP movement along the DNA template (*90*), and deterministic elongation has been observed in budding yeast (*19*). Similar models have been proposed (*33, 37, 91*). Note that in this model there is no transcriptionally inactive state in the sense that each state is associated with a particular stage of the transcription process.

Nuclear export is modelled by the reaction

$$M_N \to M_C^1$$

with rate k_C.. This process is modelled by a single rate-limiting step. M_C^1 is to be interpreted as the mRNA upon its entrance into the cytoplasm.

Degradation of mRNA in the cytoplasm is modelled by the chain of reactions

$$M_C^1 \to M_C^2 \to \cdots \to M_C^R \to \emptyset$$

Cytoplasmic mRNA degradation in eukaryotes is a complex multi-step process (*70*) that we simplify to the chain of reactions above. We are not aware of specific data for the number of rate-limiting steps R in the degradation process. In Deneke et al. (*71*), it was shown that choosing $R = 5$ can fit data in yeast well, though other values may provide an equally good fit. The rate of switching from $M_C^i \to M_C^{i+1}$ is given by k_D. Note that the measured cytoplasmic mRNA is assumed to be the sum of cytoplasmic mRNA at any stage of its cytoplasmic lifecycle, i.e. $M_C = \sum_{i=1}^{R} M_C^i$.

B.2 General properties of the model

The model is characterized by four rate constants k_A, k_B, k_C, and k_D, the number of rate-limiting steps in the initiation process (S), and the number of rate-limiting steps in cytoplasmic degradation (R).

The mean time between two successive nascent mRNA production events is given by:

$$T_{init} = \frac{S-1}{k_A} + \frac{1}{k_B}$$

The mean time for nuclear export is:

$$T_{expt} = \frac{1}{k_C}$$

The mean time for cytoplasmic degradation is:

$$T_{degr} = \frac{R}{k_D}$$

The chemical master equation of the model is difficult to solve exactly, even in steady-state conditions. This is not unexpected since exact solutions for the joint distributions of molecule numbers are only known for a handful of special cases (*92*). However, because all reactions are first order, the propensities of the chemical master equation are linear in the molecule numbers. This implies that the means, variances and covariances of all species can be obtained exactly in closed-form using the linear-noise approximation (*92*). The matrix form of this method (*93*) is particularly useful because it makes computations using a computer algebra system such as Mathematica easy for any number of species (which can be arbitrarily large for our model depending on the values of S and R).

As in the telegraph model of gene expression (*94*), in steady-state conditions the moments of nuclear and cytoplasmic mRNA are functions not of the absolute values of the rate constants but rather of the rate constants normalized by the degradation rate, i.e. k_A / k_D, k_B / k_D, k_C / k_D. Hence, without loss of generality we set $k_D = R$ such that T_{degr} is fixed to unity; in other words, this choice non-dimensionalizes time by dividing it by the mean time it takes for a cytoplasmic mRNA to decay.

It is straightforward to derive the first-order moments of nuclear and cytoplasmic mRNA numbers per gene copy. These are respectively given by:

$$\mu_N = \frac{k_A k_B}{k_C(k_A + k_B(S-1))} \text{ and } \mu_C = \frac{k_A k_B}{k_A + k_B(S-1)}.$$

The closed-form solutions for the second-order moments of mRNA numbers were obtained using Mathematica. They are very complex and hence we do not show them here. Instead, we state properties of the stochastic model that follow from an analysis of these solutions.

For all parameters, the Fano factors of nuclear mRNA and of cytoplasmic mRNA are found to be less than or equal to 1. For $S = 1$, they are exactly equal to 1. As shown in previous publications (*33, 37*), the crucial ingredient of the model that leads to sub-Poisson noise is the switching of the promoter state upon production of a single nascent transcript, changing it from a state where no new RNAP can bind to a state where this is possible. This captures the observation that a promoter can only bind a new RNAP if there is no other bound RNAP within a short distance from it (*95, 96*). Mathematically, the switching of the promoter state causes sub-Poisson noise in the transcript numbers because the coefficient of variation of the time between two successive nascent mRNA production events is less than 1, i.e. less than expected from an exponential distribution.

Note that if there was no state switching upon the production of a nascent transcript

$$U_S \to U_1 \to U_2 \to \cdots \to U_S \to U_S + N$$

then the Fano factors of nascent, nuclear and cytoplasmic mRNA would be greater than or equal to 1 (*97, 98*). In models of this type, the interpretation is that U_1 to U_{S-1} are transcriptional off states while U_S is a transcriptionally active state. Nascent mRNA can be continuously produced from this state and there is no associated promoter clearing.

In steady-state conditions, the dynamics of nascent mRNA elongation and release do not influence the moments of the nuclear and cytoplasmic mRNA in our model. This is because we are assuming that release happens a certain fixed time T after initiation of elongation, and hence the time between two subsequent nascent mRNA production events is the same as the time between two subsequent mature nuclear mRNA production events. In other words, the moments of nuclear and cytoplasmic mRNA in steady-state conditions remain the same if we instead modeled transcription as

$$U_1 \to U_2 \to \cdots \to U_S \to U_1 + M_N$$

where the last reaction has rate constant k_B.

In steady-state conditions, the Fano factor of cytoplasmic mRNA can either be larger or smaller than the Fano factor of nuclear mRNA (Fig. 8C). From the closed-form solutions it is found that in the limit k_C goes to infinity, the Fano factor of cytoplasmic mRNA is smaller than that of nuclear mRNA. The exact value of k_C when the two Fano factors are equal depends on the values of k_A, k_B, S, and R (e.g., Table S7).

Table S7: Nuclear export rate k_C above which the Fano factor of cytoplasmic mRNA becomes smaller than the Fano factor of nuclear mRNA
These are computed from the analytical expression of the model assuming $k_A = k_B = 10$.

	$R = 1$	$R = 2$	$R = 3$	$R = 4$
$S = 2$	4.58	2.60	2.14	1.94
$S = 3$	3.87	2.38	2.01	1.84
$S = 4$	3.39	2.21	1.89	1.75

The minimum Fano factor of nuclear mRNA is achieved when $k_B = k_A \gg k_C$, in other words when the rates for promoter remodeling and the rate of initiating nascent mRNA production are in the same range and considerably faster than export. The minimum Fano factor of cytoplasmic mRNA is achieved in the limits $k_C \gg k_B = k_A \gg k_D$, in other words when the rates for promoter remodeling and the rate of initiating nascent mRNA production are in the same range and considerably faster than degradation but slower than export. The minimum values possible for a model with *S* and *R* each between 1 and 4 are shown in Table S8 and Fig. 8B.

Table S8: Minimum Fano factor of nuclear and cytoplasmic mRNA
Minimum Fano factors for the general model with *S* and *R* rate limiting steps in initiation and decay, respectively. The first number shows the nuclear Fano factor, the second number shows the cytoplasmic Fano factor.

	$R = 1$	$R = 2$	$R = 3$	$R = 4$
$S = 1$	(1.00,1.00)	(1.00,1.00)	(1.00,1.00)	(1.00,1.00)
$S = 2$	(0.750,0.750)	(0.750,0.688)	(0.750,0.656)	(0.750,0.637)
$S = 3$	(0.667,0.667)	(0.667,0.583)	(0.667,0.542)	(0.667,0.516)
$S = 4$	(0.625,0.625)	(0.625,0.531)	(0.625,0.484)	(0.625,0.455)

B.3 Analytical results for the covariance and Fano factors of nuclear and cytoplasmic mRNA

Because the propensities of the reaction system are linear, it is straightforward to compute analytical expressions for the means, variances and covariances of nuclear and cytoplasmic mRNA by direct solution of the moment equations (*92*). Consider the following four models, each of which has only one degradation step for cytoplasmic mRNA.

$$U_1 \overset{k_A}{\to} U_2 \overset{k_B}{\to} U_1 + M_N, M_N \overset{k_C}{\to} M_C^1 \overset{k_D}{\to} \emptyset \quad \text{(Model A)}$$

$$U_1 \overset{k_A}{\to} U_1 + M_N, M_N \overset{k_C}{\to} M_C^1 \overset{k_D}{\to} \emptyset \quad \text{(Model B)}$$

$$U_0 \underset{k_E}{\overset{k_F}{\leftrightharpoons}} U_1 \overset{k_A}{\to} U_1 + M_N, M_N \overset{k_C}{\to} M_C^1 \overset{k_D}{\to} \emptyset \quad \text{(Model C)}$$

$$U_0 \underset{k_E}{\overset{k_F}{\leftrightharpoons}} U_1 \overset{k_A}{\to} U_2 \overset{k_B}{\to} U_1 + M_N, M_N \overset{k_C}{\to} M_C^1 \overset{k_D}{\to} \emptyset \quad \text{(Model D)}$$

Model A is the $S = 2$, $R = 1$ model described earlier. Model B is Model A but without the intermediate state U_2, which is identical to the standard model for constitutive expression. Model C is Model B but with the addition of an inactive state U_0—this is the standard telegraph model

of gene expression (*94*). Model D is Model C with the addition of an intermediate state U_2; it can also be seen as Model A with the addition of an inactive state U_0. Model D is the most general model of the four since the rest can be seen as special cases of this model.

Setting $k_D = 1$ (so that the cytoplasmic mRNA degradation time equals 1) and solving the moment equations of the reaction system in steady-state conditions for each of these circuits, we obtain the following formula for the covariance of the nuclear and cytoplasmic mRNA and for the Fano factors of nuclear and cytoplasmic mRNA:

$$\text{Covar}(M_N, M_C^1) = -\frac{k_A^2 k_B^2 (1+k_A+k_B+k_C)}{(k_A+k_B)^2(1+k_A+k_B)(1+k_C)(k_A+k_B+k_C)} \quad \text{(Model A)}$$

$$\text{Covar}(M_N, M_C^1) = 0 \quad \text{(Model B)}$$

$$\text{Covar}(M_N, M_C^1) = \frac{k_E k_A^2 k_F (1+k_E+k_C+k_F)}{(1+k_C)(k_E+k_F)^2(1+k_E+k_F)(k_E+k_C+k_F)} \quad \text{(Model C)}$$

$$FF_N = 1 - \frac{k_A k_B}{(k_A+k_B)(k_A+k_B+k_C)} \quad \text{(Model A)}$$

$$FF_N = 1 \quad \text{(Model B)}$$

$$FF_N = 1 + \frac{k_A k_F}{(k_E+k_F)(k_E+k_C+k_F)} \quad \text{(Model C)}$$

$$FF_C = 1 - \frac{k_A k_B k_C (1+k_A+k_B+k_C)}{(k_A+k_B)(1+k_A+k_B)(1+k_C)(k_A+k_B+k_C)} \quad \text{(Model A)}$$

$$FF_C = 1 \quad \text{(Model B)}$$

$$FF_C = 1 + \frac{k_A k_C k_F (1+k_E+k_C+k_F)}{(1+k_C)(k_E+k_F)(1+k_E+k_F)(k_E+k_C+k_F)} \quad \text{(Model C)}$$

For Model D, the expressions are too complicated to show. The main properties are that the covariance can be positive or negative and that the Fano factors can be less than or greater than 1. If there is no inactive state (U_0) and the intermediate state (U_2) is long lived then Model D becomes Model A (the main model in this paper) which implies that the covariance is negative and the Fano factors are less than 1. If there is an inactive state and the intermediate state (simulating promoter-proximal pausing) is short lived then Model D becomes Model C (the standard telegraph model) which implies that the covariance is positive and the Fano factors are greater than 1. Thus Model A explains *mad1*, *mad1*-GFP, *mad2*-GFP, *mad3*-GFP, *bub1*-GFP, *sep1*-GFP, and *rpb1* while Model C explains *cdc13*, *SPAC2H10.01*-GFP, and *SPAC27D7.09c*-GFP (Fig. 6, 8F).

B.4 <u>Analytical results for the effect of multi-step degradation on the cytoplasmic Fano factor</u>

Consider the following two-step degradation versions of Models A and C in the previous section

$$U_1 \overset{k_A}{\rightarrow} U_2 \overset{k_B}{\rightarrow} U_1 + M_N, M_N \overset{k_C}{\rightarrow} M_C^1 \overset{k_D}{\rightarrow} M_C^2 \overset{k_D}{\rightarrow} \emptyset \quad \text{(Model E)}$$

$$U_0 \underset{k_E}{\overset{k_F}{\leftrightarrows}} U_1 \overset{k_A}{\rightarrow} U_1 + M_N, M_N \overset{k_C}{\rightarrow} M_C^1 \overset{k_D}{\rightarrow} M_C^2 \overset{k_D}{\rightarrow} \emptyset \quad \text{(Model F)}$$

Setting $k_D = 2$ so that the total cytoplasmic mRNA degradation time equals 1 and solving the moment equations for these reaction systems, one finds the following results for the Fano factors of the total cytoplasmic mRNA (defined as the sum of M_C^1 and M_C^2)

$$(FF_C)_{Model\,E} - (FF_C)_{Model\,A} = -\frac{k_A k_B k_C^2 (36 + k_A^2(5 + k_C) + k_B^2(5 + k_C) + 5k_C(5 + k_C) + k_B(5 + k_C)^2 + k_A(5 + k_C)(5 + 2k_B + k_C))}{4(1 + k_A + k_B)(2 + k_A + k_B)^2(1 + k_C)(2 + k_C)^2(k_A + k_B + k_C)}$$

$$(FF_C)_{Model\,F} - (FF_C)_{Model\,C} = \frac{k_A k_C^2 k_F (36 + k_\mathrm{E}^2(5 + k_C) + k_C^2(5 + k_F) + 5k_F(5 + k_F) + k_C(5 + k_F)^2 + k_\mathrm{E}(5 + k_C)(5 + k_C + 2k_F))}{4(1 + k_C)(2 + k_C)^2(1 + k_\mathrm{E} + k_F)(2 + k_\mathrm{E} + k_F)^2(k_\mathrm{E} + k_C + k_F)}$$

Note that the addition of one extra step of degradation to Model A (resulting in Model E) reduces the Fano factor of total cytoplasmic mRNA while the addition of one extra step of degradation to Model C (resulting in Model F), increases the Fano factor of total cytoplasmic mRNA. The latter has been previously shown in (*15*) while the former is a novel result. Another way of stating this is that if the fluctuations are sub-Poissonian then additional degradation steps reduce the Fano factor of cytoplasmic mRNA whereas the opposite occurs if the fluctuations are super-Poissonian.

B.5 Bayesian model selection and parameter inference

While it is immediately clear from Table S8 that models with one rate limiting step for transcription will not be able to explain the sub-Poisson nature of the data, it is an open question which of the models with two or more rate-limiting steps in transcription and one or more rate-limiting steps in degradation best fits the data. There are several approaches that in principle can be used for model selection. Broadly speaking, these can be divided into two main categories: methods that use only the information about the moments of mRNA and those that use the full distribution of mRNA counts. Generally, the latter leads to more accurate estimates (*99*). An example of such a method is maximum likelihood, computed using the finite state projection method. However, the issue with the use of such a method is that we do not have a likelihood that explicitly takes into account cell size—the likelihood is the solution of the chemical master equation and this can only be computed if one knows which reaction rate in our model has size dependence (which we do not). On the other hand since we have size-corrected variances (the correction method developed in (*46*) does not require us to know which rate is size dependent), we can use moment-based approaches.

Specifically, we used a simple Approximate Bayesian Computation (ABC) rejection sampler (*100*) to perform Bayesian model selection (*101*). For each gene and for a given model with certain values of S and R, the ABC algorithm randomly samples parameters k_A, k_B, k_C from a prior distribution. Since we do not have prior knowledge about the three parameters, we simply choose the distribution to be uniform over a certain range. Stochastic simulations of the model with the selected parameters are run using Gillespie's exact algorithm (*102*) until steady-state is achieved. Then an objective function between the moments of the data and the model is evaluated. Note that the number of samples of Gillespie's algorithm equals the number of single-cell measurements for a given gene. This constraint makes sure that the simulation-generated moments have the same variability (due to finite number of samples) as the moments calculated from the data. Those parameters whose objective function evaluates to less than a certain threshold are accepted. The mean and standard deviation of the marginal distribution of these accepted parameters (the posterior) provides an estimate of the values of the parameters and their uncertainty. We also calculate the acceptance rate, which is the number of parameter sets that are accepted divided by the total number of parameter sets that were randomly generated by the

ABC algorithm. We then select which model best selects the data for a given gene by comparing the acceptance rate of any two models. The Bayes factor of two models A and B is defined as the ratio of the acceptance rate of the two models (*101*). If the Bayes factor is larger than 10, we conclude that there is strong evidence in favor of model A; if the Bayes factor is less than 1/10, we conclude that there is strong evidence in favor of model B. Otherwise, if the Bayes factor is between 1/10 and 10, we conclude that there is no strong evidence for model A or model B (there might be weak evidence).

The heart of the ABC algorithm involves accepting only those parameters which satisfy a user-defined criterion. In our case the criterion is that the following objective function is smaller than a preset threshold value:

$$d = \left(\frac{2\mu_C - \mu_C^e}{\mu_C^e}\right)^2 + \left(\frac{FF_C - FF_C^e}{FF_C^e}\right)^2 + \left(\frac{FF_N - FF_N^e}{FF_N^e}\right)^2$$

where μ_C and FF_C are the simulation predictions for the mean and Fano factor (variance divided by the mean) of the cytoplasmic mRNA, M_C; and FF_N is the simulation prediction for the Fano factor of the nuclear mRNA, M_N. The same variables but with an e superscript denote the experimental estimates of the mean and the Fano factors for non-transcription site (non-TS) labeled data from mononucleated cells. Note that in this section whenever we refer to the Fano factor, we always mean the cell size-corrected value (see Methods). This is necessary since the model's predictions (with which the experimental values will be compared) are independent of the cell size. Note also that the simulation predictions are for a single gene copy. Mononucleated *S. pombe* cells are almost exclusively in G2 phase of the cell cycle and hence have two (independent) gene copies (*59*). Therefore, we multiply the model's estimate of the mean by 2 in the expression for the objective function. The Fano factor is unchanged because both the mean and variance are doubled.

Note that the objective function does not have information about the mean of nuclear mRNA but only about its Fano factor. This choice is motivated by a direct comparison of fitting to the data with or without the transcription site label for the two genes, *mad1* and *rpb1*. When the transcription site is not labeled (non-TS labeled), it is uncertain which fraction of the nuclear RNA is nascent RNA and which fraction is mature nuclear RNA. Not making this distinction leads to a considerably different estimate for the mean of nuclear RNA, μ_N, from non-TS labeled data compared to that estimated from TS-labeled data (Table S9). The relative error is around 200 %. In contrast, estimating the nuclear Fano factor, FF_N, is more robust (relative error less than 20 %). Furthermore, the estimates for both cytoplasmic mean and cytoplasmic Fano factor are similar, regardless of which data is used. We therefore excluded the mean of nuclear RNA, μ_N, from the objective function.

Table S9: Comparison of the means (μ) and Fano factors (FF) of nuclear (N) and cytoplasmic (C) mRNA

Parameters were estimated using both non-TS labeled and TS-labeled data for both genes. The largest discrepancies are observed for μ_N (red).

	μ_N		μ_C		FF_N		FF_C	
	non-TS labeled	TS-labeled	non-TS labeled	TS-labeled	non-TS labeled	TS-labeled	non-TS labeled	TS-labeled
mad1	0.318	0.120	1.74	1.74	0.929	0.917	0.620	0.540
rpb1	3.56	1.15	17.9	15.7	0.826	0.939	0.647	0.560

The only remaining information to be specified for the ABC rejection sampler is the choice of the ranges over which the parameters are randomly sampled and the threshold values (d_c) of the objective function for each gene (Table S10). Theoretically it is known that the distribution of accepted parameters from an ABC rejection sampler will be a good approximation to the true posterior distribution provided the threshold value is sufficiently small (*101*). A threshold value is hence chosen small enough so that repeating with a smaller value does not significantly change the posterior; this value will necessarily be different from one gene to another, particularly given the variable sample sizes. The range of the uniform prior distribution was initially chosen to be (0,250) for k_A, k_B, k_C. We noticed that in many cases initial runs of the ABC rejection sampler identified parameters satisfying the user-defined criterion only in small regions of this range. Hence to obtain a large number of accepted parameters within a computationally reasonable time (which is needed to obtain a well-defined posterior distribution) it was necessary to limit the range of the prior distributions to a subset of numbers within (0,250) which the initial exploration identified as containing the vast majority of the accepted parameters. We implemented the constraint that, for a given parameter set, the 12 models should be indistinguishable with respect to the mean nuclear and cytoplasmic mRNA. This is achieved by scaling k_A by a factor proportional to $S - 1$ (following from the equations for the first-order moments above).

Table S10: Priors for parameters k_A, k_B, k_C, and thresholds for the objective function (d_c)
Note that $k_D = R$.

Gene	k_A	k_B	k_C	d_c
mad1	$\frac{S-1}{2}(0.5,10.5)$	(0.5,10.5)	(0.5,100.5)	0.003
mad1-GFP	$\frac{S-1}{2}(0.5,10.5)$	(0.5,40.5)	(20,220)	0.0001
mad2-GFP	$\frac{S-1}{2}(0.5,35.5)$	(0.5,10.5)	(0.5,20.5)	0.005
mad3-GFP	$\frac{S-1}{2}(0.5,20.5)$	(0.5,10.5)	(20,220)	0.00025
bub1-GFP	$\frac{S-1}{2}(0.5,20.5)$	(0.5,75.5)	(20,220)	0.0001
rpb1	$\frac{S-1}{2}(25,75)$	(0.5,30.5)	(0.5,50.5)	0.00075
sep1-GFP	$\frac{S-1}{2}(0.5,100.5)$	(0.5,5.5)	(0.1,30.1)	0.00075

The Bayes factors were computed for all genes except *cdc13* (whose RNA counts are consistent with a Poisson distribution; Fig. S1) and *SPAC2H10.01*-GFP and *SPAC27D7.09c*-GFP (super-Poisson; Fig. 6), and for all 12 models with $2 \leq S \leq 4$ and $1 \leq R \leq 4$. The results are shown in Tables S11 to S17. For a given gene, the models highlighted in yellow are those which can explain the data, i.e. no other model was found to have an acceptance rate at least 10-times larger; or, in other words, all the 12 Bayes factors for this model are greater than or equal to 1/10. The model selection results are summarized in Table S18. We find strong evidence that *mad1*, *mad1*-GFP, and *bub1*-GFP are best described by models with 3 or 4 rate-limiting steps in transcription and 2–4 rate-limiting steps in degradation. In contrast, for the rest of the genes (*mad2*-GFP, *mad3*-GFP, *rpb1*, and *sep1*-GFP), most of the 12 models fit the data equally well. We also note that the models with $S = 3$ and $R = 3$ or 4, as well as $S = 4$ and $R = 2$, are the *only* models that can explain the data from all genes.

Table S11: Bayes factors for *mad1*

The value in the ith row and j^{th} column is the Bayes factor of models i and j. Bayes factors ≥ 10 are shown in blue (model i explains the data better than model j); Bayes factors ≤ 1/10 are shown in red (model j explains the data better than model i); Bayes factors in the range (1/10,10) are shown in black, which is interpreted as the two models being indistinguishable. Those models which explain the data best, i.e. those lacking Bayes factors ≤ 1/10, are shaded in yellow.

mad1	S2_R1	S2_R2	S2_R3	S2_R4	S3_R1	S3_R2	S3_R3	S3_R4	S4_R1	S4_R2	S4_R3	S4_R4
**S2_R1**	1.0E+00	1.0E+00	1.0E+00	2.3E+00	1.3E+00	5.8E+02	4.3E+03	8.1E+03	6.5E+01	9.0E+03	9.5E+03	4.8E+03
**S2_R2**	1.0E+00	1.0E+00	1.0E+00	2.3E+00	1.3E+00	5.8E+02	4.3E+03	8.1E+03	6.5E+01	9.0E+03	9.5E+03	4.8E+03
**S2_R3**	1.0E+00	1.0E+00	1.0E+00	2.3E+00	5.7E-01	5.8E+02	4.3E+03	8.1E+03	6.5E+01	9.0E+03	9.5E+03	4.8E+03
**S2_R4**	4.4E-01	4.4E-01	4.4E-01	1.0E+00	5.7E-01	2.5E+02	1.9E+03	3.5E+03	2.8E+01	3.9E+03	4.2E+03	2.1E+03
**S3_R1**	7.8E-01	7.8E-01	7.8E-01	1.8E+00	1.0E+00	4.5E+02	3.3E+03	6.3E+03	5.0E+01	7.0E+03	7.4E+03	3.8E+03
**S3_R2**	1.7E-03	1.7E-03	1.7E-03	4.0E-03	2.2E-03	1.0E+00	7.4E+00	1.4E+01	1.1E-01	1.6E+01	1.7E+01	8.4E+00
**S3_R3**	2.3E-04	2.3E-04	2.3E-04	5.3E-04	3.0E-04	1.3E-01	1.0E+00	1.9E+00	1.5E-02	2.1E+00	2.2E+00	1.1E+00
**S3_R4**	1.2E-04	1.2E-04	1.2E-04	2.8E-04	1.6E-04	7.1E-02	5.3E-01	1.0E+00	8.0E-03	1.1E+00	1.2E+00	6.0E-01
**S4_R1**	1.5E-02	1.5E-02	1.5E-02	3.5E-02	2.0E-02	8.9E+00	6.6E+01	1.2E+02	1.0E+00	1.4E+02	1.5E+02	7.5E+01
**S4_R2**	1.1E-04	1.1E-04	1.1E-04	2.5E-04	1.4E-04	6.4E-02	4.8E-01	9.0E-01	7.2E-03	1.0E+00	1.1E+00	5.4E-01
**S4_R3**	1.0E-04	1.0E-04	1.0E-04	2.4E-04	1.4E-04	6.0E-02	4.5E-01	8.5E-01	6.8E-03	9.4E-01	1.0E+00	5.1E-01
**S4_R4**	2.1E-04	2.1E-04	2.1E-04	4.7E-04	2.7E-04	1.2E-01	8.9E-01	1.7E+00	1.3E-02	1.9E+00	2.0E+00	1.0E+00

Table S12: Bayes factors for *mad1*-GFP

mad1-GFP	S2_R1	S2_R2	S2_R3	S2_R4	S3_R1	S3_R2	S3_R3	S3_R4	S4_R1	S4_R2	S4_R3	S4_R4
**S2_R1**	1.0E+00	1.0E+00	1.9E+01	4.7E+01	1.3E+01	6.4E+02	9.9E+02	8.8E+02	4.0E+02	2.4E+02	7.4E+01	6.6E+01
**S2_R2**	1.0E+00	1.0E+00	1.9E+01	4.7E+01	1.3E+01	6.4E+02	9.9E+02	8.8E+02	4.0E+02	2.4E+02	7.4E+01	6.6E+01
**S2_R3**	5.1E-02	5.1E-02	1.0E+00	2.4E+00	6.6E-01	3.3E+01	5.1E+01	4.5E+01	2.0E+01	1.2E+01	3.8E+00	3.4E+00
**S2_R4**	2.1E-02	2.1E-02	4.2E-01	1.0E+00	2.7E-01	1.4E+01	2.1E+01	1.9E+01	8.4E+00	5.0E+00	1.6E+00	1.4E+00
**S3_R1**	7.8E-02	7.8E-02	1.5E+00	3.6E+00	1.0E+00	5.0E+01	7.7E+01	6.8E+01	3.1E+01	1.8E+01	5.8E+00	5.1E+00
**S3_R2**	1.6E-03	1.6E-03	3.0E-02	7.3E-02	2.0E-02	1.0E+00	1.5E+00	1.4E+00	6.1E-01	3.7E-01	1.2E-01	1.0E-01
**S3_R3**	1.0E-03	1.0E-03	2.0E-02	4.7E-02	1.3E-02	6.5E-01	1.0E+00	8.9E-01	4.0E-01	2.4E-01	7.5E-02	6.7E-02
**S3_R4**	1.1E-03	1.1E-03	2.2E-02	5.3E-02	1.5E-02	7.3E-01	1.1E+00	1.0E+00	4.5E-01	2.7E-01	8.4E-02	7.5E-02
**S4_R1**	2.5E-03	2.5E-03	4.9E-02	1.2E-01	3.3E-02	1.6E+00	2.5E+00	2.2E+00	1.0E+00	5.9E-01	1.9E-01	1.7E-01
**S4_R2**	4.3E-03	4.3E-03	8.3E-02	2.0E-01	5.5E-02	2.7E+00	4.2E+00	3.7E+00	1.7E+00	1.0E+00	3.2E-01	2.8E-01
**S4_R3**	1.3E-02	1.3E-02	2.6E-01	6.3E-01	1.7E-01	8.7E+00	1.3E+01	1.2E+01	5.3E+00	3.2E+00	1.0E+00	8.9E-01
**S4_R4**	1.5E-02	1.5E-02	2.9E-01	7.1E-01	1.9E-01	9.7E+00	1.5E+01	1.3E+01	6.0E+00	3.6E+00	1.1E+00	1.0E+00

Table S13: Bayes factors for *mad2*-GFP

mad2-GFP	S2_R1	S2_R2	S2_R3	S2_R4	S3_R1	S3_R2	S3_R3	S3_R4	S4_R1	S4_R2	S4_R3	S4_R4
**S2_R1**	1.0E+00	8.9E-01	7.4E-01	6.6E-01	6.0E-01	4.2E-01	3.5E-01	3.4E-01	5.3E-01	4.3E-01	3.8E-01	3.5E-01
**S2_R2**	1.1E+00	1.0E+00	8.4E-01	7.4E-01	6.8E-01	4.7E-01	3.9E-01	3.8E-01	6.0E-01	4.8E-01	4.2E-01	3.9E-01
**S2_R3**	1.3E+00	1.2E+00	1.0E+00	8.8E-01	8.1E-01	5.7E-01	4.7E-01	4.5E-01	7.1E-01	5.8E-01	5.1E-01	4.6E-01
**S2_R4**	1.5E+00	1.4E+00	1.1E+00	1.0E+00	9.2E-01	6.4E-01	5.3E-01	5.2E-01	8.1E-01	6.6E-01	5.8E-01	5.3E-01
**S3_R1**	1.7E+00	1.5E+00	1.2E+00	1.1E+00	1.0E+00	7.0E-01	5.8E-01	5.6E-01	8.8E-01	7.2E-01	6.3E-01	5.8E-01
**S3_R2**	2.4E+00	2.1E+00	1.8E+00	1.6E+00	1.4E+00	1.0E+00	8.3E-01	8.0E-01	1.3E+00	1.0E+00	9.0E-01	8.2E-01
**S3_R3**	2.9E+00	2.5E+00	2.1E+00	1.9E+00	1.7E+00	1.2E+00	1.0E+00	9.7E-01	1.5E+00	1.2E+00	1.1E+00	9.9E-01
**S3_R4**	3.0E+00	2.6E+00	2.2E+00	1.9E+00	1.8E+00	1.2E+00	1.0E+00	1.0E+00	1.6E+00	1.3E+00	1.1E+00	1.0E+00
**S4_R1**	1.9E+00	1.7E+00	1.4E+00	1.2E+00	1.1E+00	7.9E-01	6.6E-01	6.4E-01	1.0E+00	8.1E-01	7.1E-01	6.5E-01
**S4_R2**	2.3E+00	2.1E+00	1.7E+00	1.5E+00	1.4E+00	9.8E-01	8.1E-01	7.9E-01	1.2E+00	1.0E+00	8.8E-01	8.0E-01
**S4_R3**	2.7E+00	2.4E+00	2.0E+00	1.7E+00	1.6E+00	1.1E+00	9.2E-01	9.0E-01	1.4E+00	1.1E+00	1.0E+00	9.2E-01
**S4_R4**	2.9E+00	2.6E+00	2.2E+00	1.9E+00	1.7E+00	1.2E+00	1.0E+00	9.8E-01	1.5E+00	1.2E+00	1.1E+00	1.0E+00

Table S14: Bayes factors for *mad3*-GFP

mad3-GFP	S2_R1	S2_R2	S2_R3	S2_R4	S3_R1	S3_R2	S3_R3	S3_R4	S4_R1	S4_R2	S4_R3	S4_R4
**S2_R1**	1.0E+00	6.1E+01	6.5E+01	5.7E+01	8.3E+01	2.0E+01	2.1E+01	2.1E+01	2.7E+01	2.0E+01	2.0E+01	1.9E+01
**S2_R2**	1.6E-02	1.0E+00	1.1E+00	9.4E-01	1.4E+00	3.3E-01	3.4E-01	3.5E-01	4.4E-01	3.3E-01	3.3E-01	3.2E-01
**S2_R3**	1.5E-02	9.3E-01	1.0E+00	8.8E-01	1.3E+00	3.1E-01	3.2E-01	3.2E-01	4.1E-01	3.0E-01	3.1E-01	2.9E-01
**S2_R4**	1.7E-02	1.1E+00	1.1E+00	1.0E+00	1.5E+00	3.5E-01	3.6E-01	3.7E-01	4.7E-01	3.5E-01	3.5E-01	3.4E-01
**S3_R1**	1.2E-02	7.3E-01	7.9E-01	6.9E-01	1.0E+00	2.4E-01	2.5E-01	2.5E-01	3.2E-01	2.4E-01	2.4E-01	2.3E-01
**S3_R2**	5.0E-02	3.0E+00	3.3E+00	2.9E+00	4.2E+00	1.0E+00	1.0E+00	1.1E+00	1.3E+00	1.0E+00	1.0E+00	9.6E-01
**S3_R3**	4.8E-02	2.9E+00	3.1E+00	2.8E+00	4.0E+00	9.6E-01	1.0E+00	1.0E+00	1.3E+00	9.6E-01	9.7E-01	9.3E-01
**S3_R4**	4.7E-02	2.9E+00	3.1E+00	2.7E+00	3.9E+00	9.5E-01	9.9E-01	1.0E+00	1.3E+00	9.5E-01	9.6E-01	9.1E-01
**S4_R1**	3.7E-02	2.3E+00	2.4E+00	2.1E+00	3.1E+00	7.4E-01	7.7E-01	7.8E-01	1.0E+00	7.4E-01	7.5E-01	7.2E-01
**S4_R2**	5.0E-02	3.1E+00	3.3E+00	2.9E+00	4.2E+00	1.0E+00	1.0E+00	1.1E+00	1.4E+00	1.0E+00	1.0E+00	9.7E-01
**S4_R3**	4.9E-02	3.0E+00	3.2E+00	2.8E+00	4.1E+00	9.9E-01	1.0E+00	1.0E+00	1.3E+00	9.8E-01	1.0E+00	9.5E-01
**S4_R4**	5.2E-02	3.2E+00	3.4E+00	3.0E+00	4.3E+00	1.0E+00	1.1E+00	1.1E+00	1.4E+00	1.0E+00	1.1E+00	1.0E+00

Table S15: Bayes factors for *bub1*-GFP

bub1-GFP	S2_R1	S2_R2	S2_R3	S2_R4	S3_R1	S3_R2	S3_R3	S3_R4	S4_R1	S4_R2	S4_R3	S4_R4
**S2_R1**	1.0E+00	2.8E+00	1.3E+01	5.4E+01	1.5E+01	8.8E+02	1.5E+03	1.5E+03	3.9E+02	1.0E+03	2.1E+02	1.6E+02
**S2_R2**	3.6E-01	1.0E+00	4.7E+00	1.9E+01	5.4E+00	3.1E+02	5.2E+02	5.2E+02	1.4E+02	3.6E+02	7.3E+01	5.6E+01
**S2_R3**	7.7E-02	2.1E-01	1.0E+00	4.1E+00	1.2E+00	6.8E+01	1.1E+02	1.1E+02	3.0E+01	7.8E+01	1.6E+01	1.2E+01
**S2_R4**	1.9E-02	5.2E-02	2.4E-01	1.0E+00	2.8E-01	1.6E+01	2.7E+01	2.7E+01	7.2E+00	1.9E+01	3.8E+00	3.0E+00
**S3_R1**	6.6E-02	1.9E-01	8.6E-01	3.5E+00	1.0E+00	5.8E+01	9.6E+01	9.7E+01	2.6E+01	6.7E+01	1.4E+01	1.0E+01
**S3_R2**	1.1E-03	3.2E-03	1.5E-02	6.1E-02	1.7E-02	1.0E+00	1.7E+00	1.7E+00	4.4E-01	1.1E+00	2.3E-01	1.8E-01
**S3_R3**	6.9E-04	1.9E-03	9.0E-03	3.7E-02	1.0E-02	6.1E-01	1.0E+00	1.0E+00	2.7E-01	7.0E-01	1.4E-01	1.1E-01
**S3_R4**	6.8E-04	1.9E-03	6.5E-01	3.7E-02	1.0E-02	6.0E-01	1.0E+00	1.0E+00	2.6E-01	6.9E-01	1.4E-01	1.1E-01
**S4_R1**	2.6E-03	7.2E-03	3.4E-02	1.4E-01	3.9E-02	2.3E+00	3.8E+00	3.8E+00	1.0E+00	2.6E+00	5.3E-01	4.1E-01
**S4_R2**	9.9E-04	2.8E-03	1.3E-02	5.3E-02	1.5E-02	8.7E-01	1.4E+00	1.4E+00	3.8E-01	1.0E+00	2.0E-01	1.6E-01
**S4_R3**	4.9E-03	1.4E-02	6.4E-02	2.6E-01	7.4E-02	4.3E+00	7.1E+00	7.1E+00	1.9E+00	5.0E+00	1.0E+00	7.7E-01
**S4_R4**	6.3E-03	1.8E-02	8.2E-02	3.4E-01	9.6E-02	5.6E+00	7.0E+00	9.2E+00	2.4E+00	6.4E+00	1.3E+00	1.0E+00

Table S16: Bayes factors for *rpb1*

rpb1	S2_R1	S2_R2	S2_R3	S2_R4	S3_R1	S3_R2	S3_R3	S3_R4	S4_R1	S4_R2	S4_R3	S4_R4
**S2_R1**	1.0E+00	1.5E+02	9.5E+02	1.9E+03	8.4E+02	1.6E+03	1.1E+03	9.3E+02	1.9E+03	1.1E+03	1.0E+03	9.8E+02
**S2_R2**	6.6E-03	1.0E+00	6.3E+00	1.2E+01	5.5E+00	1.0E+01	7.3E+00	6.2E+00	1.3E+01	7.5E+00	6.8E+00	6.5E+00
**S2_R3**	1.1E-03	1.6E-01	1.0E+00	2.0E+00	8.8E-01	1.7E+00	1.2E+00	9.8E-01	2.0E+00	1.2E+00	1.1E+00	1.0E+00
**S2_R4**	5.3E-04	8.0E-02	5.0E-01	1.0E+00	4.5E-01	8.4E-01	5.9E-01	5.0E-01	1.0E+00	6.0E-01	5.4E-01	5.2E-01
**S3_R1**	1.2E-03	1.8E-01	1.1E+00	2.2E+00	1.0E+00	1.9E+00	1.3E+00	1.1E+00	2.3E+00	1.3E+00	1.2E+00	1.2E+00
**S3_R2**	6.3E-04	9.6E-02	6.0E-01	1.2E+00	5.3E-01	1.0E+00	7.1E-01	5.9E-01	1.2E+00	7.2E-01	6.5E-01	6.2E-01
**S3_R3**	9.0E-04	1.4E-01	8.5E-01	1.7E+00	7.5E-01	1.4E+00	1.0E+00	8.4E-01	1.7E+00	1.0E+00	9.2E-01	8.8E-01
**S3_R4**	1.1E-03	1.6E-01	1.0E+00	2.0E+00	9.0E-01	1.7E+00	1.2E+00	1.0E+00	2.1E+00	1.2E+00	1.1E+00	1.1E+00
**S4_R1**	5.2E-04	7.9E-02	4.9E-01	9.8E-01	4.4E-01	8.2E-01	5.8E-01	4.8E-01	1.0E+00	5.9E-01	5.3E-01	5.1E-01
**S4_R2**	8.8E-04	1.3E-01	8.4E-01	1.7E+00	7.4E-01	1.4E+00	9.8E-01	8.2E-01	1.7E+00	1.0E+00	9.0E-01	8.7E-01
**S4_R3**	9.8E-04	1.5E-01	9.3E-01	1.8E+00	8.2E-01	1.5E+00	1.1E+00	9.1E-01	1.9E+00	1.1E+00	1.0E+00	9.6E-01
**S4_R4**	1.0E-03	1.5E-01	9.7E-01	1.9E+00	8.6E-01	1.6E+00	1.1E+00	9.5E-01	2.0E+00	1.2E+00	1.0E+00	1.0E+00

Table S17: Bayes factors for *sep1*-GFP

sep1-GFP	S2_R1	S2_R2	S2_R3	S2_R4	S3_R1	S3_R2	S3_R3	S3_R4	S4_R1	S4_R2	S4_R3	S4_R4
**S2_R1**	1.0E+00	8.0E-01	7.4E-01	7.1E-01	9.9E-01	8.4E-01	7.7E-01	7.3E-01	1.0E+00	8.6E-01	8.1E-01	7.5E-01
**S2_R2**	1.2E+00	1.0E+00	9.2E-01	8.8E-01	1.2E+00	1.1E+00	9.6E-01	9.1E-01	1.3E+00	1.1E+00	1.0E+00	9.4E-01
**S2_R3**	1.4E+00	1.1E+00	1.0E+00	9.6E-01	1.3E+00	1.1E+00	1.0E+00	9.9E-01	1.4E+00	1.2E+00	1.1E+00	1.0E+00
**S2_R4**	1.4E+00	1.1E+00	1.0E+00	1.0E+00	1.4E+00	1.2E+00	1.1E+00	1.0E+00	1.4E+00	1.2E+00	1.1E+00	1.1E+00
**S3_R1**	1.0E+00	8.1E-01	7.4E-01	7.1E-01	1.0E+00	8.5E-01	7.8E-01	7.3E-01	1.0E+00	8.7E-01	8.2E-01	7.6E-01
**S3_R2**	1.2E+00	9.5E-01	8.8E-01	8.4E-01	1.2E+00	1.0E+00	9.1E-01	8.6E-01	1.2E+00	1.0E+00	9.6E-01	9.0E-01
**S3_R3**	1.3E+00	1.0E+00	9.6E-01	9.2E-01	1.3E+00	1.1E+00	1.0E+00	9.5E-01	1.3E+00	1.1E+00	1.1E+00	9.8E-01
**S3_R4**	1.4E+00	1.1E+00	1.0E+00	9.7E-01	1.4E+00	1.2E+00	1.1E+00	1.0E+00	1.4E+00	1.2E+00	1.1E+00	1.0E+00
**S4_R1**	9.9E-01	7.9E-01	7.3E-01	7.0E-01	9.8E-01	8.3E-01	7.6E-01	7.2E-01	1.0E+00	8.5E-01	8.0E-01	7.5E-01
**S4_R2**	1.2E+00	9.3E-01	8.6E-01	8.2E-01	1.1E+00	9.8E-01	8.9E-01	8.4E-01	1.2E+00	1.0E+00	9.4E-01	8.7E-01
**S4_R3**	1.2E+00	9.9E-01	9.1E-01	8.7E-01	1.2E+00	1.0E+00	9.5E-01	9.0E-01	1.2E+00	1.1E+00	1.0E+00	9.3E-01
**S4_R4**	1.3E+00	1.1E+00	9.8E-01	9.4E-01	1.3E+00	1.1E+00	1.0E+00	9.6E-01	1.3E+00	1.1E+00	1.1E+00	1.0E+00

Table S18: Summary of Bayesian model selection results

The symbol X denotes a model which can explain the data for a particular gene, i.e. all the 12 Bayes factors for this model are greater than or equal to 1/10. Those models that explain data from all genes are shaded in yellow.

	S2_R1	S2_R2	S2_R3	S2_R4	S3_R1	S3_R2	S3_R3	S3_R4	S4_R1	S4_R2	S4_R3	S4_R4
mad1							X	X		X	X	X
mad1-GFP						X	X	X	X	X		
mad2-GFP	X	X	X	X	X	X	X	X	X	X	X	X
mad3-GFP		X	X	X	X	X	X	X	X	X	X	X
bub1-GFP						X	X	X	X	X	X	X
rpb1			X	X	X	X	X	X	X	X	X	X
sep1-GFP	X	X	X	X	X	X	X	X	X	X	X	X

Furthermore, from the posterior distributions we can estimate the value of the parameters and quantify their uncertainty. For this purpose, we used the model $S = R = 3$ since it is one of the few models which can explain the data from all genes. In Table S19 we show the estimates of the three parameters and their uncertainty (mean and standard deviation of their posterior distributions), as well as two biologically meaningful functions of these parameters:

- T_{init}/T_{degr}, the ratio of the mean time between two successive transcription events and the mean time between two successive cytoplasmic degradation events, and
- T_{expt}/T_{init}, the ratio of the mean time for nuclear export and the mean time between two successive transcription events.

Computing percentage error as standard deviation divided by the mean, the percentage error is in the range 10-36 % for k_A, 4-69 % for k_B, 21-66 % for k_C, 1.4-3.3 % for T_{init}/T_{degr}, and 26-186 % for T_{expt}/T_{init}. Hence, the most clearly identifiable parameter is T_{init}/T_{degr}. The underlying reason is that this parameter is equal to the model's prediction of the inverse of the cytoplasmic mean which has been directly measured. The large errors in T_{expt}/T_{init} are to be expected because this quantity is equal to the model's prediction of the nuclear mean which we do not use in our objective function since the estimated mean from experimental data without transcription site labeled is substantially different from that estimated from data with transcription site labeled. Nevertheless, the mean nuclear mRNA per gene copy predicted for

mad1 and *rpb1* from non-TS labeled data are in good agreement with those measured in the TS-labeled data (Table S19), which is remarkable given that the TS-labeled data was not used to estimate parameters.

Note that the posterior distributions are not uniform (as the priors), but peaked (Fig. S4), implying that the inference procedure was successful at finding optimal parameter values for each gene. The identified parameters varied across genes (Fig. 8D, S4).

Table S19: Estimation of parameters from non-TS labeled data

Estimation uses the $S = R = 3$ model with $k_D = R$. In brackets we show the estimated uncertainty; this is computed using the standard deviation of the accepted ABC sample estimates. T_{expt}/T_{init} is equal to the model's prediction of the mean mature nuclear mRNA number. In the last column, for comparison, we show the mean mature nuclear mRNA number per gene copy from TS-labeled data (Fig. S18D). Note that the choice $k_D = R$ implies that k_A, k_B, k_C have units of inverse time where one time unit equals the mean time for one cytoplasmic mRNA molecule to decay.

Gene	$\boldsymbol{k_A}$	$\boldsymbol{k_B}$	$\boldsymbol{k_C}$	$\boldsymbol{T_{init}/T_{degr}}$	$\boldsymbol{T_{expt}/T_{init}}$	mean mature nuclear mRNA per gene copy
mad1	2.54 (0.504)	3.64 (1.79)	20.0 (9.08)	1.15 (0.0366)	0.0533 (0.0248)	0.064
mad1-GFP	3.49 (1.24)	20.4 (9.92)	111 (37.9)	0.7 (0.0106)	0.0145 (0.00559)	N/A
mad2-GFP	14.8 (3.46)	1.84 (0.125)	4.33 (2.33)	0.689 (0.0230)	0.453 (0.271)	N/A
mad3-GFP	13.3 (1.39)	2.42 (0.0997)	90.4 (31.5)	0.567 (0.00804)	0.0220 (0.00851)	N/A
bub1-GFP	5.62 (1.80)	26.9 (18.5)	45.5 (9.77)	0.455 (0.00732)	0.0510 (0.0133)	N/A
rpb1	49.2 (5.82)	14.3 (1.07)	27.0 (9.35)	0.112 (0.00157)	0.382 (0.164)	0.604
sep1-GFP	53.7 (19.1)	3.08 (0.179)	10.0 (6.61)	0.368 (0.00983)	0.636 (1.18)	N/A

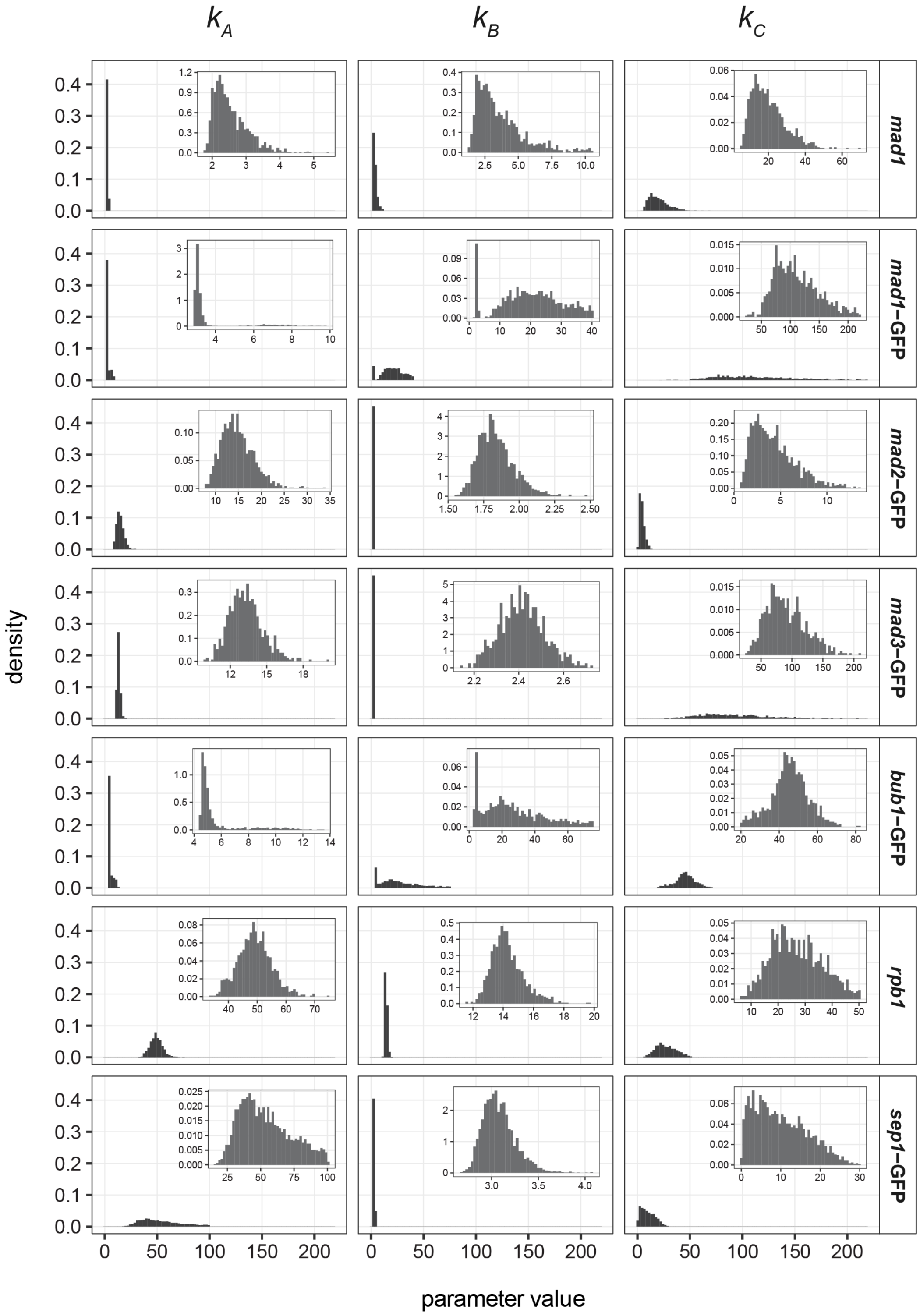

Figure S4: Posterior distributions of k_A, k_B, and k_C
Posterior distributions were obtained using an ABC rejection sampler with the model with $S = R = 3$. Main panels use the same axes, the insets use axes optimized for each parameter and gene.

B.6 Verifying the ABC results using a different objective function

In the previous section the inference results are based on the particular choice of the objective function. Here we test its validity more extensively by using a different type of objective function where we weigh the cytoplasmic mean, nuclear Fano factor and cytoplasmic Fano factor by the inverse of their sampling variance determined by bootstrapping. The new objective function is given by

$$d = \frac{1}{SV(\mu_c^e)}\left(\frac{2\mu_c - \mu_c^e}{\mu_c^e}\right)^2 + \frac{1}{SV(FF_c^e)}\left(\frac{FF_c - FF_c^e}{FF_c^e}\right)^2 + \frac{1}{SV(FF_N^e)}\left(\frac{FF_N - FF_N^e}{FF_N^e}\right)^2.$$

Here SV are the sampling variances of each quantity, which are cell size-corrected for the Fano factors. Note that if the sampling variances were all equal then the new objective function would equal the old one multiplied by some constant and hence the inference results would be the same. However because these variances are generally different for the means and the Fano factors, it is difficult to *a priori* say what effect this will have on the values of the parameters inferred. The ABC inference using the new metric was done for the case $S = R = 3$ using the Sequential Monte Carlo-Approximate Bayesian Computation (SMC-ABC) algorithm in the Julia package (*103*), which provided a substantial reduction in computation time. The posteriors and uncertainty plots for non-TS labeled data are shown in Fig. S5 and S6, and the key quantities are reported in Table S20. Posterior distributions are very similar to those obtained with the original objective function and ABC rejection sampler (Fig. S7), demonstrating that the inference results are not very sensitive to the choice of objective function. The small differences stem most likely from the fact that previously we used the ABC rejection sampler whereas in this case we used the SMC-ABC algorithm.

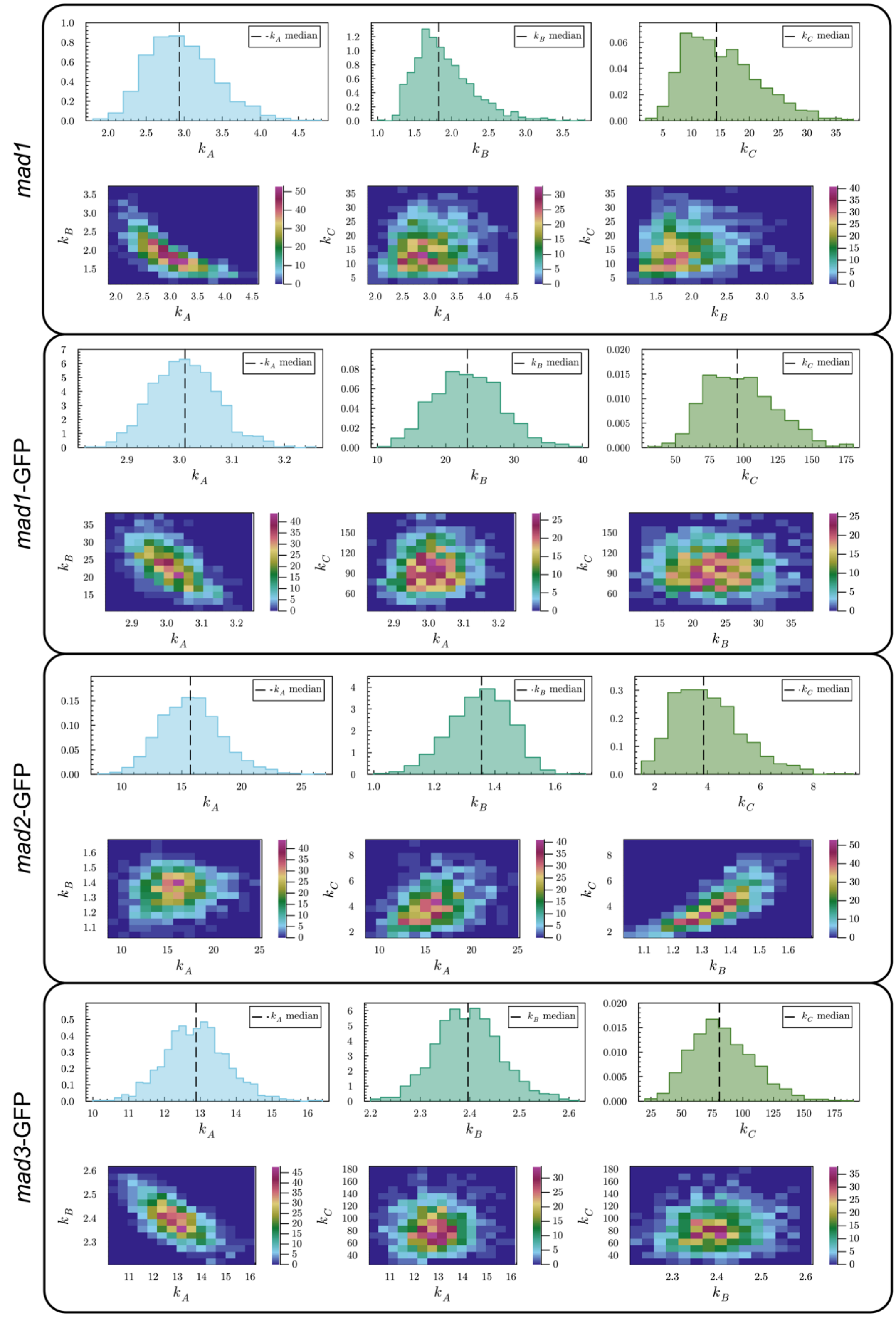

Figure S5: Posterior and uncertainty plots from the SMC-ABC calibration for *mad1*, *mad1*-GFP, *mad2*-GFP, and *mad3*-GFP

The sampling variance-weighted objective function was used with $S = R = 3$.

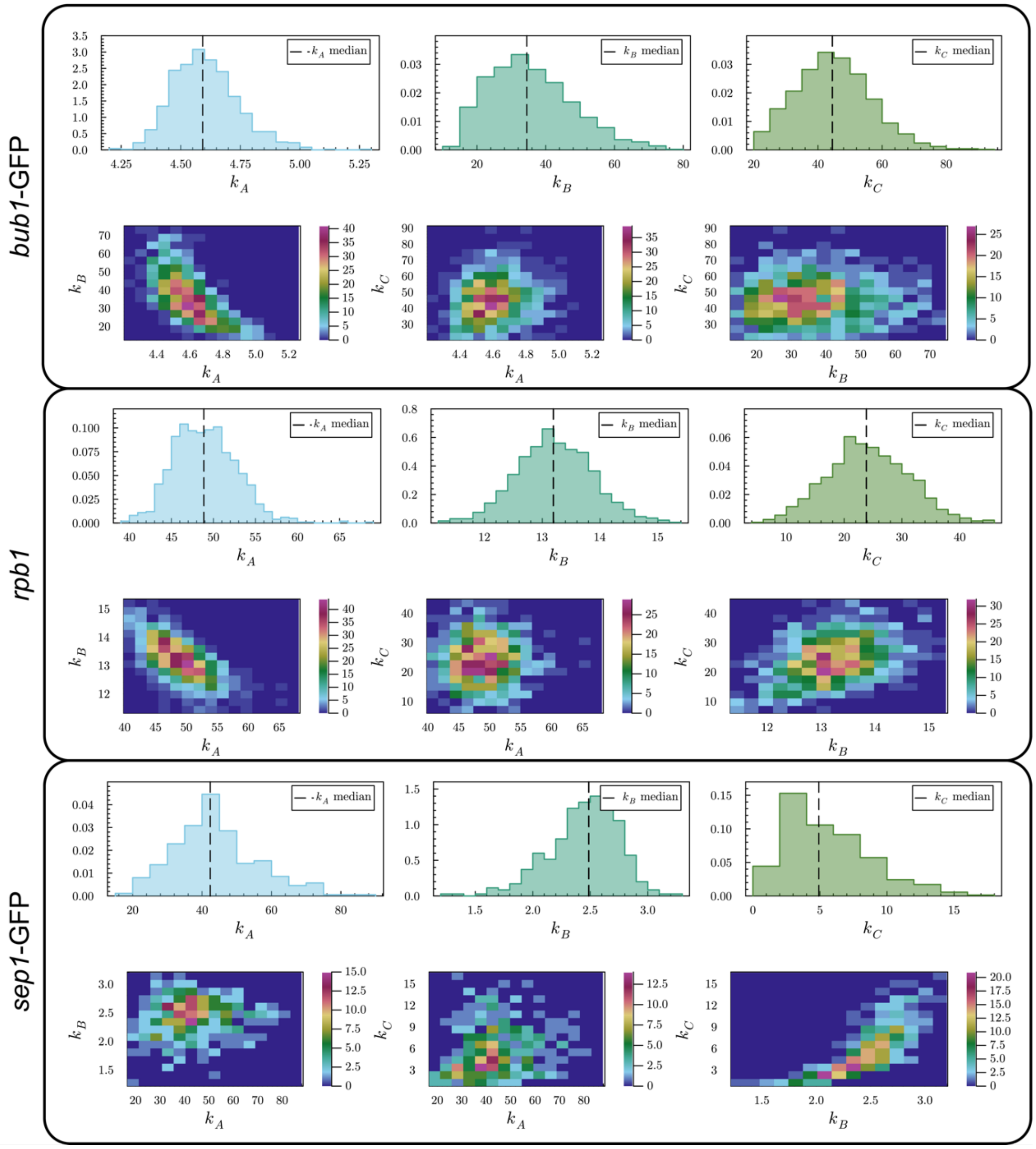

Figure S6: Posterior and uncertainty plots from the SMC-ABC calibration for *bub1*-GFP, *rpb1*, and *sep1*-GFP
The sampling variance-weighted objective function was used with $S = R = 3$.

Table S20: Results from the SMC-ABC model inference using the sampling variance-weighted objective function

Values of k_A, k_B, k_C are the average values from the posterior, with the standard deviations of the ABC posteriors in parentheses. Cor(x,y) is the correlation between the parameters x and y. The value d_c gives the final threshold value of the objective function from the schedule specified in the SMC-ABC inference.

Gene	k_A	k_B	k_C	$Cor(k_A, k_B)$	$Cor(k_A, k_C)$	$Cor(k_B, k_C)$	d_c
mad1	2.97 (0.444)	1.9 (0.39)	15.3 (6.31)	-0.779	0.139	0.163	5
mad1-GFP	3.01 (0.0619)	23.2 (4.85)	97.7 (25.0)	-0.637	0.113	0.00442	0.1
mad2-GFP	15.8 (2.55)	1.35 (0.0989)	4.03 (1.28)	0.113	0.372	0.793	5
mad3-GFP	12.9 (0.875)	2.4 (0.0656)	83.3 (25.5)	-0.702	0.0423	0.118	0.15
bub1-GFP	4.61 (0.14)	36 (12.2)	45 (11.7)	-0.694	0.159	0.0553	0.5
rpb1	49 (3.76)	13.2 (0.656)	24.1 (6.98)	-0.666	0.0265	0.401	0.2
sep1-GFP	43.7 (12.1)	2.44 (0.305)	5.6 (3.19)	0.0913	0.24	0.781	0.5

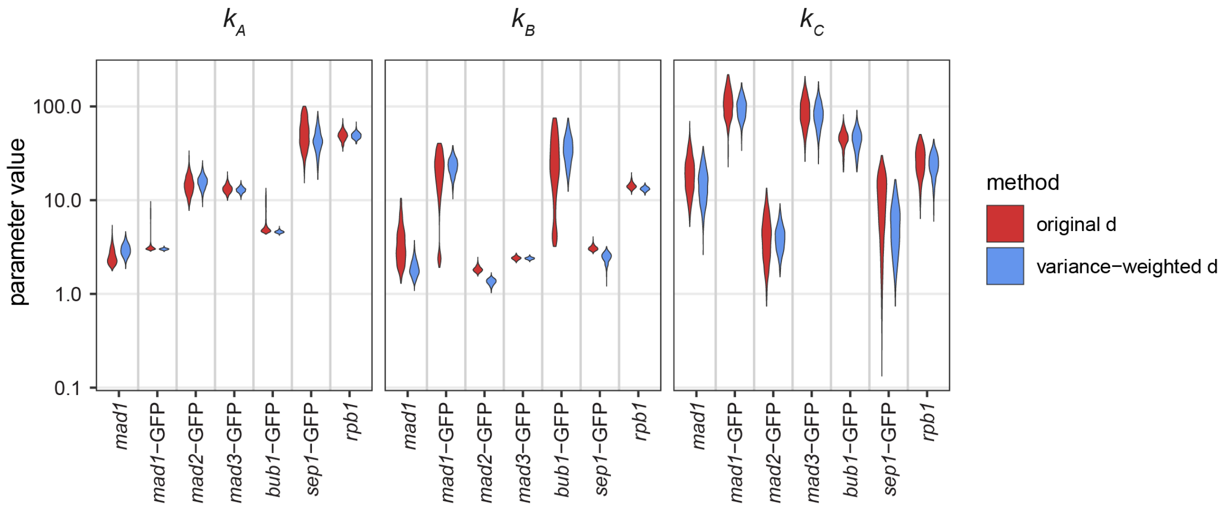

Figure S7: Comparison of model inference results from different methods

Comparison of posterior distributions obtained with the original objective function and ABC rejection sampler (original d) with those obtained with the sampling variance-weighted objective function and SMC-ABC method (variance-weighted d). In both cases, the model with $S = R = 3$ was used.

B.7 Testing the ansatz of Poisson fluctuations

We considered the possibility that—while the measurements indicate sub-Poissonian mRNA distributions—they may instead result from a Poisson process and appear sub-Poissonian due to finite sample sizes. Specifically, if n Poisson-distributed random variables are generated (mimicking the mRNA number produced with a single rate-limiting step and measured in n cells), and the Fano factor (variance / mean) is calculated for this population of cells, then the Fano factor will vary around 1 and has a 50 % chance of being less than 1. In fact, it is known that the sampling distribution of the Fano factor is approximately a gamma distribution with shape parameter $(n-1)/2$ and scale parameter $2/(n-1)$ (*104*). Hence if the data from n cells were generated by a Poisson process, with probability 0.95 the computed Fano factor would lie in the interval $[\lambda_1, \lambda_2]$ where $\int_0^{\lambda_1} \Gamma(x; (n-1)/2, 2/(n-1))dx = 0.025$ and $\int_0^{\lambda_2} \Gamma(x; (n-1)/2, 2/(n-1))dx = 0.975$. In Table S21 we show the computation of the 95 % confidence interval for the Fano factor for all Poisson and sub-Poisson genes in mononucleated cells (non-TS labeled data).

Table S21: Testing if the measured Fano factor could be due to finite sampling of Poisson noise
For each gene we compute the confidence interval (CI) for its Fano factor given the number of cells and assuming mRNA fluctuations are Poissonian. Fano factor values in red indicate those which fall within the confidence intervals.

Gene	Number of cells	Nuclear Fano factor	Cytoplasmic Fano factor	95 % CI for Fano factor
mad1	1382	0.929	0.620	[0.927,1.08]
mad1-GFP	2155	0.983	0.657	[0.941,1.06]
mad2-GFP	3064	0.845	0.824	[0.951,1.05]
mad3-GFP	2661	0.974	0.725	[0.947,1.05]
bub1-GFP	1142	0.951	0.621	[0.920,1.08]
rpb1	1425	0.826	0.647	[0.928,1.07]
sep1-GFP	589	0.865	0.880	[0.889,1.11]
cdc13	1144	0.929	0.997	[0.920,1.08]

Note that *cdc13* is the only gene in this list whose nuclear and cytoplasmic Fano factors both fall within the confidence intervals (also see Fig. S1). On this basis, we cannot rule out that this gene has Poissonian expression characteristics, and hence we have not used data from this gene when fitting to the model. We note that for some genes their nuclear Fano factor falls within the confidence intervals whereas their cytoplasmic Fano factor does not (Table S21). This might be expected due to the relationship between nuclear and cytoplasmic Fano factors when the nuclear export rate is high (Fig. 8C).

Figs. S8–S19

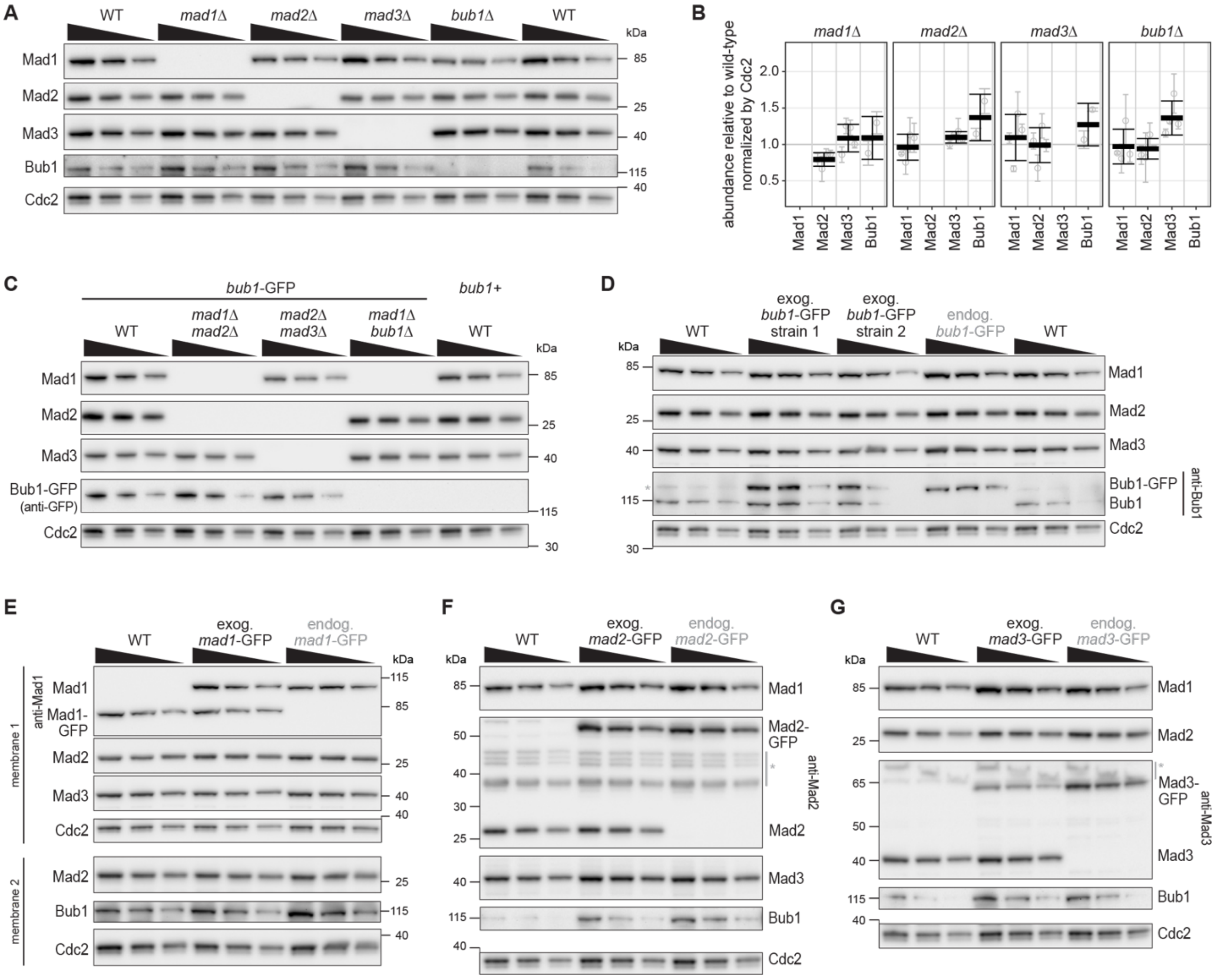

Fig. S8. Additional immunoblots for SAC gene deletions or overexpressions

(A, C-G) Immunoblot of cell extracts from the indicated strains; 75 % and 50 % of extract are loaded for the dilutions of each extract. Antibodies were against the endogenous proteins, except for (C), where an anti-GFP antibody was used to detect Bub1-GFP. Cdc2 serves as loading control. Extracts were from strains deleted for SAC genes (A), deleted for two SAC genes at a time (C), or additionally expressing the GFP-tagged version of a SAC gene from an exogenous locus (D-G). In (D-G), strains expressing GFP-tagged SAC genes from the endogenous locus are also shown for comparison. Gray asterisks indicate unspecific cross-reactions of the antibodies.

(B) Quantification of immunoblots from strains with single SAC genes deleted. The level of each protein was determined relative to the wild-type, and all values were normalized for the concentrations observed with the loading control anti-Cdc2 antibody. Gray data are mean and standard deviation from technical replicates in single experiments. Black data are mean and standard deviation across experimental replicates. Error from the linear regressions is not included, and error is not propagated, so that the error bars shown are likely an underestimate.

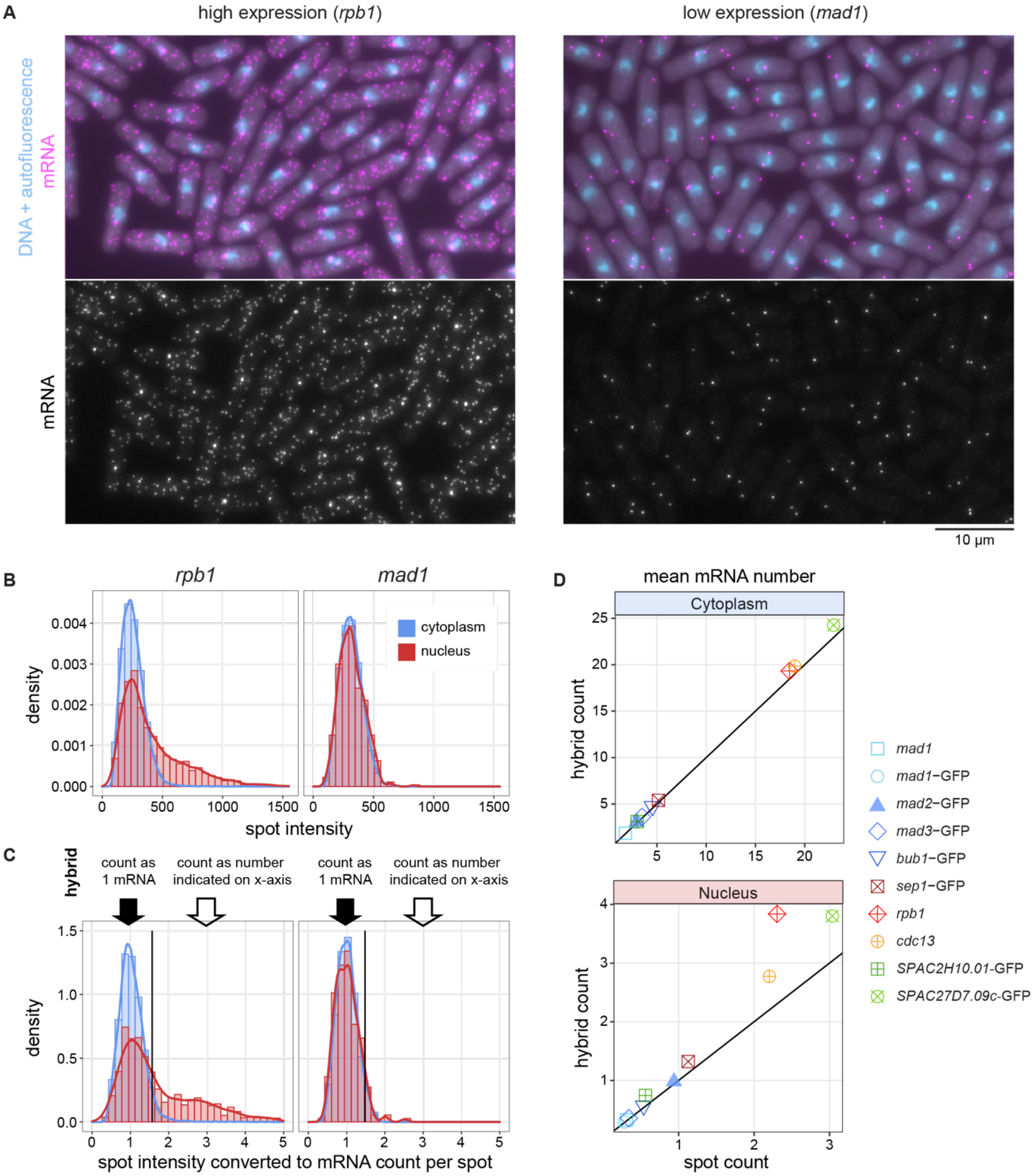

Fig. S9. Comparison between "spot count" and "hybrid count" to determine mRNA number per cell

(A) Representative images from smFISH experiments; DNA is stained with DAPI. The highly expressed gene (*rpb1*) shows higher intensity mRNA FISH spots in the nucleus, presumably representing transcription sites.

(B) Histogram and density distribution of FISH spot intensity ("AMP" from FISHquant) for *rpb1* and *mad1*. Higher intensity spots are almost exclusively found for *rpb1* and in nuclei. Pooled data from three independent experiments; *n* = 34,884 spots for *rpb1*; 3,424 spots for *mad1*.

(C) Single experiments from the data in (B) illustrating the "hybrid count" method. Data were normalized to the median of the spot intensity in the cytoplasm for each image. The vertical black line indicates the 95th percentile of intensities observed in the cytoplasm in this experiment and is used as cut-off between the count methods. *n* = 13,657 spots for *rpb1*; 1,511 spots for *mad1*.

(D) Comparison of mean mRNA counts per compartment obtained with the "spot count" method (which ignores spot intensities) and the "hybrid count" method illustrated in (C). Deviations are mostly observed for highly expressed genes and are most pronounced in the nucleus, as expected.

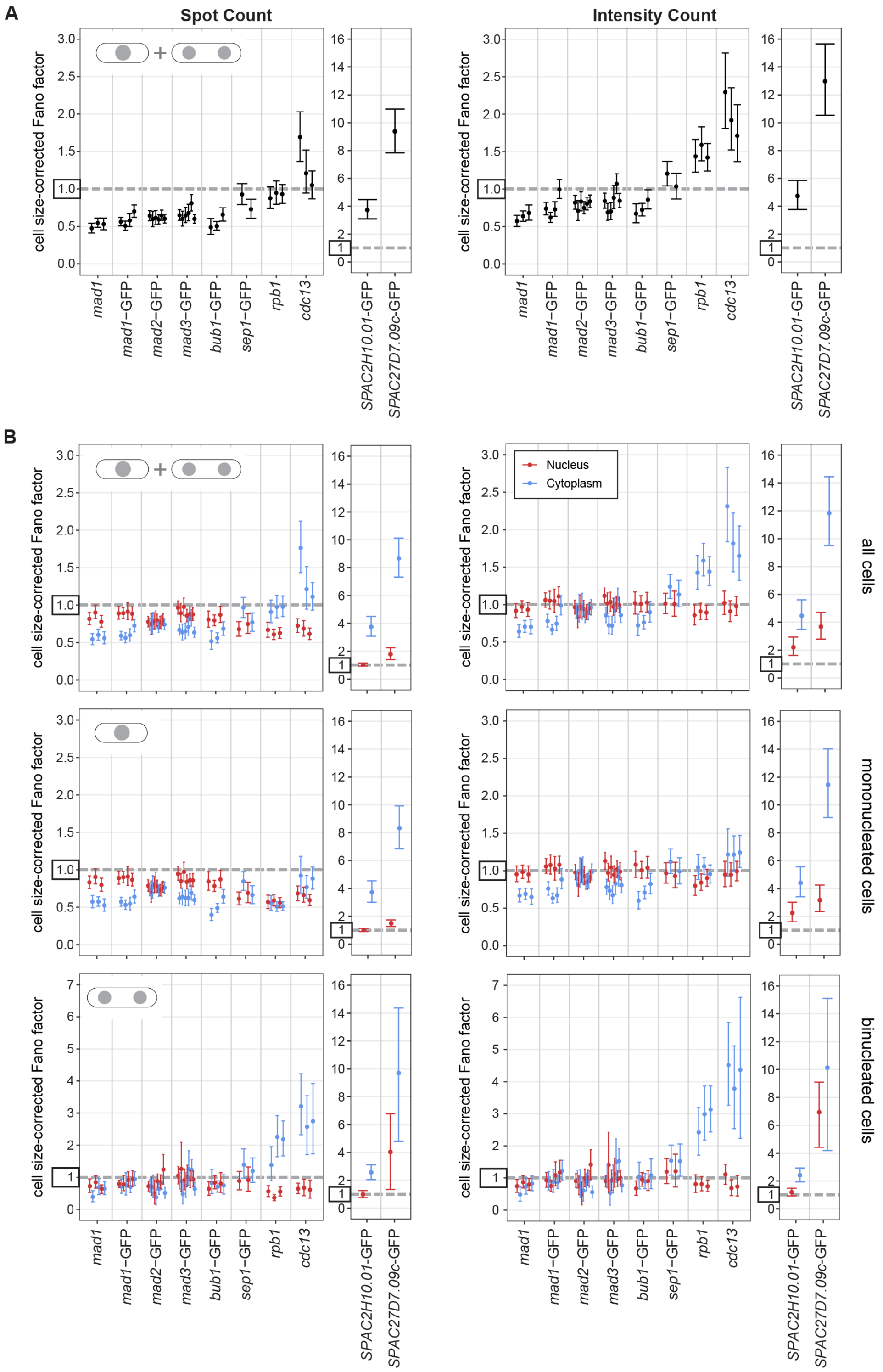

Fig. S10. Sub-Poissonian mRNA distributions are observed when different methods are used to count mRNA molecules

(A) Cell size-corrected Fano factors and their 95 % confidence intervals from counts of mRNA molecules per cell calculated using either the "spot count" (left) or "intensity count" (right) methods. Same experiments as in Fig. 2 and 3.

(B) Same as in (A) except Fano factors calculated separately for mRNA in the nucleus and cytoplasm. Fano factors calculated for all cells combined, or separately for mononucleated and binucleated cells. Same experiments as in Fig. 6.

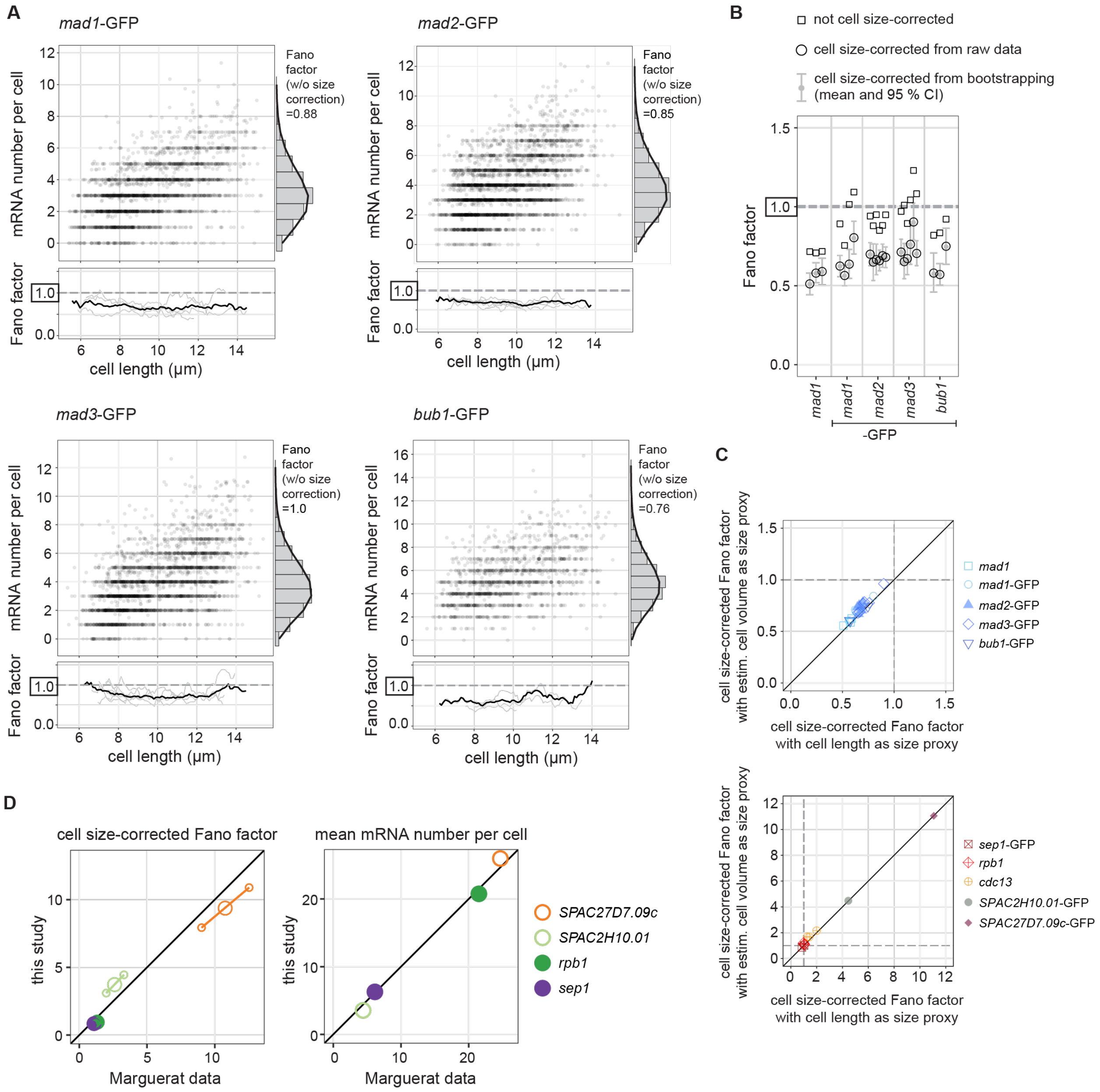

Fig. S11. Additional data for sub-Poissonian mRNA distributions of SAC genes

(A) Scatter plots of cell length versus mRNA number per cell for *mad1*-GFP (*n* = 2,499 cells), *mad2*-GFP (*n* = 3,501 cells), *mad3*-GFP (*n* = 2,993 cells), and *bub1*-GFP (*n* = 1,358 cells), all expressed from their endogenous locus. Data from 3–6 replicates combined. Right: Histogram of mRNA number across all cells with fit to Poisson distribution. Bottom: The Fano factor was determined in a sliding window spanning 1 µm of cell length. The Fano factors for single replicates are shown in light gray; the Fano factor for the pooled data in black. Data for untagged *mad1* is shown in Fig. 2.

(B) Comparison between uncorrected and cell size-corrected Fano factors for SAC genes. The cell size-corrected Fano factors from bootstrapping (also shown in Fig. 2) and their 95 % confidence intervals are shown in gray. 3–6 independent experiments for each gene are shown.

(C) Comparison of cell size-corrected Fano factors determined by either using cell length or cell volume as proxy for cell size. Cell volume is calculated from cell length, cell width, and the idealized assumption that an *S. pombe* cell is a cylinder with half-spheres at each end. Fano factors tend to be slightly higher when estimated cell volume is used, but they remain below 1 for SAC genes.

(D) Direct comparison between values determined by the Marguerat group and in this study. Left: cell size-corrected Fano factors with 95 % confidence intervals; right: mean mRNA numbers per cell. All data available for a single gene in each study were pooled. Note that *SPAC27D7.09c*, *SPAC2H10.01*, and *sep1* were tagged with GFP in this but not in the Marguerat studies. For our data the "spot count" for mRNA number was used for better comparability.

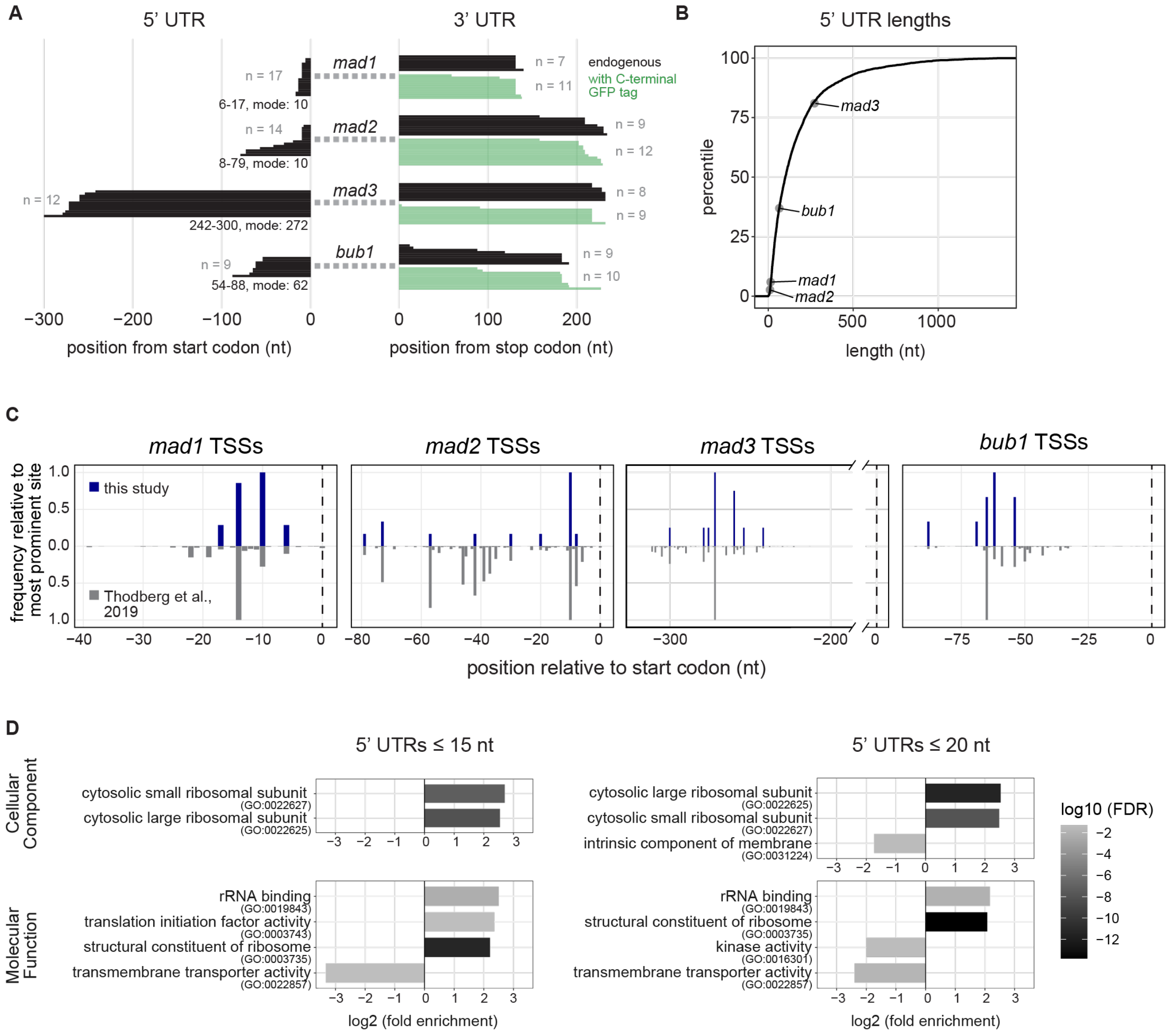

Fig. S12. Mapping of SAC gene UTRs

(A) Lengths of 5’ and 3’ untranslated regions (UTRs) determined by RACE-PCR; black, endogenous gene; green, ymEGFP-tagged gene. For the 5‘ UTRs, the spread in lengths and the most frequent length is given at the bottom.

(B) Lengths of SAC gene 5’ UTRs in comparison to genome-wide data from Thodberg *et al.* (*49*), displayed as empirical cumulative distribution (4,710 transcripts).

(C) Comparison of transcription start sites (TSSs) determined in this study by RACE-PCR, and determined in Thodberg *et al.* (*49*), by cap analysis of gene expression (CAGE). Data are normalized by setting the most prominent TSS for each gene to 1.

(D) Gene Ontology (GO) enrichment analysis for genes with short 5’ UTRs, as identified by Thodberg *et al.* (*49*). Genes with 5’ UTRs of 15 nucleotides or shorter (6.4th percentile), and 20 nucleotides or shorter (10th percentile) were analyzed. Ribosomal protein-coding genes are most prominently overrepresented. Value from Fisher’s exact test with FDR correction is shown.

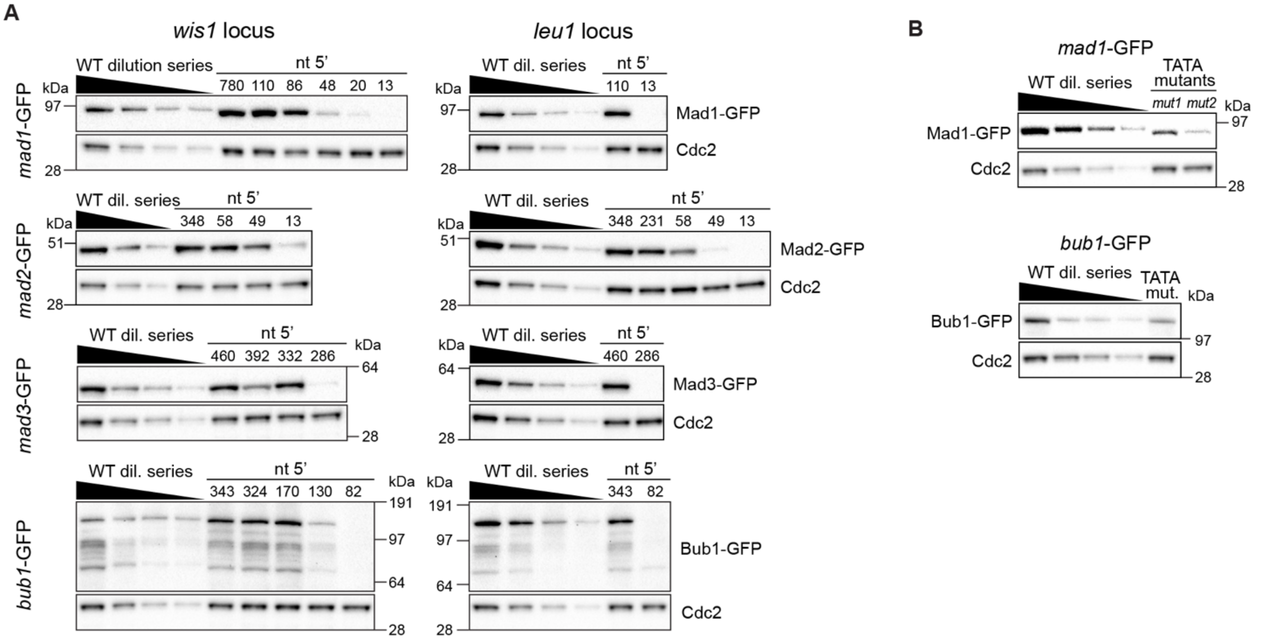

Fig. S13. Additional data on SAC gene promoter mapping

(A) Immunoblots of protein extracts from cells with either the endogenous gene tagged with GFP (WT) or a genome fragment containing the GFP-tagged gene inserted at an exogenous locus (*wis1* or *leu1*). The exogenous locus fragments for each gene contained a fixed sequence length 3' of the stop codon, but a variable number of nucleotides 5' of the start codon (nt 5'). A 1:1 serial dilution is loaded for WT extract. Quantification of these immunoblots is shown in Fig 4B.

(B) Examples of immunoblots quantified in Fig. 4E (one of two replicate experiments for each gene). Protein extracts from cells with either *mad1*-GFP or *bub1*-GFP inserted at the *leu1* locus. The *mad1* or *bub1* promoter was either left intact (WT) or was mutated at the site of the proposed TATA box. A 1:1 serial dilution was loaded for the extract from cells with WT promoter.

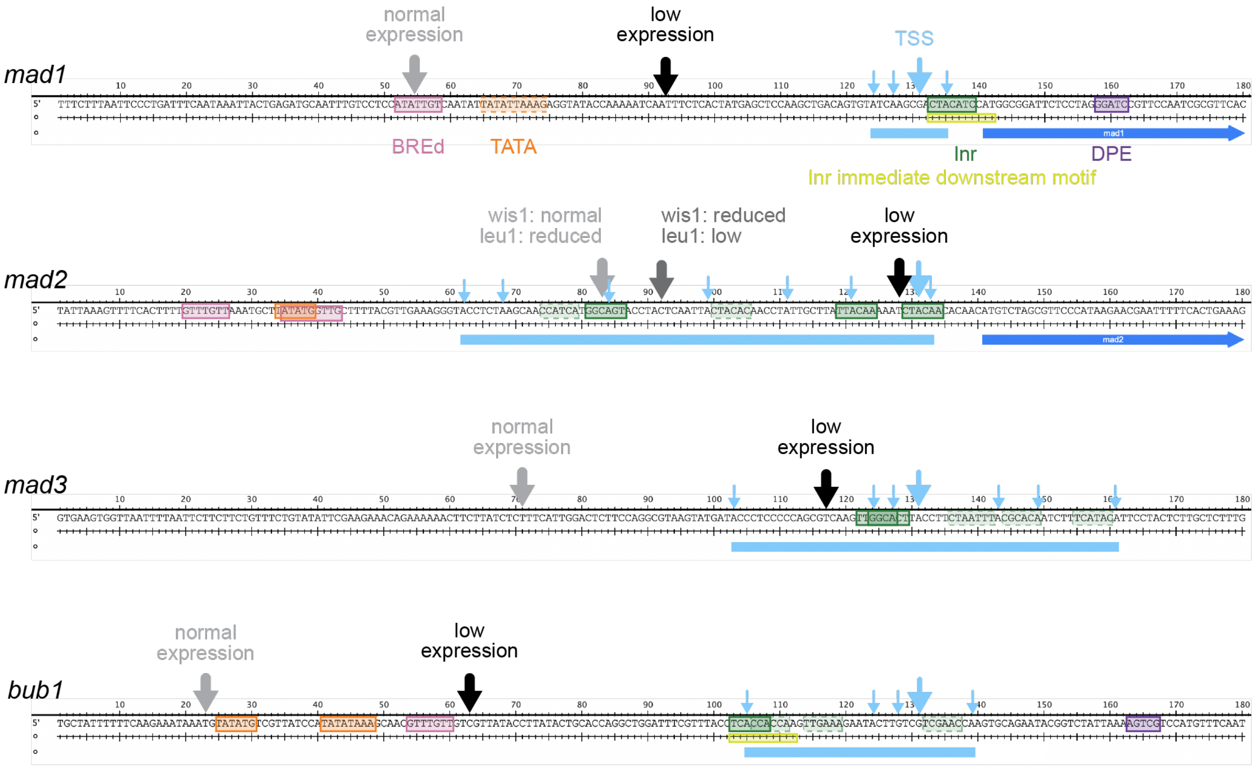

Fig. S14. Potential core promoter motifs of *S. pombe* SAC genes

Sequence around the transcription start site (TSS) of SAC genes with potential core promoter motifs annotated. The range of **TSSs** observed is indicated by a **light blue** bar, individual TSSs by light blue arrows, the most frequent TSS by the larger arrow. Coding sequences for *mad1* and *mad2* are indicated in blue. Arrows in gray or black point to the 5' ends of fragments that were or were not sufficient for expression (see Fig. 4). **TATA-box (orange)** identification used the consensus TATAWR (*105*). The region marked as dashed in *mad1* has a mismatch to that consensus, but was included because it resembles the TATAWAWR consensus (*106*), and because we found experimental evidence for its importance (Fig. 4). **BREd (pink)** identification used the RTDKKKK consensus (*105–107*). Motifs within 10 nucleotides of a TATA box are shown, even when they were located upstream. BREu sequences (consensus SSRRGCC) were not found. **Inr sequences (green)** were annotated when located in the TSS region and matching one of the consensus sequences YYRNMM (identified in *S. pombe* (*56*)), YYANWYY (*108*), or BBCABW (*106*). Dark green boxes have an observed TSS in the correct position of the Inr, light green boxes do not. Sequences resembling an **"Inr immediate downstream motif" (yellow)**, previously identified in *S. pombe* (*56*), were found in *mad1* and *bub1*, but not with the expected spacing relative to a TSS. **DPE (purple)** identification used the consensus RGWYV (*105, 107*).

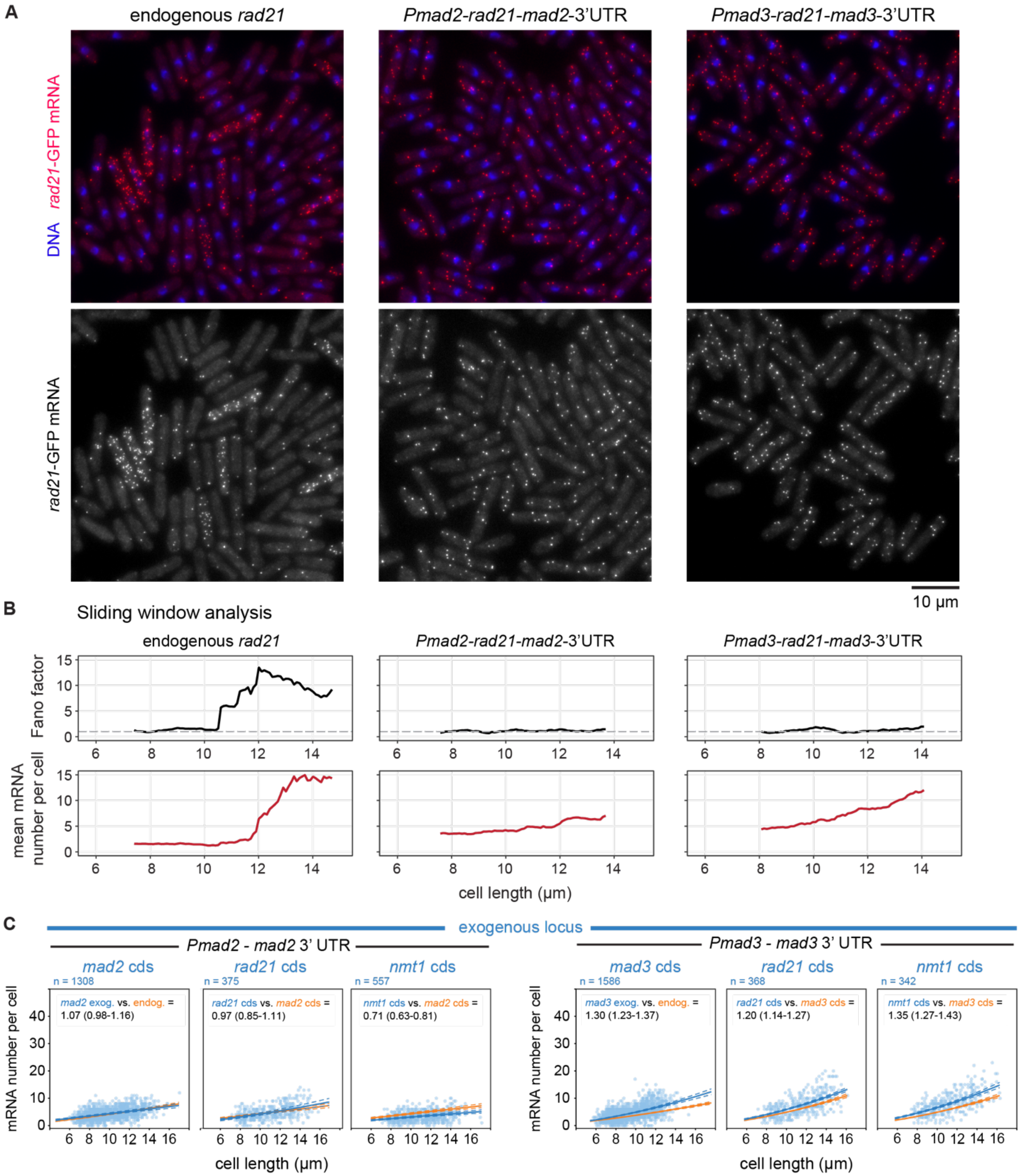

Fig. S15. Expression of *rad21* from *mad2* or *mad3* regulatory sequences reduces expression level and Fano factor

(A) Example images of *rad21*-GFP mRNA staining by smFISH (red). DNA is shown in blue (stained by DAPI). Expression from the endogenous *rad21* locus (left), or from the *wis1* locus with *mad2* regulatory sequences (middle) or *mad3* regulatory sequences (right).

(B) Same experiment as in (A). The Fano factor (black) and the mean mRNA number per cell (red) was determined in a sliding window spanning 1 μm of cell length. Only cell lengths for which more than 35 cells were available for quantification are shown.

(C) Same experiment as in Fig. 5B, except that "spot count", not "hybrid count" is shown. mRNA number relative to cell length at the exogenous locus for expression from the *mad2* promoter (*Pmad2*) and *mad2* downstream region or *mad3* promoter (*Pmad3*) and *mad3* downstream region. Solid blue lines are regression curves from generalized linear mixed model fits for the data shown, solid orange lines are regression curves for the reference data; dashed lines indicate the 95 % bootstrap confidence bands for the regression curves. Left panel on each side: comparing *mad2* or *mad3* expression at the exogenous locus to the endogenous locus; middle and right panel on each side: comparing *rad21* or *nmt1* cds at the exogenous locus to *mad2* or *mad3* cds at the exogenous locus. Model estimates of the ratio with bootstrap 95 % confidence interval in brackets are shown on top.

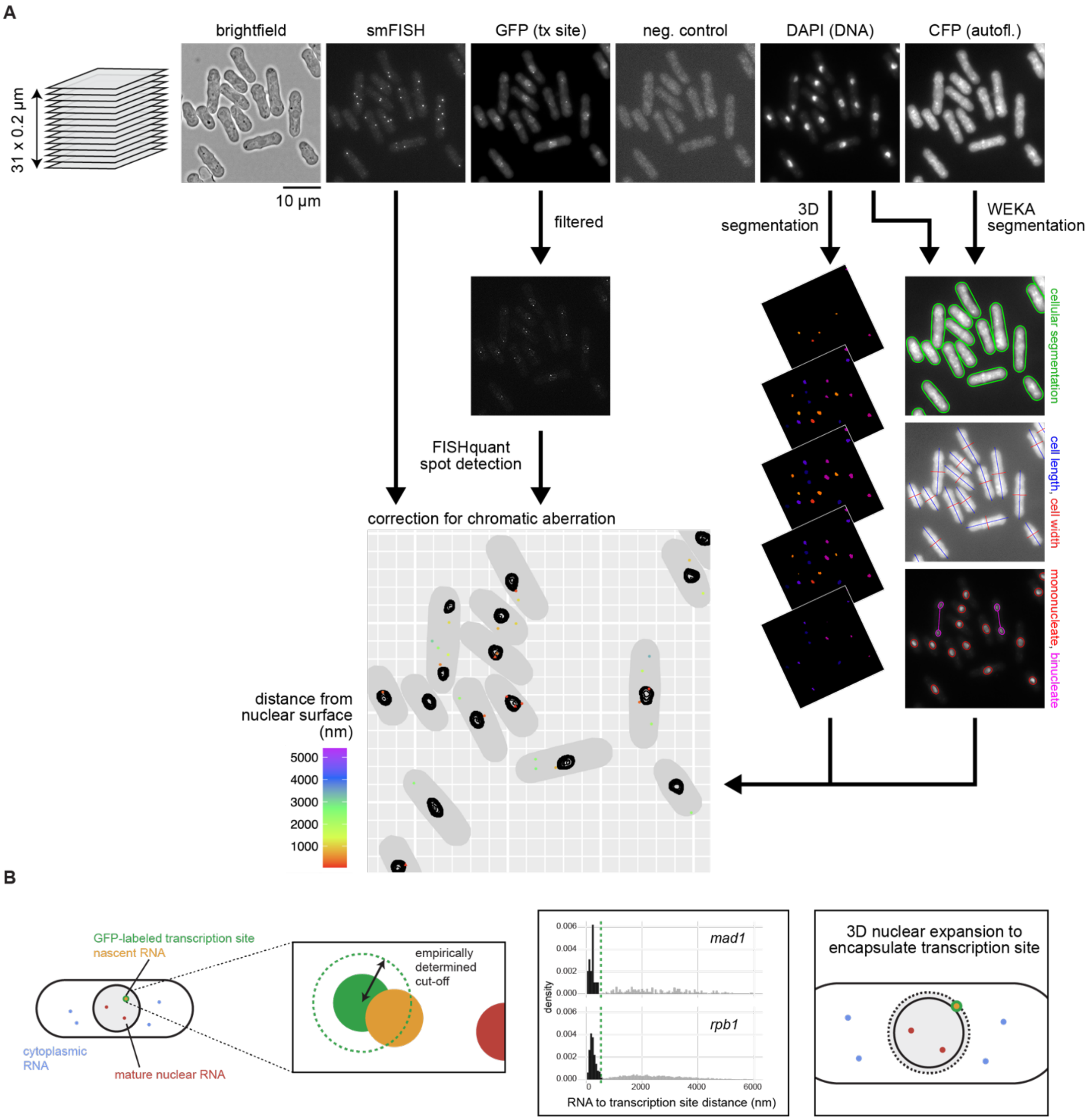

Fig. S16. Segmentation and image processing steps to distinguish nascent, nuclear, and cytoplasmic mRNA

(A) Image analysis pipeline. 31 Z-sections, spaced by 0.2 µm, were recorded in six channels. Sub-resolution fluorescent beads were imaged using the same settings (not shown), and this information was used to correct for chromatic aberration. Autofluorescence recorded with filters for cyan fluorescent protein (CFP) was used to segment cells in 2D by trainable WEKA segmentation. In addition, nuclei were segmented in 3D based on staining DNA with DAPI. Single-molecule mRNA FISH spots and GFP-labeled transcription sites were identified using FISHquant. After correcting for chromatic aberration, FISH spots were classified as nuclear if located within the 3D-segmented nucleus, and as cytoplasmic otherwise.

(B) FISH spots showed a bimodal distribution for distance to transcription sites, and this information was used as a cut-off to distinguish nascent mRNA from mature nuclear or cytoplasmic mRNA. In around 16 % of the cells, the transcription site was located just outside of the segmented nucleus. In these cases, the nuclear border was expanded to incorporate the transcription site. In an alternative approach, we excluded these cells from the analysis, which did not change the conclusions (see Fig. 7D vs. S18B).

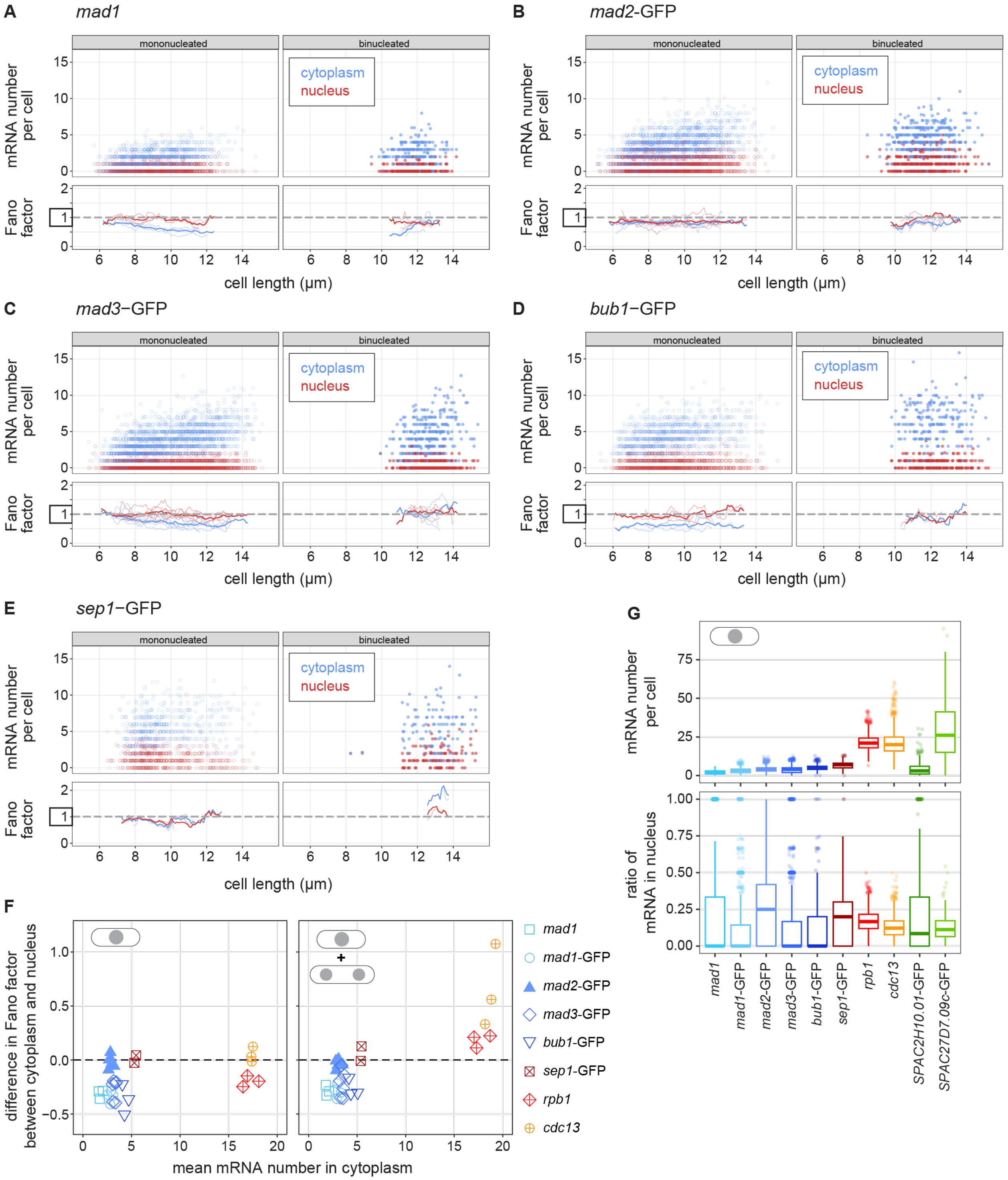

Fig. S17. Additional data on Fano factors of the nuclear and cytoplasmic mRNA distributions

(A-E) Top: Scatter plot of cell length versus mRNA number in cytoplasm (blue) or nucleus (red). Mono- and binucleated cells are shown separately. Bottom: The Fano factor was determined in a sliding window spanning 1 µm of cell length. The Fano factors for single replicates are shown as thin lines; the Fano factor for the pooled data as a thick line. See Fig. 6 for other genes.

(F) For each experiment, the mean mRNA number in the cytoplasm was calculated for cells with lengths in the interquartile range of all experiments (8.1–11.3 µm for all cells, 8.0–10.6 µm for mononucleated cells). Mean mRNA number is shown in relation to the difference between the Fano factor in the cytoplasm and the nucleus (data from Fig. 6A,F).

(G) Number of mRNA molecules in mononucleated cells (top) and fraction of these mRNAs in the nucleus (bottom); number of cells per gene between 212 and 3,064. Boxplots show median and interquartile range; whiskers extend to values no further than 1.5 times the interquartile range from the first and third quartile, respectively.

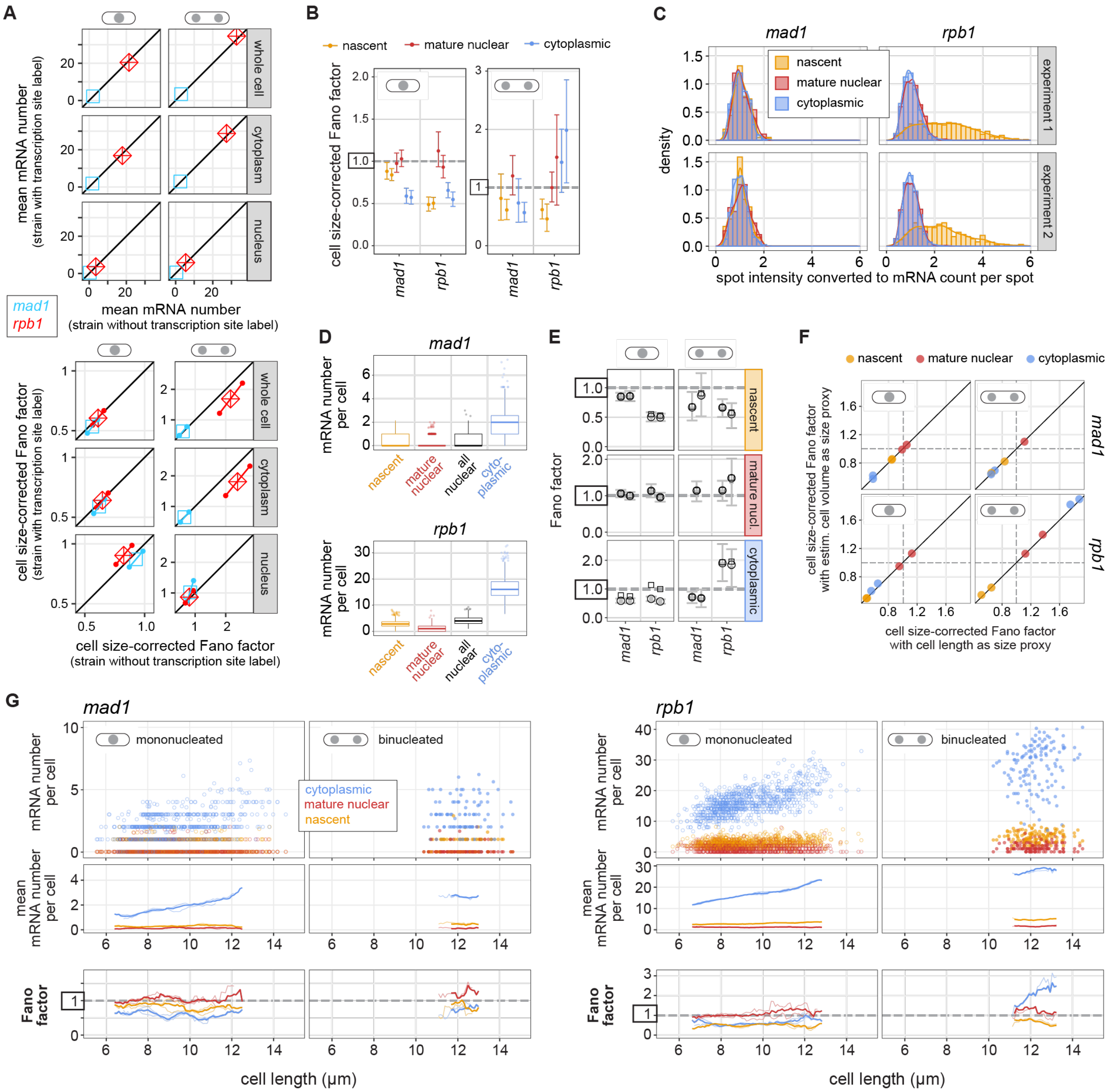

Fig. S18. Additional data from cells with transcription site labeled

(A) Comparison of mean mRNA number (top) and cell size-corrected Fano factor (with 95 % confidence interval) (bottom) for *mad1* and *rpb1* measured in cells without transcription site label (x-axis) or with transcription site label (y-axis).

(B) Same analysis as in Fig. 7D, except that cells in which the GFP-labeled transcription start site was not within the segmented nucleus were excluded. This removed between 11 % (*mad1*, mononucleated) and 36 % (*rpb1*, binucleated) of cells, yet yielded highly similar results.

(C) Histogram and density distribution of FISH spot intensity at the transcription site (nascent), at other positions in the nucleus (mature nuclear), or in the cytoplasm. Data were normalized to the median of the spot intensity in the cytoplasm for each image (n = 1,411 and 806 spots for *mad1*; n = 11,244 and 9,496 spots for *rpb1*). Spots identified as nascent by their proximity to the GFP-labeled *rpb1* gene contain a higher number of mRNAs, which (i) reflects strong

transcription of *rpb1*, and (ii) suggests that the identification of transcription sites by proximity to GFP is accurate.

(D) Numbers of mRNA in the different compartments in mononucleated cells; n = 884 cells for *mad1*, n = 921 cells for *rpb1*. Boxplots show median and interquartile range; whiskers extend to values no further than 1.5 times the interquartile range from the first and third quartile, respectively.

(E) Comparison between uncorrected (black square) and cell size-corrected (black circle) Fano factors for *mad1* and *rpb1* in different compartments. The cell size-corrected Fano factors from bootstrapping and their 95 % confidence interval (also shown in Fig. 7) are shown in gray; two independent experiments for each gene.

(F) Comparison of cell size-corrected Fano factors determined by using cell length or cell volume as proxy for cell size. Cell volume is calculated from cell length, cell width, and the idealized assumption that an *S. pombe* cell is a cylinder with half-spheres at each end. Individual experiments are shown as dots.

(G) Top: Scatter plot of cell length versus mRNA number at the transcription site (nascent, orange), in the nucleus, but not associated with the transcription site (mature nuclear, red), or in the cytoplasm (blue). Mono- and binucleated cells are shown separately. Middle: The mean mRNA number in the three different compartments was determined in a sliding window spanning 1 µm of cell length. Bottom: The Fano factor in the three different compartments was determined in a sliding window spanning 1 µm of cell length. For the sliding window data, single replicates are shown as thin lines; pooled data as a thick line.

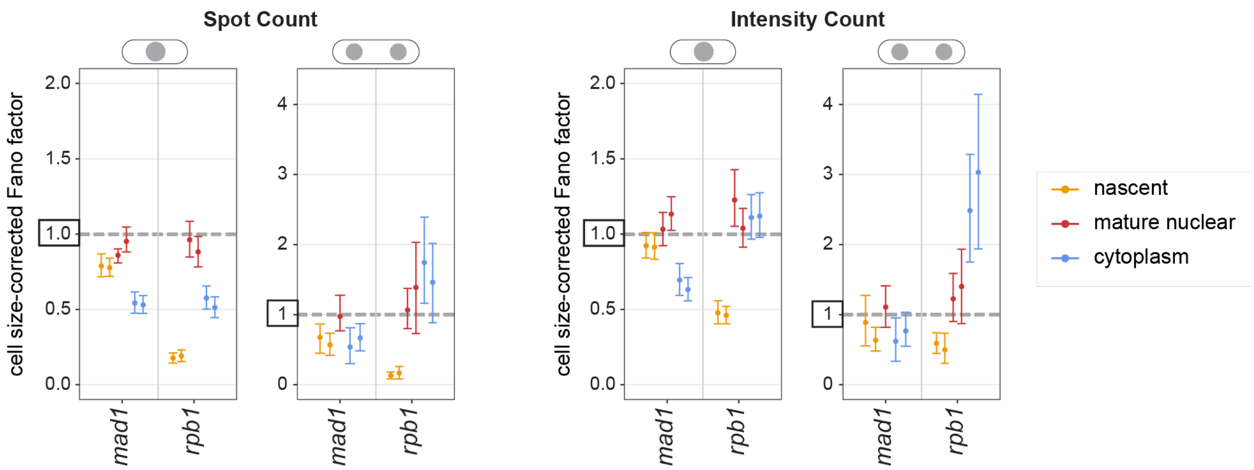

Fig. S19. Fano factors from cells with transcription site labeled when different methods are used to count mRNA molecules

Cell size-corrected Fano factors and their 95 % confidence intervals from counts of mRNA molecules per cell calculated using either the “spot count” (left) or “intensity count” (right) methods. Fano factors were calculated separately for mRNA at the transcription site (nascent), at other positions in the nucleus (mature nuclear), or in the cytoplasm. Same experiments as in Fig. 7.

Tables S22–S25

Table S22. *S. pombe* strains

Strain Number	Mating type	Genotype
JY001	*h-*	
JY002	*h+*	
JY265	*h-*	*leu1*
JY743	*h-*	*leu1 ura4-D18*
SU161	*h-*	*leu1<<343nt-bub1+-ymeGFP-427nt*
SU162	*h-*	*leu1<<82nt-bub1+-ymeGFP-427nt*
SU163	*h-*	*leu1<<110nt-mad1+-ymeGFP-164nt*
SU165	*h-*	*leu1<<13nt-mad1+-ymeGFP-164nt*
SU167	*h+*	*leu1<<49nt-mad2+-ymeGFP-521nt*
SU169	*h-*	*leu1<<460nt-mad3+-ymeGFP-279nt*
SU170	*h-*	*leu1<<286nt-mad3+-ymeGFP-279nt*
SU171	*h+*	*wis1+<<86nt-mad1+-ymEGFP-164nt*
SU172	*h+*	*wis1+<<48nt-mad1+-ymEGFP-164nt*
SU173	*h+*	*wis1+<<20nt-mad1+-ymEGFP-164nt*
SU174	*h+*	*wis1+<<13nt-mad1+-ymEGFP-164nt*
SU184	*h-*	*wis1+<<286nt-mad3+-ymEGFP-279nt*
SU185	*h+*	*wis1+<<130nt-bub1+-ymEGFP-427nt*
SU186	*h+*	*mad3Δ::ymEGFP*
SU186'	*h+*	*mad3Δ::ymEGFP*
SU189	*h+*	*wis1+<<170nt-bub1+-ymEGFP-427nt*
SU190	*h-*	*rad21+-ymEGFP<<kanR*
SU192	*h+*	*wis1+<<392nt-mad3+-ymEGFP-279nt*
SU193	*h+*	*wis1+<<460nt-mad3+-ymEGFP-279nt*
SU194	*h+*	*wis1+<<332nt-mad3+-ymEGFP-279nt*
SU194'	*h+*	*wis1+<<332nt-mad3+-ymEGFP-279nt*
SU216	*h-*	*leu1 ura4-D18 mad2+-ymEGFP*
SU228	*h+*	*bub1+-ymEGFP*
SU229	*h-*	*bub1+-ymEGFP*
SU229'	*h-*	*bub1+-ymEGFP*
SU230	*h+*	*bub1Δ::ymEGFP*
SU231	*h-*	*bub1Δ::ymEGFP*
SU499	*h-*	*leu1 ura4-D18 mad1+-ymEGFP*
SU804	*h-*	*leu1 ura4-D18 mad3+-ymEGFP*
SW130	*h+*	*mad2+-ymEGFP*
SW130'	*h+*	*mad2+-ymEGFP*
SW132	*h+*	*mad3+-ymEGFP*
SW132'	*h+*	*mad3+-ymEGFP*
SW139'	*h-*	*mad2Δ::ymEGFP*
SW140'	*h+*	*mad2Δ::ymEGFP*
SW176	*h+*	*mad1+-ymEGFP mad3+-ymEGFP*
SW201	*h-*	*mad1Δ::ymEGFP*
SW203	*h-*	*mad1Δ::ymEGFP*
SW204	*h+*	*mad1Δ::ymEGFP*
SW205	*h+*	*mad1+-ymEGFP*
SW206	*h+*	*mad1+-ymEGFP*
SW222	*h+*	*wis1+<<13nt-mad2+-ymEGFP-521nt*
SW224	*h+*	*wis1+<<49nt-mad2+-ymEGFP-521nt*
SW225	*h+*	*wis1+<<348nt-mad2+-ymEGFP-521nt*
SW226	*h+*	*wis1+<<58nt-mad2+-ymEGFP-521nt*
SW226'	*h+*	*wis1+<<58nt-mad2+-ymEGFP-521nt*
SW228	*h+*	*wis1+<<780nt-mad1+-ymEGFP-164nt*
SW229	*h+*	*wis1+<<343nt-bub1+-ymEGFP-427nt*
SW231	*h+*	*wis1+<<82nt-bub1+-ymEGFP-427nt*
SW232	*h+*	*wis1+<<324nt-bub1+-ymEGFP-427nt*
SW235	*h-*	*leu1+<<pDUAL-Pmad1-mad1+-ymEGFP*
SW235'	*h-*	*leu1+<<pDUAL-Pmad1-mad1+-ymEGFP*
SW241	*h-*	*sep1+-ymEGFP<<hphNT1*
SW241'	*h-*	*sep1+-ymEGFP<<hphNT1*
SW242	*h-*	*SPAC2H10.01+-ymEGFP<<hphNT1*
SW243	*h-*	*SPAC27D7.09c+-ymEGFP<<hphNT1*
SW244	*h-*	*leu1+<<pDUAL-Pbub1L-bub1+-ymEGFP*
SW244'	*h-*	*leu1+<<pDUAL-Pbub1L-bub1+-ymEGFP*
SW245	*h-*	*leu1+<<pDUAL-Pbub1Lmut1-bub1+-ymEGFP*
SW245'	*h-*	*leu1+<<pDUAL-Pbub1Lmut1-bub1+-ymEGFP*
SW249	*h+*	*GFP-mad1+*
SW253	*h+*	*leu1 his7+<<GFP-lacI-NLS but2+<<LEU2+<<lacO<<trz2+*
SW254	*h+*	*leu1 his7+<<GFP-lacI-NLS hrd3+<<LEU2+<<lacO<<toa1+*
SW509	*h-*	*leu1<<13nt-mad2+-ymEGFP-521nt*
SW510	*h-*	*leu1<<348nt-mad2+-ymEGFP-521nt*
SW511	*h-*	*leu1<<231nt-mad2+-ymEGFP-521nt*
SW513	*h+*	*wis1+<<110nt-mad1+-ymEGFP-164nt*
SW514	*h-*	*leu1<<58nt-mad2+-ymEGFP-521nt*
SW642	*h+*	*mad1+-ymEGFP*
SW702	*h+*	*wis1+<<58nt-Pmad2-rad21+-ymEGFP-521nt*
SW703	*h+*	*wis1+<<58nt-Pmad2-nmt1+-ymEGFP-521nt*
SW704	*h-*	*leu1+<<pDUAL-Pmad1mut1-mad1+-ymEGFP*
SW704'	*h-*	*leu1+<<pDUAL-Pmad1mut1-mad1+-ymEGFP*
SW705	*h-*	*leu1+<<pDUAL-Pmad1mut2-mad1+-ymEGFP*
SW705'	*h-*	*leu1+<<pDUAL-Pmad1mut2-mad1+-ymEGFP*
SW712	*h+*	*wis1+<332nt-Pmad3-nmt1+-ymEGFP-279nt*
SW714	*h+*	*wis1+<332nt-Pmad3-rad21+-ymEGFP-279nt*
SX030	*h+*	*ade6-M216*
SX430	*h+*	*mad1Δ::ymEGFP mad2Δ::ura4+ bub1+-ymEGFP*
SX432	*h+*	*ade6-M216 (ura4DS/E) bub1Δ::ura4+ mad1Δ::ymEGFP*
SX434	*h+*	*(ura4-D18) mad2Δ::ymEGFP mad3Δ::ura4+ bub1+-ymEGFP*

Table S23. sgRNA targeting sequences

Gene	Targeting sequence
mad2	ATTGGGTAGACAGTGACCCT
mad2	TATTCTTCATTAAGTTAGCA
mad2	TGTTGTTCTATTATCACTTT
mad3	GCAATTTACTCACCGTTGGT
wis1 locus intergenic	GTATGTGGCATACGCAGCCG
leu1 locus intergenic	GTAAGTACACAGCGACAACT
hph	TGCTCCATACAAGCCAACCA

Table S24. FISH probes

ymEGFP	*mad1*	*rpb1*	*cdc13*
cagtgaataattcttcacctt	gaacggatccctaggagaatc	cgtaaggggacagaagaaggt	aactcgtcaagatgctggtt
tcaaccaaaattgggacaaca	aatctaggcaactgtgaacgc	cggggacaagattccaaattg	taggggaaaagtcctgttct
gaccattaacatcaccatcta	atttggtttcttaacgcttgt	cgcaacgctcattgaacgaat	tctagctctagacagtgact
ccttcaccggagacagaaaat	gcagaattaacagaaggcttc	ttcatccatggtctcaggaaa	ggcaagagcttttgagactt
gtcaatttaccgtaagtagca	agctagtttgggatttttttgt	ggtttgacacttgaattgtcg	atagcctttgaccgaatcta
atggaactggcaatttaccag	ccttccgctttaaatcatttt	aaaatgaccaggacaatccgc	aacgtgaaggggtagtacgg
aaagtagtgactaaggttggc	actcaatttgttcacgctcaa	aactggctttgcaagttcaat	acgaattctgtcggggattg
tgttgtttcatatgatctggg	ttcttctgcaagttttctttg	ttttgcttaggaaaccgatgt	taggtgctggcgagttaaac
ctggcatggcagacttgaaaa	gctgtaacgaattcttctgtt	cgcaattccaacaaacgcatt	tttttgcacgggcaatatgg
gttctttcttgaacataacct	agttgcttttcaactagagtt	gatcacgataacgttgtgtgt	gaagacacagtggctttctt
agttaccgtcatctttgaaaa	gataagaagtagactgctcct	cagacattccaaactgcattt	caacgcatgacgcttcttag
ttgacttcagctctggtcttg	ccttttcatttcttacttctt	gtatcgcaaaccatttttgtc	agtgtgtctgacatttgtgt
taactaaggtatcaccttcaa	ttgcatcaagtagttcatgga	aaattatctgagcctgcagat	cacgacgggtactgacagaa
ataccttttaattcgattcta	ttcaactctgcaatctctttc	tattggcggagggattactaa	tcatctgttgcagggattat
cctaaaatgttaccatcttct	gatcattcttttctatctggg	tctttacgtattgtaggttgg	aaacagaaggttggcgacgc
gagagttatagttgtattcca	acttcatgattcttttcactt	caagaaccccataatctcagg	ttgaggtaacgaggggactg
gtcagccatgatgtaaacatt	tgcaaagcttgatttgagacc	aatctgattcatctttaccgc	gttgcttgatccttatggaa
actttgataccattcttttgt	gtttgtaagattggtatcctt	cgacagaaggcgtttttctgg	cgcgttcatcaacatctttc
gttgtgtctaattttgaagtt	ggaatccgcaaagagtttttc	atgtgtgaatattgtgtgcac	catcccaatcttgagattcg
attgaacagaaccatcttcaa	ttccttacacttcgtttcaag	actgctcattaagacctaagt	agggcatagtttcaatttcc
ttttgttgataatggtcagct	gaaagctcttgtaattgctgc	aataatcatccagtcaggtct	aagctctttttgacgatcca
agactggaccatcaccaattg	tcttccaattgctgattatga	cggaggaggaacaggtaagac	ccaatcggtaagtattccac
aagtaatggttgtctggtaac	aactagaaacctgcttgatgg	accgaaatactaggacggaca	aacaaggcagcaatgccaac
ggataaacgagattgagtgga	gcatttatttttttccagttca	ttaaatcatcttcgccacgac	ccatccgccatatatacaaa
ctctcttttcgtttggatctt	cgctaatttgaagacgttgct	aacgttggcatttgcttttat	tcggcttgaagaatttcctc
aattctaacaagaccatgtgg	cgcagcttttaatttttccaa	ttcagagacaatatgcgcagg	tcataggattcgggtaagca
ggtaataccagcagcagtaac	gatagcttttcaattcgttct	tggcaacatggaactgaagta	agtcggcttttgagatacga
ttgtacaattcatccatacca	ccttgagaatttcaacattcc	cgctatttcattgtccatata	ctgttgggaagggggataag
	ttggactccaaatcgtttttc	tctgccagatttttgtaaagc	ctttttggaggcatacttct
	aaccttatccctatattcttc	cgtgcacggatacttttaaga	aatcttcatcagcgtcatcg
	tcattttcgagttcaagggta	gagctgaaaaatcaacccgtt	gaattatcggcgactcgttt
	gggttggtaactcgttagtaa	aatttggatcaccggtaatca	tatgcatctggcaatcttca
	acgagtttgtttgaaacagct	aacaccgagttcatctaacga	atcggcaagtagctcaacat
	ttagcattggtattctgtaga	agtgtcttagcaatactacgt	atattactccgactcatggt
	taaactagaaacgcgctctcc	aggtgtaacagtttcaggata	

Table S25. Primers for quantitative PCR

Target	Forward	Reverse
act1	CCAAATCCAACCGTGAGAAGA	GTACGACCAGAGGCATACAAAG
cdc2	GGTATCGTGCTCCTGAAGTATTG	CAGAGTCACCGGGAAATAATGG
mad1	CCTAATGGGAGTGTTCGTGTTA	CCTGATGGATTACCAACCAATTTC
mad2	TTAGAGCGGTGGCAGTTTAAT	CTCGCAGTTCATCTTCTTTGTTG
mad3	CGGATGGTTCTGGAAAGGAT	CTACCCAAGAAGTAGCCGATATG
bub1	TGTCACCAGCTATGCCTAAAG	TCGTGGCTACCGGATTACTA
ymEGFP	TGAAGGTGAAGGTGATGCTAC	CTAAGGTTGGCCATGGAACT

Data S1. (separate file)
qPCR results for correlation analysis in Fig. 1A

Data S2. (separate file)
Counts of RNA per cell from single-molecule RNA FISH experiments (transcription site not labeled)

Data S3. (separate file)
qPCR results and immunoblot quantification for promoter mapping experiments in Fig. 4A,B, S13A.

Data S4. (separate file)
qPCR results and immunoblot quantification for candidate TATA box deletion experiments in Fig. 4D,E; S13B.

Data S5. (separate file)
Counts of RNA per cell from single-molecule RNA FISH experiments with transcription site labeling (Fig. 7, S18, S19)

REFERENCES AN NOTES

1. M. Kærn, T. C. Elston, W. J. Blake, J. J. Collins, Stochasticity in gene expression: From theories to phenotypes. *Nat. Rev. Genet.* **6**, 451–464 (2005).

2. A. Raj, A. van Oudenaarden, Nature, nurture, or chance: Stochastic gene expression and its consequences. *Cell* **135**, 216–226 (2008).

3. D. Zenklusen, D. R. Larson, R. H. Singer, Single-RNA counting reveals alternative modes of gene expression in yeast. *Nat. Struct. Mol. Biol.* **15**, 1263–1271 (2008).

4. A. Raj, C. S. Peskin, D. Tranchina, D. Y. Vargas, S. Tyagi, Stochastic mRNA synthesis in mammalian cells. *PLOS Biol.* **4**, e309 (2006).

5. I. Golding, J. Paulsson, S. M. Zawilski, E. C. Cox, Real-time kinetics of gene activity in individual bacteria. *Cell* **123**, 1025–1036 (2005).

6. K. B. Halpern, S. Tanami, S. Landen, M. Chapal, L. Szlak, A. Hutzler, A. Nizhberg, S. Itzkovitz, Bursty gene expression in the intact mammalian liver. *Mol. Cell* **58**, 147–156 (2015).

7. N. Battich, T. Stoeger, L. Pelkmans, Control of transcript variability in single mammalian cells. *Cell* **163**, 1596–1610 (2015).

8. M. Saint, F. Bertaux, W. Tang, X.-M. M. Sun, L. Game, A. Köferle, J. Bähler, V. Shahrezaei, S. Marguerat, Single-cell imaging and RNA sequencing reveal patterns of gene expression heterogeneity during fission yeast growth and adaptation. *Nat. Microbiol.* **4**, 480–491 (2019).

9. D. Fraser, M. Kærn, A chance at survival: Gene expression noise and phenotypic diversification strategies. *Mol. Microbiol.* **71**, 1333–1340 (2009).

10. H. B. Fraser, A. E. Hirsh, G. Giaever, J. Kumm, M. B. Eisen, Noise minimization in eukaryotic gene expression. *PLOS Biol.* **2**, e137 (2004).

11. B. Lehner, Selection to minimise noise in living systems and its implications for the evolution of gene expression. *Mol. Syst. Biol.* **4**, 170 (2008).

12. M. Sun, J. Zhang, Allele-specific single-cell RNA sequencing reveals different architectures of intrinsic and extrinsic gene expression noises. *Nucleic Acids Res.* **48**, 533–547 (2019).

13. J. Rodriguez, D. R. Larson, Transcription in living cells: Molecular mechanisms of bursting. *Annu. Rev. Biochem.* **89**, 1–24 (2020).

14. K. Bahar Halpern, I. Caspi, D. Lemze, M. Levy, S. Landen, E. Elinav, I. Ulitsky, S. Itzkovitz, Nuclear retention of mRNA in mammalian tissues. *Cell Rep.* **13**, 2653–2662 (2015).

15. M. M. K. Hansen, R. V. Desai, M. L. Simpson, L. S. Weinberger, Cytoplasmic amplification of transcriptional noise generates substantial cell-to-cell variability. *Cell Syst.* **7**, 384–397.e6 (2018).

16. A. Sanchez, I. Golding, Genetic determinants and cellular constraints in noisy gene expression. *Science* **342**, 1188–1193 (2013).

17. V. Shahrezaei, P. S. Swain, Analytical distributions for stochastic gene expression. *Proc. Natl. Acad. Sci. U.S.A.* **105**, 17256–17261 (2008).

18. M. Thattai, A. van Oudenaarden, Intrinsic noise in gene regulatory networks. *Proc. Natl. Acad. Sci. U.S.A.* **98**, 8614–8619 (2001).

19. D. R. Larson, D. Zenklusen, B. Wu, J. A. Chao, R. H. Singer, Real-time observation of transcription initiation and elongation on an endogenous yeast gene. *Science* **332**, 475–478 (2011).

20. M. Thattai, Universal poisson statistics of mRNAs with complex decay pathways. *Biophys. J.* **110**, 301–305 (2016).

21. J. M. Pedraza, J. Paulsson, Effects of molecular memory and bursting on fluctuations in gene expression. *Science* **319**, 339–343 (2008).

22. S. J. Gandhi, D. Zenklusen, T. Lionnet, R. H. Singer, Transcription of functionally related constitutive genes is not coordinated. *Nat. Struct. Mol. Biol.* **18**, 27–34 (2010).

23. X.-M. Sun, A. Bowman, M. Priestman, F. Bertaux, A. Martinez-Segura, W. Tang, C. Whilding, D. Dormann, V. Shahrezaei, S. Marguerat, Size-dependent increase in RNA polymerase II initiation rates mediates gene expression scaling with cell size. *Curr. Biol.* **30**, 1217–1230.e7 (2020).

24. Y. Taniguchi, P. J. Choi, G.-W. Li, H. Chen, M. Babu, J. Hearn, *A. Emili*, X. S. Xie, Quantifying *E. coli* proteome and transcriptome with single-molecule sensitivity in single cells. *Science* **329**, 533–538 (2010).

25. R. Foreman, R. Wollman, Mammalian gene expression variability is explained by underlying cell state. *Mol. Syst. Biol.* **16**, e9146 (2020).

26. P. S. Swain, Efficient attenuation of stochasticity in gene expression through post-transcriptional control. *J. Mol. Biol.* **344**, 965–976 (2004).

27. A. Singh, Negative feedback through mRNA provides the best control of gene-expression noise. *IEEE Trans. Nanobioscience* **10**, 194–200 (2011).

28. J. Mäkelä, M. Kandhavelu, S. M. D. Oliveira, J. G. Chandraseelan, J. Lloyd-Price, J. Peltonen, O. Yli-Harja, A. S. Ribeiro, In vivo single-molecule kinetics of activation and subsequent activity of the arabinose promoter. *Nucleic Acids Res.* **41**, 6544–6552 (2013).

29. A. F. Ramos, J. E. M. Hornos, J. Reinitz, Gene regulation and noise reduction by coupling of stochastic processes. *Phys. Rev. E* **91**, 020701 (2015).

30. S. Choubey, J. Kondev, A. Sanchez, Deciphering transcriptional dynamics in vivo by counting nascent RNA molecules. *PLOS Comput. Biol.* **11**, e1004345 (2015).

31. M. Sturrock, S. Li, V. Shahrezaei, The influence of nuclear compartmentalisation on stochastic dynamics of self-repressing gene expression. *J. Theor. Biol.* **424**, 55–72 (2017).

32. M. Z. Ali, S. Choubey, D. Das, R. C. Brewster, Probing mechanisms of transcription elongation through cell-to-cell variability of RNA polymerase. *Biophys. J.* **118**, 1769–1781 (2020).

33. R. Karmakar, A. K. Das, Effect of transcription reinitiation in stochastic gene expression. *J. Stat. Mech.* **2021**, 033502 (2021).

34. A.-B. Muthukrishnan, M. Kandhavelu, J. Lloyd-Price, F. Kudasov, S. Chowdhury, O. Yli-Harja, A. S. Ribeiro, Dynamics of transcription driven by the tetA promoter, one event at a time, in live *Escherichia coli* cells. *Nucleic Acids Res.* **40**, 8472–8483 (2012).

35. M. Kandhavelu, J. Lloyd-Price, A. Gupta, A.-B. Muthukrishnan, O. Yli-Harja, A. S. Ribeiro, Regulation of mean and noise of the in vivo kinetics of transcription under the control of the lac/ara-1 promoter. *FEBS Lett.* **586**, 3870–3875 (2012).

36. T. Lionnet, B. Wu, D. Grünwald, R. H. Singer, D. R. Larson, Nuclear physics: Quantitative single-cell approaches to nuclear organization and gene expression. *Cold Spring Harb. Symp. Quant. Biol.* **75**, 113–126 (2010).

37. S. Braichenko, J. Holehouse, R. Grima, Distinguishing between models of mammalian gene expression: Telegraph-like models versus mechanistic models. *J. R. Soc. Interface* **18**, 20210510 (2021).

38. S. Heinrich, E.-M. Geissen, J. Kamenz, S. Trautmann, C. Widmer, P. Drewe, M. Knop, N. Radde, J. Hasenauer, S. Hauf, Determinants of robustness in spindle assembly checkpoint signalling. *Nat. Cell Biol.* **15**, 1328–1339 (2013).

39. E. Esposito, D. E. Weidemann, J. M. Rogers, C. M. Morton, E. K. Baybay, J. Chen, S. Hauf, Mitotic checkpoint gene expression is tuned by codon usage bias. *EMBO J.* **41**, e107896 (2022).

40. E. S. Fischer, Kinetochore-catalyzed MCC formation: A structural perspective. *IUBMB Life* **75**, 289–310 (2022).

41. A. D. McAinsh, G. J. P. L. Kops, Principles and dynamics of spindle assembly checkpoint signalling. *Nat. Rev. Mol. Cell Bio*, 1–17 (2023).

42. E. Chung, R.-H. Chen, Spindle checkpoint requires Mad1-bound and Mad1-free Mad2. *Mol. Biol. Cell* **13**, 1501–1511 (2002).

43. S. D. Ryan, E. M. C. Britigan, L. M. Zasadil, K. Witte, A. Audhya, A. Roopra, B. A. Weaver, Up-regulation of the mitotic checkpoint component Mad1 causes chromosomal instability and resistance to microtubule poisons. *Proc. Natl. Acad. Sci. U.S.A.* **109**, E2205–E2214 (2012).

44. M. Öztürk, A. Freiwald, J. Cartano, R. Schmitt, M. Dejung, K. Luck, B. Al-Sady, S. Braun, M. Levin, F. Butter, Proteome effects of genome-wide single gene perturbations. *Nat. Commun.* **13**, 6153 (2022).

45. A. Raj, P. van den Bogaard, S. A. Rifkin, A. van Oudenaarden, S. Tyagi, Imaging individual mRNA molecules using multiple singly labeled probes. *Nat. Methods* **5**, 877–879 (2008).

46. O. Padovan-Merhar, G. P. Nair, A. G. Biaesch, A. Mayer, S. Scarfone, S. W. Foley, A. R. Wu, S. L. Churchman, A. Singh, A. Raj, Single mammalian cells compensate for differences in cellular volume and DNA copy number through independent global transcriptional mechanisms. *Mol. Cell.* **58**, 339–352 (2015).

47. J. Zhurinsky, K. Leonhard, S. Watt, S. Marguerat, J. Bähler, P. Nurse, A coordinated global control over cellular transcription. *Curr. Biol.* **20**, 2010–2015 (2010).

48. H. Kempe, A. Schwabe, F. Crémazy, P. J. Verschure, F. J. Bruggeman, The volumes and transcript counts of single cells reveal concentration homeostasis and capture biological noise. *Mol. Biol. Cell* **26**, 797–804 (2015).

49. M. Thodberg, A. Thieffry, J. Bornholdt, M. Boyd, C. Holmberg, A. Azad, C. T. Workman, Y. Chen, K. Ekwall, O. Nielsen, A. Sandelin, Comprehensive profiling of the fission yeast transcription start site activity during stress and media response. *Nucleic Acids Res.* **47**, 1671–1691 (2018).

50. M. Kozak, A short leader sequence impairs the fidelity of initiation by eukaryotic ribosomes. *Gene Expr.* **1**, 111–115 (1991).

51. A. J. Faure, J. M. Schmiedel, B. Lehner, Systematic analysis of the determinants of gene expression noise in embryonic stem cells. *Cell Syst.* **5**, 471–484.e4 (2017).

52. G. Hornung, R. Bar-Ziv, D. Rosin, N. Tokuriki, D. S. Tawfik, M. Oren, N. Barkai, Noise–mean relationship in mutated promoters. *Genome Res.* **22**, 2409–2417 (2012).

53. D. L. Jones, R. C. Brewster, R. Phillips, Promoter architecture dictates cell-to-cell variability in gene expression. *Science* **346**, 1533–1536 (2014).

54. W. J. Blake, G. Balázsi, M. A. Kohanski, F. J. Isaacs, K. F. Murphy, Y. Kuang, C. R. Cantor, D. R. Walt, J. J. Collins, Phenotypic consequences of promoter-mediated transcriptional noise. *Mol. Cell* **24**, 853–865 (2006).

55. S. Hocine, M. Vera, D. Zenklusen, R. H. Singer, Promoter-autonomous functioning in a controlled environment using single molecule FISH. *Sci. Rep.* **5**, 9934 (2015).

56. H. Li, J. Hou, L. Bai, C. Hu, P. Tong, Y. Kang, X. Zhao, Z. Shao, Genome-wide analysis of core promoter structures in *Schizosaccharomyces pombe* with DeepCAGE. *RNA Biol.* **12**, 525–537 (2015).

57. A. Matsuyama, A. Shirai, Y. Yashiroda, A. Kamata, S. Horinouchi, M. Yoshida, pDUAL, a multipurpose, multicopy vector capable of chromosomal integration in fission yeast. *Yeast* **21**, 1289–1305 (2004).

58. R. P. Birkenbihl, S. Subramani, The rad21 gene product of *Schizosaccharomyces pombe* is a nuclear, cell cycle-regulated phosphoprotein. *J. Biol. Chem.* **270**, 7703–7711 (1995).

59. J. Hayles, P. Nurse, Introduction to fission yeast as a model system. *Cold Spring Harb. Protoc.* **2018**, 323–333 (2017).

60. L. Krenning, S. Sonneveld, M. Tanenbaum, Time-resolved single-cell sequencing identifies multiple waves of mRNA decay during the mitosis-to-G1 phase transition. *Elife* **11**, e71356 (2022).

61. J. R. Peterson, J. A. Cole, J. Fei, T. Ha, Z. A. Luthey-Schulten, Effects of DNA replication on mRNA noise. *Proc. Natl. Acad. Sci. U.S.A.* **112**, 15886–15891 (2015).

62. V. V. Hausnerová, C. Lanctôt, Transcriptional output transiently spikes upon mitotic exit. *Sci Rep.* **7**, 12607 (2017).

63. I. Tsirkas, D. Dovrat, M. Thangaraj, I. Brouwer, A. Cohen, Z. Paleiov, M. M. Meijler, T. Lenstra, A. Aharoni, Transcription-replication coordination revealed in single live cells. *Nucleic Acids Res.* **50**, 2143–2156 (2022).

64. X. Fu, H. P. Patel, S. Coppola, L. Xu, Z. Cao, T. L. Lenstra, R. Grima, Quantifying how post-transcriptional noise and gene copy number variation bias transcriptional parameter inference from mRNA distributions. *eLife* **11**, e82493 (2022).

65. M. Wang, J. Zhang, H. Xu, I. Golding, Measuring transcription at a single gene copy reveals hidden drivers of bacterial individuality. *Nat. Microbiol.* **4**, 2118–2127 (2019).

66. M.-A. Saroufim, P. Bensidoun, P. Raymond, S. Rahman, M. R. Krause, M. Oeffinger, D. Zenklusen, The nuclear basket mediates perinuclear mRNA scanning in budding yeast. *J. Cell Biol.* **211**, 1131–1140 (2015).

67. P. Cramer, Organization and regulation of gene transcription. *Nature* **573**, 45–54 (2019).

68. G. T. Booth, I. X. Wang, V. G. Cheung, J. T. Lis, Divergence of a conserved elongation factor and transcription regulation in budding and fission yeast. *Genome Res.* **26**, 799–811 (2016).

69. L. Core, K. Adelman, Promoter-proximal pausing of RNA polymerase II: A nexus of gene regulation. *Genes Dev.* **33**, 960–982 (2019).

70. D. Cao, R. Parker, Computational modeling of eukaryotic mRNA turnover. *RNA* **7**, 1192–1212 (2001).

71. C. Deneke, R. Lipowsky, A. Valleriani, Complex degradation processes lead to non-exponential decay patterns and age-dependent decay rates of messenger RNA. *PLOS ONE* **8**, e55442 (2013).

72. H. Ochiai, T. Hayashi, M. Umeda, M. Yoshimura, A. Harada, Y. Shimizu, K. Nakano, N. Saitoh, Z. Liu, T. Yamamoto, T. Okamura, Y. Ohkawa, H. Kimura, I. Nikaido, Genome-wide kinetic properties of transcriptional bursting in mouse embryonic stem cells. *Sci. Adv.* **6**, eaaz6699 (2020).

73. E. Torre, H. Dueck, S. Shaffer, J. Gospocic, R. Gupte, R. Bonasio, J. Kim, J. Murray, A. Raj, Rare cell detection by single-cell RNA sequencing as guided by single-molecule RNA FISH. *Cell Syst.* **6**, 171–179.e5 (2018).

74. C. Mittal, O. Lang, W. K. M. Lai, B. F. Pugh, An integrated SAGA and TFIID PIC assembly pathway selective for poised and induced promoters. *Genes Dev.* **36**, 985–1001 (2022).

75. T. Nieuwkoop, M. Finger-Bou, J. van der Oost, N. J. Claassens, The ongoing quest to crack the genetic code for protein production. *Mol. Cell* **80**, 193–209 (2020).

76. J. Szavits-Nossan, R. Grima, Uncovering the effect of RNA polymerase steric interactions on gene expression noise: Analytical distributions of nascent and mature RNA numbers; https://doi.org/10.48550/arXiv.2304.05304 (2023).

77. M. Bertolini, K. Fenzl, I. Kats, F. Wruck, F. Tippmann, J. Schmitt, J. J. Auburger, S. Tans, B. Bukau, G. Kramer, Interactions between nascent proteins translated by adjacent ribosomes drive homomer assembly. *Science* **371**, 57–64 (2021).

78. A. Roguev, M. Wiren, J. S. Weissman, N. J. Krogan, High-throughput genetic interaction mapping in the fission yeast *Schizosaccharomyces pombe*. *Nat. Methods* **4**, 861–866 (2007).

79. J. Z. Jacobs, K. M. Ciccaglione, V. Tournier, M. Zaratiegui, Implementation of the CRISPR-Cas9 system in fission yeast. *Nat. Commun.* **5**, 5344–5344 (2014).

80. A. T. Watson, V. Garcia, N. Bone, A. M. Carr, J. Armstrong, Gene tagging and gene replacement using recombinase-mediated cassette exchange in *Schizosaccharomyces pombe*. *Gene* **407**, 63–74 (2008).

81. D. A. Zacharias, J. D. Violin, A. C. Newton, R. Y. Tsien, Partitioning of lipid-modified monomeric GFPs into membrane microdomains of live cells. *Science* **296**, 913–916 (2002).

82. J. Bähler, J. Q. Wu, M. S. Longtine, N. G. Shah, A. McKenzie, A. B. Steever, A. Wach, P. Philippsen, J. R. Pringle, Heterologous modules for efficient and versatile PCR-based gene targeting in *Schizosaccharomyces pombe*. *Yeast* **14**, 943–951 (1998).

83. S. Rohner, S. M. Gasser, P. Meister, Modules for cloning-free chromatin tagging in *Saccharomyces cerevisiae*. *Yeast* **25**, 235–239 (2008).

84. I. Arganda-Carreras, V. Kaynig, C. Rueden, K. W. Eliceiri, J. Schindelin, A. Cardona, H. S. Seung, Trainable weka segmentation: A machine learning tool for microscopy pixel classification. *Bioinformatics* **33**, 2424–2426 (2017).

85. F. Mueller, A. Senecal, K. Tantale, H. Marie-Nelly, N. Ly, O. Collin, E. Basyuk, E. Bertrand, X. Darzacq, C. Zimmer, FISH-quant: Automatic counting of transcripts in 3D FISH images. *Nat. Methods* **10**, 277–278 (2013).

86. K. Sewart, S. Hauf, Different functionality of Cdc20 binding sites within the mitotic checkpoint complex. *Curr. Biol.* **27**, 1213–1220 (2017).

87. H. Mi, A. Muruganujan, X. Huang, D. Ebert, C. Mills, X. Guo, P. D. Thomas, Protocol update for large-scale genome and gene function analysis with the PANTHER classification system (v.14.0). *Nat. Protoc.* **14**, 703–721 (2019).

88. M. A. Harris, K. M. Rutherford, J. Hayles, A. Lock, J. Bähler, S. G. Oliver, J. Mata, V. Wood, Fission stories: Using PomBase to understand *Schizosaccharomyces pombe* biology. *Genetics* **220**, iyab222 (2021).

89. D. Bates, M. Mächler, B. Bolker, S. Walker, Fitting linear mixed-effects models using lme4. *J. Stat. Softw.* **67**, 1–48 (2015).

90. T. Filatova, N. Popovic, R. Grima, Statistics of nascent and mature RNA fluctuations in a stochastic model of transcriptional initiation, elongation, pausing, and termination. *Bull Math Biol.* **83**, 3 (2021).

91. Z. Cao, T. Filatova, D. A. Oyarzún, R. Grima, A stochastic model of gene expression with polymerase recruitment and pause release. *Biophys. J.* **119**, 1002–1014 (2020).

92. D. Schnoerr, G. Sanguinetti, R. Grima, Approximation and inference methods for stochastic biochemical kinetics—A tutorial review. *J. Phys. Math. Theor.* **50**, 093001 (2017).

93. J. Elf, M. Ehrenberg, Fast evaluation of fluctuations in biochemical networks with the linear noise approximation. *Genome Res.* **13**, 2475–2484 (2003).

94. J. Peccoud, B. Ycart, Markovian modeling of gene-product synthesis. *Theor. Popul. Biol.* **48**, 222–234 (1995).

95. W. Shao, J. Zeitlinger, Paused RNA polymerase II inhibits new transcriptional initiation. *Nat. Genet.* **49**, 1045–1051 (2017).

96. S. Gressel, B. Schwalb, T. M. Decker, W. Qin, H. Leonhardt, D. Eick, P. Cramer, CDK9-dependent RNA polymerase II pausing controls transcription initiation. *eLife* **6**, e29736 (2017).

97. H. Xu, S. O. Skinner, A. M. Sokac, I. Golding, Stochastic kinetics of nascent RNA. *Phys. Rev. Lett.* **117**, 128101 (2016).

98. T. Zhou, J. Zhang, Analytical results for a multistate gene model. *Siam. J. Appl. Math.* **72**, 789–818 (2012).

99. B. Munsky, G. Li, Z. R. Fox, D. P. Shepherd, G. Neuert, Distribution shapes govern the discovery of predictive models for gene regulation. *Proc. Natl. Acad. Sci. U.S.A.* **115**, 7533–7538 (2018).

100. J. K. Pritchard, M. T. Seielstad, A. Perez-Lezaun, M. W. Feldman, Population growth of human Y chromosomes: A study of Y chromosome microsatellites. *Mol. Biol. Evol.* **16**, 1791–1798 (1999).

101. T. Toni, D. Welch, N. Strelkowa, A. Ipsen, M. P. H. Stumpf, Approximate Bayesian computation scheme for parameter inference and model selection in dynamical systems. *J. R. Soc. Interface* **6**, 187–202 (2008).

102. D. T. Gillespie, Exact stochastic simulation of coupled chemical reactions. *J. Phys. Chem.* **81**, 2340–2361 (1977).

103. E. Tankhilevich, J. Ish-Horowicz, T. Hameed, E. Roesch, I. Kleijn, M. P. H. Stumpf, F. He, GpABC: A Julia package for approximate Bayesian computation with Gaussian process emulation. *Bioinformatics* **36**, 3286–3287 (2020).

104. U. T. Eden, M. A. Kramer, Drawing inferences from Fano factor calculations. *J. Neurosci. Meth.* **190**, 149–152 (2010).

105. L. V. Ngoc, Y.-L. Wang, G. A. Kassavetis, J. T. Kadonaga, The punctilious RNA polymerase II core promoter. *Genes Dev.* **31**, 1289–1301 (2017).

106. V. Haberle, A. Stark, Eukaryotic core promoters and the functional basis of transcription initiation. *Nat. Rev. Mol. Cell Biol.* **19**, 1–17 (2018).

107. L. V. Ngoc, G. A. Kassavetis, J. T. Kadonaga, The RNA polymerase II core promoter in *Drosophila*. *Genetics* **212**, 13–24 (2019).

108. A. L. Roy, D. S. Singer, Core promoters in transcription: Old problem, new insights. *Trends Biochem. Sci.* **40**, 165–171 (2015).
